# Species Delimitation in Taxonomically Difficult Fungi: The Case of *Hymenogaster*

**DOI:** 10.1371/journal.pone.0015614

**Published:** 2011-01-25

**Authors:** Benjamin Stielow, Zoltan Bratek, Akos Kund I. Orczán, Szabolcs Rudnoy, Gunnar Hensel, Peter Hoffmann, Hans-Peter Klenk, Markus Göker

**Affiliations:** 1 DSMZ – German Collection of Microorganisms and Cell Cultures, Braunschweig, Germany; 2 Department of Plant Physiology and Molecular Plant Biology, ELTE University, Budapest, Hungary; 3 Fungarium Gunnar Hensel, Merseburg, Germany; Duke University Medical Center, United States of America

## Abstract

**Background:**

False truffles are ecologically important as mycorrhizal partners of trees and evolutionarily highly interesting as the result of a shift from epigeous mushroom-like to underground fruiting bodies. Since its first description by Vittadini in 1831, inappropriate species concepts in the highly diverse false truffle genus *Hymenogaster* has led to continued confusion, caused by a large variety of prevailing taxonomical opinions.

**Methodology:**

In this study, we reconsidered the species delimitations in *Hymenogaster* based on a comprehensive collection of Central European taxa comprising more than 140 fruiting bodies from 20 years of field work. The ITS rDNA sequence dataset was subjected to phylogenetic analysis as well as clustering optimization using OPTSIL software.

**Conclusions:**

Among distinct species concepts from the literature used to create reference partitions for clustering optimization, the broadest concept resulted in the highest agreement with the ITS data. Our results indicate a highly variable morphology of *H. citrinus* and *H. griseus*, most likely linked to environmental influences on the phenology (maturity, habitat, soil type and growing season). In particular, taxa described in the 19^th^ century frequently appear as conspecific. Conversely, *H. niveus* appears as species complex comprising seven cryptic species with almost identical macro- and micromorphology. *H. intermedius* and *H. huthii* are described as novel species, each of which with a distinct morphology intermediate between two species complexes. A revised taxonomy for one of the most taxonomically difficult genera of Basidiomycetes is proposed, including an updated identification key. The (semi-)automated selection among species concepts used here is of importance for the revision of taxonomically problematic organism groups in general.

## Introduction

The construction of a comprehensive phylogenetic classification of the fungi is a formidable task for modern systematics. As morphological characters alone are often insufficient for recognizing natural units, the analysis of nucleic acid sequences has significantly accelerated the progress towards achieving this challenging systematic goal. Particularly the taxonomy of the homobasidiomycetes has been entirely revolutionized by the use of molecular techniques, e.g. regarding the identification of (pseudo-)cryptic species and the specificity of mycorrhizal symbiosis [1], [2], [3]. The difficulties in defining characters and their states, and particularly the fact that distinct taxonomists assigned distinct weights to morphological characters, have probably been the largest obstacles to the establishment of broadly acceptable classifications of numerous difficult groups of fungi. Considerable morphological variation has been observed within genera and even within species [4], [5]. Hypogeous fungi, the morphologically defined group of mushrooms that form spores in enclosed basidio- and ascomata, are well suited to illustrate the intricacies involved in fungal taxonomy.

Traditional systematics has assigned these gasteroid taxa to a group of their own, but several studies have proven that at least some hypogeous basidiomycetes share morphological and genetic relations to epigeous sister taxa [6], [7], [8], [9]. Within the hebelomatoid-cortinarioid mushrooms only a few transition forms (so-called secotioid taxa) exist (e.g., *Thaxterogaster, Setchelliogaster*) in addition to several taxa with hymenothecia such as *Hebeloma*, *Naucoria*, *Gymnopilus* and *Cortinarius* [9], [10], [11], [12]. Among the hebelomatoid-cortinarioid fungi, the *Hymenogasteraceae* exclusively comprise hypogeous ectomycorrhizal taxa such as the genera *Timgrovea*, *Destuntzia* and *Hymenogaster*, which are dispersed in temperate and (sub-)tropical habitats [11], [12], [13], [14], [15]. Fruiting body morphology, spore ornamentation and ectomycorrhizal habit of these genera allowed their affiliation with the epigeous hebelomatoid-cortinarioid taxa [8], [16]. Molecular data confirmed that the hypogeous genus *Hymenogaster* is linked through secotioid forms to the epigeous mushrooms [8], [10].

Numerous authors [17], [18], [19], [20], [21], [22], [23] have reported *Hymenogaster* taxa from the southern hemisphere, mainly from Australia and New Zealand. With approximately 170 species within *Hymenogaster* (sensu lato) described from the northern and southern hemispheres and submitted to the MycoBank database [24], *Hymenogaster* is the most species-rich genus of false truffles. Since the first description of *Hymenogaster* was published by Vittadini [25], the generic limits of this heterogeneous, confusing assemblage of fungi were redefined several times [26], [27], [28], [29]. On several occasions taxa of false truffles with a brown-beige-greyish, loculate, non-gelatinized gleba completely enclosed within a peridium have incorrectly been placed within *Hymenogaster* [29], [30]. Quite a few authors have noted the strong morphological variability of *Hymenogaster* basidiomes [16], [25], [26], [31], [32], [33], [34], leading others to proposing doubtful infrageneric ranks [23], [35], [36]. As the most recent addition, *Cortinogaster* was suggested to constitute a novel genus closely related to *Cortinarius* but as yet remained unpublished and is currently termed “*Hymenogaster sublilacinus*” [8] in Genbank, which is not an authoritative source for taxonomy. Conversely, the merging of *Hymenogaster* and *Gautieria* [29] has not been confirmed by molecular studies [37], in which *Gautieria* appeared as more closely related to the gomphoid-phalloid fungi.

Even though organisms such as *Hymenogaster* display well-examined phenotypic characteristics, analysis of molecular data is apparently necessary to validate established species concepts and to identify those that require a taxonomic revision. Moreover, molecular data are essential to detect so-called cryptic species (or pseudocryptic species) [38], i.e. species for which no morphological differences exist (or have not been determined so far). Finally, molecular taxonomy is needed to analyse sequences directly sampled from the environment as, e.g., in the context of metagenomics projects [39], [40]. For molecular species delimitation, researchers mostly used a predefined threshold *T* for pairwise genetic distances in clustering algorithms to assign sequences to molecular operational taxonomic units [41], [42], [43], [44], [45], [46]. However, values of *T* used for clustering differ in the literature, even if applied to the same groups of organisms and molecular markers [47], [48], [49], [46], and are often based on subjective criteria or on a recently emerged tradition for the sake of comparability between studies [50], [51], [52]. In addition to *T*, the clustering algorithm also affects the circumscription and the shape of the clusters formed [53: p. 192]. In the context of linkage clustering, a link is defined as a pairwise distance shorter than or equal to the chosen threshold *T*. To add a new object to a given cluster, one can either request that at least one distance to a cluster member is a link (single linkage) or that all distances are links (complete linkage), or any proportion *F* of the distances between the new object and cluster members are links (see overview in [53]). However, *F* has hardly been addressed in the recent literature on molecular taxonomy [54]. For a given *T*, mean and maximum within-cluster distances may, but need not be much larger for small values of *F* [53: p. 192], thus potentially allowing a better adaptation to cases where genetic divergence differs between morphologically defined lineages [55]. Methods more advanced than linkage clustering have been suggested [56], [57], but these focus on identification, i.e. the assignment of query sequences to predefined groups, and thus require a correct reference taxonomy. However, misidentifications even of organisms with well-established microscopical characteristics are possible, and sequences in public databases are frequently mislabelled [58]. Thus, it is obvious that methods are needed that can adapt molecular taxonomy to reference data based on traditional taxonomy, without requiring that the latter is 100% correct.

A recently introduced method, clustering optimization [54], [59], allows one to obtain taxonomic units from non-hierarchical clustering that are in optimal agreement with a given reference dataset as, e.g., derived from traditional taxonomy. The parameters *T* and *F* are determined that result in the highest agreement between the clustering partition and the reference data, which are also represented as a partition (i.e., a non-overlapping, non-hierarchical division of objects such as a classification comprising a single taxonomic rank only). Moreover, optimizing clustering independently for distinct reference partitions created by applying distinct taxonomic concepts (as, e.g., represented in identification keys) allows one to select the taxonomic concept that results in the overall highest agreement with the molecular data. (In an analogous way, *via* independently optimizing distinct distance matrices, one could select the best distance functions for clustering sequences that are difficult to align [59].) The best concept as well as the best clusters obtained can then serve as the starting point for a taxonomic revision.

We here apply clustering optimization to a *Hymenogaster* internal transcribed spacer nuclear ribosomal DNA (ITS nrDNA) data matrix using distinct reference partitions representing distinct species concepts. The outcome is used to reconsider the species concepts for the Central European *Hymenogaster* spp. and to uncover several cryptic, novel, neglected and conspecific taxa. Our study is based on more than 140 specimens and represents the first comprehensive analysis on the genus *Hymenogaster* based on both morphological and molecular data. The File S1 includes an illustrated dichotomous key to the taxa of one of the most taxonomically difficult genera of *Basidiomycetes*.

## Materials and Methods

### Collecting and morphological study

Fruiting bodies were collected at various seasons during a period of approximately twenty years. Most of the Hungarian and Eastern European specimens were collected by ZB with the help of trained truffle dogs, mainly specialised on detecting commercially relevant black *Tuber* species. The material originating from Germany collected by GH and BS was dug out at suitable locations of different types of forest or park-like habitats. Collection details for the 142 dried and fresh basidiomata collected by the authors are provided in File S2.

Microscopical characteristics were observed for each collection (not necessarily for each fruiting body) on dried specimens mounted in 5% KOH (w/v). Tissue measurements were made with a 40x lens (Zeiss Axiophot) and repeated 10 times. Spores were measured using the software package Image Pro. For selected specimens (see below), mean measured length and width data were visualized as a dendrogram by calculating euclidean distances and applying average-linkage clustering [53] using the statistical software R (http://www.r-project.org/). Only the following exsiccates could not be measured: zb1485 (unripe), zb321, zb225 (the entire fruiting body was used for DNA extraction), zb20070605 and zb20070814 (the lent material had to be sent back to the herbarium). The morphological data were also used for species identification (see below) and the (informal) establishment of larger subgroups within *Hymenogaster* for comparison with the phylogenetic tree.

### DNA isolation, PCR and sequencing

Total genomic DNA from German specimens was extracted from approximately 50 mg basidioma material using the MasterPure Fungal Genomic DNA Kit following the manufacturer's protocol. DNA from fruiting body tissue of Hungarian exsiccates was extracted with the Qiagen DNeasy Plant Mini Kit following the manufactures protocol. The ITS rDNA region was amplified with the PCR primers ITS1F/ITS4 and ITS1/ITS4 under semi-nested conditions [60], [61] using Takara Hot Ex Taq and alternatively Fermentas Dream Taq. The PCR reactions were run with the following settings: initial denaturation for 3 min at 95°C followed by 35 cycles of 30 s denaturation at 95°C, annealing at 60°C (alternatively 51°C) for 30 s, extension for 1 min (alternatively 45 s) at 72°C and final extension at 72°C for 10 min (alternatively 7 min). The cycle sequencing reaction was conducted using the Beckman Coulter GenomeLab DTCS Quick Start Kit^M^ according to the manufacturer's protocol, followed by sequencing with the corresponding lab capillary electrophoresis system. Alternatively, PCR products were purified using Bioline Sure Clean following the manufacturer's instructions and sent to commercial sequencing labs. The sequences were assembled using Invitrogen Vector NTI 11.

### Phylogenetic analysis

Sequences obtained as described above were complemented using a classification-based search for *Hymenogaster* ITS sequences in GenBank (http://ncbi.nlm.nih.gov/). Sequences and taxonomic information to define the reference partitions (see below) were extracted from the complete GenBank flat files using the program gbk2fas [54] (freely available at http://www.goeker.org/mg/clustering/). Eighteen GenBank entries, termed as *H. brunnescens*, *H. diabolus*, *H. subcaeruleus*, *H. sublilacinus* or *H. subochraceus*, apparently assigned to an unpublished genus “*Cortinogaster*” were not taken into account because their affinity is closer to *Cortinarius* than to *Hymenogaster* [8]. The combined dataset comprising a total of 165 sequences was aligned with POA (version 2) in progressive alignment mode [62].

Phylogenetic analysis under the maximum-likelihood (ML) criterion [63] was conducted with RAxML version 7.2.5, using its fast bootstrap option with subsequent search for the best tree, employing the GTR+GAMMA model [64]. (See the RAxML manual for the rationale behind model choice.) Bootstrapping under the maximum-parsimony (MP) criterion [65] was done with PAUP* version 4.0b10 [66], treating gaps as missing data, collapsing branches of zero minimum length, and using 10 rounds of random sequence addition (in which only a single best tree was held, respectively) followed by TBR branch swapping per bootstrap replicate. In both ML and MP bootstrapping, 1000 replicates were conducted. Because, to the best of our knowledge, the sister group of *Hymenogaster* is uncertain and because the phylogenetic relationships between *Hymenogaster* and other genera are not of interest in the current study, the tree was rooted using midpoint rooting [67] as implemented in PAUP* to avoid the need for including outgroup taxa. Sequence alignments and phylogenetic trees are included in the supporting file S3.

### Identification of Hymenogaster and clustering optimization

For comparing the different species concepts for *Hymenogaster* from the literature, the specimens collected by GH and BS were determined using two distinct identification keys, one of which was modified according to a suggestion of its authors, yielding a total of three reference partitions for use in optimization (see below). First, the key from Montecchi and Sarasini [16] was used literally; only for few species which were not considered therein we resorted to Soehner [26]. Second, species were identified in the same manner but the following sets of species were merged as suggested by Montecchi and Sarasini [16] themselves: (i) *H. citrinus* and *H. olivaceus* (p. 491) and (ii) *H. hessei*, *H. lycoperdineus*, *H. populetorum* and *H. vulgaris* (p. 476, 483, 501). Third, the key of Soehner [26] was used. The key that performed best in clustering optimization (thus representing the most appropriate taxonomic concept) was then applied to identify the specimens collected by ZB (for pragmatic reasons, these were not re-identified using the suboptimal keys).

GenBank sequences and species affiliations were not used in clustering optimization because the underlying taxonomic concepts were unknown to us. Pairwise uncorrected (“p”) distances for clustering were calculated with PAUP*. Clustering optimization as implemented in OPTSIL [54] (freely available at http://www.goeker.org/mg/clustering/) was run independently for each of the three reference partitions, which corresponded to the three distinct species concepts listed above; four short sequences (it15_2, it15_3, it10_5_1 and it9_5_1) were not considered in distance calculation, yielding a set of 58 sequences for optimization (51 in the case of the third key because seven specimens could not be assigned to a species). *F* values were varied between 0.0 and 1.0, using a step width of 0.05. For each *F*, *T* was varied between 0.0 and 1.0, applying a step width of 0.0001. For each of the three reference partitions, we recorded the highest observed agreement with a clustering partition and the corresponding clustering parameters. The agreement is measured using the modified Rand index (MRI), which varies between −1.0 and 1.0; 1.0 represents full agreement, whereas random partitions achieve a MRI of about 0.0 (for details, see [54] and references therein). The clustering parameters yielding the globally highest MRI were applied to cluster the complete sequence set, including all collected specimens and all GenBank sequences.

As the optimal clustering does not necessarily include only clusters that appear as monophyletic in the optimal phylogeny, we used the SH-test as implemented in RAxML [64] to assess whether the best tree obtained if all clusters are constrained to be monophyletic was significantly worse than the globally best ML tree. The same was done in PAUP* [66] under the MP criterion, using the built-in KH-test and nonparametric test. MP settings were as described above, but saving up to ten best trees per replicate. We conducted 100 random sequence addition replicates under both clustering-constrained and unconstrained conditions and both ML and MP.

## Results

### Identification

Identification of the 62 specimens collected by GH and BS using the 1^st^ key resulted in twelve distinct species; using the 2^nd^ key, only eight species were observed. In contrast, applying the 3^rd^ key resulted in 24 distinct species and four specimens that could not be identified (see File S2). Morphological data also allowed us to classify the specimens into five sections based on principal spore characters, similar to Soehner's opinion [26]:

1. Spore surface smooth, no ornamentation; no perisporium.
2. Spore surface smooth, verrucose or partially smooth and partially verrucose; perisporium present in verrucose spores, absent in smooth spores.
3. Spore surface verrucose; perisporium present.
4. Spore surface thorny-verrucose; perisporium present.
5. Spore surface thorny-spiny; perisporium present.

Some of these groups also appeared as monophyletic in the phylogenetic tree (see below).

### Sequence alignment, phylogenetic analysis and clustering optimization

**Figure 1:**
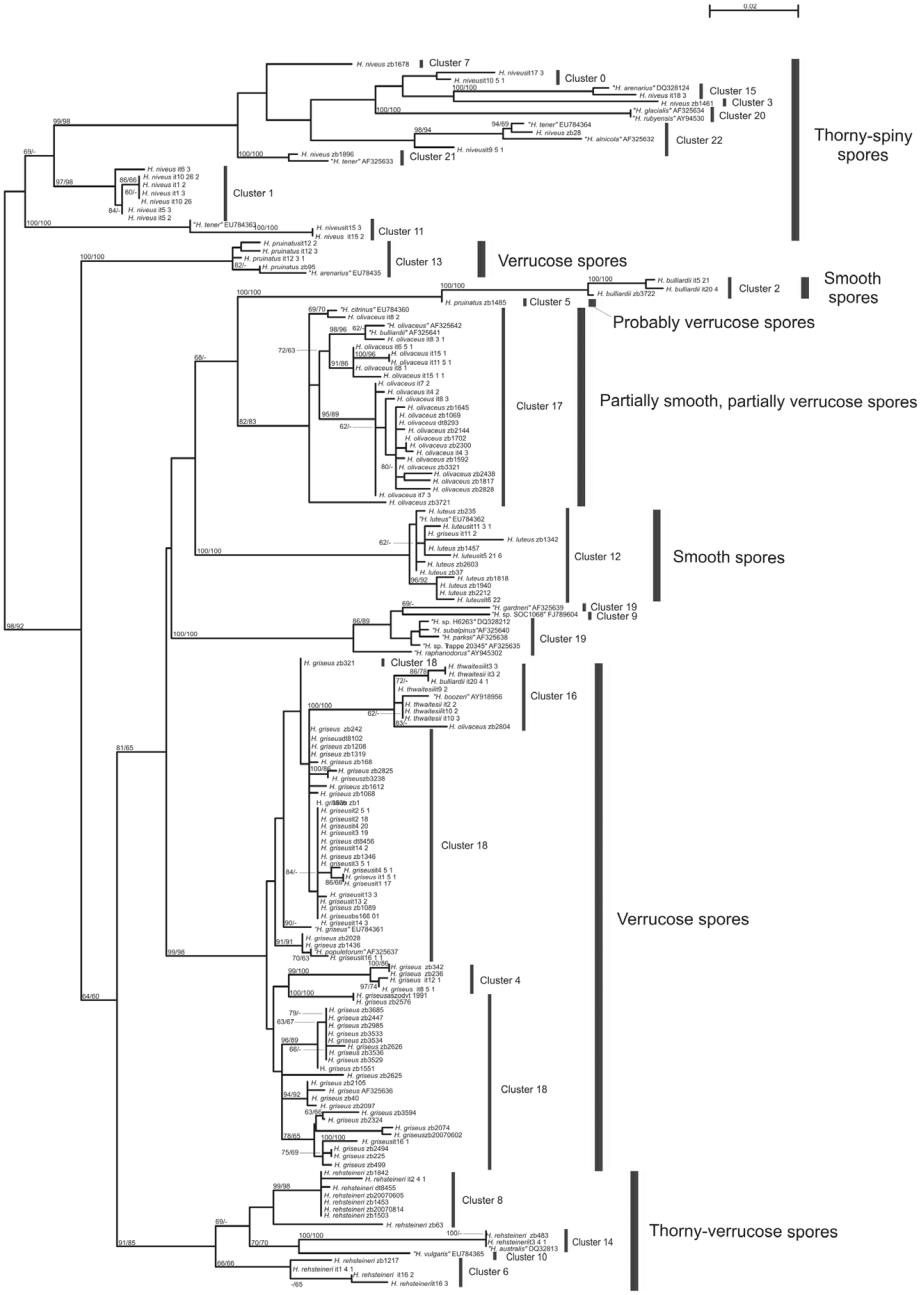
Phylogenetic tree inferred under the maximum-likelihood (ML) criterion from the ITS rDNA alignment and rooted using midpoint rooting. Numbers on the branches represent support values from 1,000 replicates under the ML (left) and the maximum-parsimony (right) criterion. The branches are scaled in terms of the expected number of substitutions per site. Accession numbers in the sequence labels indicate sequences from Genbank. Labels of sequences newly obtained in the course of this study include the assigned species name according to the best performing identification key [16] and the specimen ID (see File S2). Slender bars indicate the cluster membership according to the optimal clustering parameters applied to the optimal key. Wide bars indicate the five morphology-defined *Hymenogaster* species groups. (Specimen zb1485 was unripe and micromorphology had to be extrapolated from the macromorphological characteristics.)

The ITS rDNA alignment comprising 165 sequences had a total length of 983 positions, 221 of which were excluded because they mainly contained leading or trailing gaps. The resulting best ML tree had a log likelihood of −5311.323 and is shown in Fig. 1 together with ML and MP bootstrap values.

Results from clustering optimization using the ITS rDNA distance matrix and reference partitions inferred using three distinct identification keys are shown in Table 1. The 2^nd^ key, based on [16] but with some species merged, performed best, yielding a highest MRI of 0.814, which was obtained for *F* values between 0.65 and 1.0. The 1^st^ key, based directly on [16], performed moderately, yielding a maximal MRI of 0.642 with comparatively low settings for *F*. The 3^rd^ key, based on [26], performed even worse, yielding a maximum MRI of only 0.523. Consequently, we applied key 2 to identify the remaining specimens, and used the results for the annotation of the specimens in the tree (Fig. 1) and the clustering optimization with the entire dataset collected by ZB, BS and GH. An optimal MRI of 0.862 was obtained (Table 1) for values of *F* between 0.65 and 0.90. We chose *F* = 0.75 and the corresponding median optimal *T* of 0.0263 to cluster the complete dataset, including also the GenBank sequences, which yielded 23 clusters.

**Table 1:** Results from clustering-optimization when applying the three distinct identification keys to the BS/GH subset of the data (58 specimens) and applying the best performing key to the entire dataset (136 specimens).

F	Highest MRI	Median best T	Highest MRI	Median best T	Highest MRI	Median best T	Highest MRI	Median best T
0.00	0.64208	0.01200	0.77842	0.01500	0.51403	0.00075	0.81647	0.03490
0.05	0.64208	0.01200	0.77842	0.01505	0.51403	0.00075	0.82361	0.01695
0.10	0.64811	0.01120	0.77842	0.01650	0.51403	0.00075	0.81927	0.04165
0.15	0.64208	0.01270	0.77842	0.01655	0.51403	0.00075	0.82669	0.04495
0.20	0.64811	0.01195	0.77842	0.01655	0.52253	0.00235	0.82669	0.04550
0.25	0.64208	0.01275	0.77842	0.01655	0.52253	0.00235	0.82669	0.04560
0.30	0.64208	0.01350	0.77842	0.01660	0.52253	0.00235	0.85110	0.02010
0.35	0.61465	0.01270	0.77842	0.01675	0.52253	0.00235	0.85110	0.02030
0.40	0.61465	0.01270	0.77842	0.01745	0.52253	0.00235	0.86136	0.02195
0.45	0.63754	0.01275	0.77842	0.01755	0.52253	0.00235	0.85351	0.02210
0.50	0.63754	0.01275	0.77842	0.01820	0.52253	0.00235	0.85351	0.02260
0.55	0.62755	0.01275	0.77842	0.01965	0.52253	0.00235	0.85351	0.02300
0.60	0.63718	0.01440	0.77842	0.01965	0.52253	0.00235	0.86136	0.02350
0.65	0.61453	0.01980	0.81441	0.02220	0.45900	0.00235	0.86170	0.02550
0.70	0.61453	0.01980	0.81441	0.02225	0.45911	0.00395	0.86170	0.02575
0.75	0.61453	0.02055	0.81441	0.02285	0.45911	0.00395	0.86170	0.02630
0.80	0.61453	0.02065	0.81441	0.02300	0.45984	0.00710	0.86170	0.02670
0.85	0.61465	0.01735	0.81441	0.02320	0.45984	0.00710	0.86170	0.02775
0.90	0.61465	0.01735	0.81441	0.02390	0.45984	0.00710	0.86170	0.02915
0.95	0.62184	0.01835	0.81441	0.02620	0.45984	0.00710	0.82643	0.03045
1.00	0.62184	0.01835	0.81441	0.02805	0.46399	0.00630	0.81952	0.06640

Highest MRI values and corresponding median optimal *T* values are given for each examined *F* value.

Heuristic search under the ML criterion resulted in a best unconstrained tree with a log likelihood of −5311.487 and a best tree constrained for monophyly of all clusters from the optimal clustering with a log likelihood of −5318.039. According to the SH-test as implemented in RAxML, the latter tree was not significantly worse than the former (p = 0.05). Under MP, the globally best trees had a score of 710 steps in unconstrained and of 705 steps in constrained search; both the KH-test and the nonparametric test as implemented in PAUP* indicated to significant difference (p = 0.05). That is, while two clusters (18 and 19) do not appear as monophyletic in the tree, their monophyly is not significantly rejected by the data.

The cluster numbers are indicated in Fig. 1. Note that these numbers only indicate cluster membership; for instance, cluster 0 is not more closely related to cluster 1 than to cluster 22 or any other cluster. In the following, we will describe the composition of the clusters and their relationships to clades in the phylogenetic tree.

#### Clusters 0, 1, 3, 7, 11, 15, 20, 21 and 22

The clade composed of these clusters contains 24 sequences, all of which belong to specimens with thorny-spiny spores; after midpoint rooting, this clade appears as the sister group of all other examined *Hymenogaster* spp. (Fig. 1). Specimens in these clusters are uniformly identified as *H. niveus* except for some sequences from GenBank. All clusters correspond to clades (i.e., they are monophyletic in the tree), and most are highly supported. The clusters 0, 3, 7, 15, 20, 21 and 22 together form a clade; bootstrap support within this clade is low. The dendrogram inferred from the spore measurements did not indicate apparent differences between the *H. niveus* ITS clusters (data not shown; spore measurements are included in the electronic File S4).

#### Clusters 2, 5 and 13

The specimens identified as *H. pruinatus* occur in two distinct, not closely related clusters, which either comprise only a single specimen (cluster 5) or are highly supported as a clade (cluster 13). A single sequence from GenBank annotated as “*H. arenarius*” is also present in cluster 13. The sister-group relationship of the highly supported clades corresponding to cluster 5 and cluster 2 is also highly supported. Cluster 2 only contains specimens identified as *H. bulliardii*.

#### Cluster 17

This cluster corresponds to a moderately supported clade (82%/83% bootstrap support under ML/MP). It is a taxonomically rather consistent cluster, containing 27 sequences identified as *H. olivaceus* except for the GenBank sequences AF325641 (“*H. bulliardii*”) and EU784360 (“*H. citrinus*”).

#### Cluster 12

Except for one specimen, *H. griseus* it11_2, the clade equivalent to cluster 12 is taxonomically uniform, comprising only specimens assigned to *H. luteus*; it is also highly supported (100%).

#### Clusters 4, 16 and 18

Together these clusters form a highly supported clade that comprises the *Hymenogaster* specimens with verrucose spores (Fig. 1). Clusters 4 and 16 correspond to highly supported monophyletic assemblages inserted within the paraphyletic cluster 18. However, the non-monophyly of cluster 18 does not receive high bootstrap support, as the backbone within the entire clade is hardly supported. This is in accordance with the results of the SH- and KH-tests (see above). Using the modified key of Montecchi and Sarasini [16], clusters 4 and 18 are taxonomically uniform, including only specimens identified as *H. griseus*. Comprising 57 sequences, the clade corresponding to cluster 18 is the largest in the dataset. The subgroups within this clade did not appear to correlate with the origin of the specimens from certain geographical areas (Fig. 1, File S2). The specimens within cluster 16 are mostly identified as *H. thwaitesii* (six specimens), but the remaining ones as either *H. bulliardii* (it20_4_1), *H. olivaceus* (zb2804) or “*H. boozeri*” (AY918956).

#### Clusters 6, 8, 10 and 14

**Figure 2:**
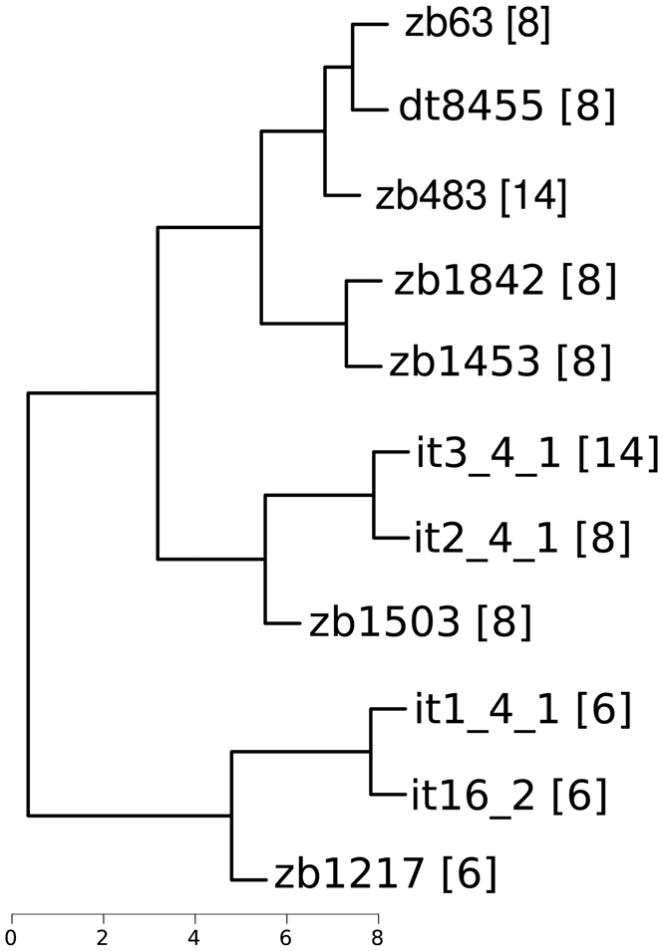
Dendrogram obtained by clustering the spore lengths and widths averaged for each specimen assigned to *H. rehsteineri*. Labels correspond to the specimens' ITS isolation numbers (see File S2), numbers in square brackets indicate the number of the cluster in Fig. 1. The branches are scaled in µm.

The clades corresponding to the clusters 6, 8, 10, 14 are, except for cluster 6, well-supported and together form a moderately well supported larger clade comprising the *Hymenogaster* collections with thorny-verrucose spores (Fig. 1). The four clades only contain specimens identified as *H. rehsteineri* except for two GenBank sequences assigned to either “*H. vulgaris*” (EU784365) or “*H. australis*” (DQ328132). The dendrogram inferred from the spore measurements (Fig. 2) indicates that cluster 6 may well be differentiated from the other two clusters, whereas clusters 8 and 14 are intermixed. Spores of cluster 6 are shorter and, hence, characterized by a lower length/width ratio (see electronic File S4 and below).

#### Clusters 9 and 19

All sequences within the clades corresponding to clusters 9 and 19 originate from GenBank and, according to their annotation, originate from North American exsiccates. Cluster 9 appears as nested within cluster 19, and moderate bootstrap support is present for the paraphyly of the latter clade (but see the results of the SH- and KH-tests mentioned above). Taxonomically, clade 19 is maximally diverse.

## Discussion

Since Vittadini established *Hymenogaster* in 1831 [25], a variety of prevalent taxonomical concepts introduced large numbers of hardly identifiable taxa, often lacking information on diagnostic characters [26], [32], [68], [69], [16]. Accordingly, not only the generic limits of *Hymenogaster* have been questioned [14], [29], [35] but also the species limits within this difficult group of fungi were frequently challenged in the past. In 1962, Soehner [26] accepted no less than 94 species in his monograph, including the eight species of Vittadini [25], the 15 ones of the Tulasne brothers [31] and numerous ones of Hesse [32]. The results obtained with ITS sequences and OPTSIL analysis favour a much broader species concept for the genus, as the key yielding the lowest number of species also resulted in the highest agreement between molecular data and classification. Nevertheless, the optimal clustering also indicates that some species of Montecchi & Sarasini [16] need to be split. In the following, we briefly discuss to what extent the resulting taxa were morphologically interpretable. As the five sections based on spore characters (see above) were largely congruent with the phylogenetic tree (Fig. 1), our arrangement of the discussed taxa follows that classification.

The *Hymenogaster* collections with smooth spores (Fig. 1; clusters 2 and 12) form a paraphyletic group comprising two species, *H. luteus* and *H. bulliardii*, which are comparatively easy to identify and are not in conflict with the clustering (the difficult varieties of *H. luteus* [25], [26] do not concern us here). *H. “griseus”* it11_2 in cluster 12 appears to be a misidentification (compare cluster 18, which contains the vast majority of specimens identified as *H. griseus*).

The section with partially smooth, partially verrucose spores is represented by the species *H. citrinus* and *H. olivaceus*. *H. citrinus* is one of Vittadini's [25] eight species, described nearly 180 years ago. The very similar species *H. olivaceus* was also described by Vittadini and is known to occur in the same forests and soil types, but in early spring, not during summer like *H. citrinus*. It has frequently been suggested to merge the two species [16], [69], which is corroborated by our results. Gross et al. [69] interpreted *H. citrinus* as an abnormal form of *H. olivaceus*. That typical basidiomata of *H. citrinus* (sensu stricto) have been described only from calcareous, clayey soils might indicate that the soil type, in addition to the season, has an effect on the colour of the peridia. Moreover, the fact that *H. citrinus* develops a strong unpleasant gleba odour whereas *H. olivaceus* is characterized by a rather pleasant smell, might indicate seasonal differences in the bacterial fruiting body flora.

Verrucose spores occur in the majority of *Hymenogaster* species. Aside from the variability of basidiomata, the rich number of different shapes, graduations and nuances present in verrucose-spored taxa is the major reason why most verrucose-spored taxa are almost indistinguishable. Several very early species concepts [25], [31] have persisted in all present classifications without having been questioned more intensively, including the following taxa with verrucose spores: *H. decorus*, *H. griseus*, *H. hessei*, *H. lilacinus*, *H. lycoperdineus*, *H. muticus*, *H. populetorum*, *H. rehsteineri*, *H. thwaitesii*, *H. verrucosus* and *H. vulgaris*. In particular, taxa morphologically similar to the current description of *H. vulgaris* agree in almost any character (e.g. fusoid, papillate, warty and verrucose spores) with those of *H. griseus*, *H. decorus*, *H. lycoperdineus*, *H. populetorum*, *H. muticus*, and partially agree with *H. hessei*. Montecchi & Sarasini [16] regard these taxa as conspecific, a view which is supported by our findings, as all of them are placed in a single cluster, cluster 18 (Fig. 1). While some morphologically constant and distinct characters are present in these supposed species, such as the lilac-purple-brown coloured gleba of *H. populetorum* and the apically rounded, verrucose spores of *H. vulgaris*, all of these taxa share common characters; further examples are the lack of a true columella and the strongly compressed sterile gleba cells.

However, the merging of other taxa as suggested by [16] is in disagreement with our results. Comparison with the key in [26] shows that cluster 4 comprises specimens of *H. megasporus*, a taxon similar to *H. vulgaris* and *H. decorus*, but distinguishable by its exceedingly large spores and a distinct purple-coloured peridia and gleba [26]. *H. megasporus* turned out not to be conspecific to the *H. griseus* group, in contrast to the identification of the cluster 4 specimens as *H. griseus* according to [16] (Fig. 1). While *H. megasporus* could be regarded as an abnormal giant-spored form of *H. griseus*, Soehner's approach [26] to establish a species of its own is in higher agreement with the clustering of the ITS sequence data.

In contrast to *H. megasporus*, *H. thwaitesii* was accepted by Montecchi & Sarasini [16]. It here appears in a cluster of its own (cluster 16), supporting this view. The two distinctly annotated specimens in cluster 16, *H. “bulliardii”* it20_4_1 and *H. “olivaceus”* zb2804 (an immature specimen) most likely represent identification artefacts (compare cluster 2, which contains most *H. bulliardii* specimens, and cluster 17, which comprises most *H. olivaceus* collections).

The specimens of *H. rehsteineri* included in our sample, a species characterized by thorny-verrucose spores, occur in three distinct clusters (6, 8 and 14). Greenish nuances of thorny-spiny *H. niveus* basidiospores, sometimes found in *H. rehsteineri* exsiccates, but not in other *Hymenogaster* spp., occur in both species complexes; however, they do not appear as particularly closely related in the tree (Fig. 1). Soehner [26] noted that *H. rehsteineri* is common around all seasons and habitats and particularly emphasized the great morphological variability of this taxon. Soehner was unable to delineate varieties of this fungus, even though his collection comprised more that 150 *H. rehsteineri* exsiccates.

Clustering optimization indicates that (pseudo-)cryptic diversity exists within this species. Closer examination showed that cluster 6 differs markedly in the length-width ratio of their spores and peridial colour from other specimens of *H. rehsteineri*. Moreover, in the phylogenetic tree (Fig. 1), cluster 6 appears as the sister group of the remaining *H. rehsteineri* clusters. Accordingly, we propose the novel species *Hymenogaster intermedius*. Its small, warty-verrucose spores are similar to those of *H. tener* and *H. arenarius* (see below), whereas its pale brown-greyish peridia is similar to the one found in typical *H. rehsteineri* specimens; further details are given below. As we did not detect morphological differences between the remaining *H. rehsteineri* clusters, we regard them as indicative of cryptic diversity. It is suggested to consider this revised *H. rehsteineri* as a species complex with higher genetic diversity than in typical *Hymenogaster* species.

Within the *Hymenogaster* section with thorny-spiny spores, Soehner [26] included the species *H. albus* ( =  *Descomyces albus*), *H. arenarius*, *H. mutabilis*, *H. niveus*, *H. pusillus* and *H. tener*. Montecchi & Sarasini [16] regarded them as conspecific. Results from clustering optimization do not support their view (Fig. 1). Specimens assigned to *H. arenarius* sensu [26], [69] are located in a cluster of their own (cluster 1), whereas *H. niveus* sensu Soehner [26] is distributed among seven clusters (0, 3, 7, 15, 20, 21, 22). *H. arenarius* and *H. niveus* sensu Soehner [26] are easily distinguishable by their seasonal appearance in spring (*H. arenarius*) vs. in early summer to early autumn (*H. niveus*), by the rancid, garlic-like odour of *H. arenarius* vs the mild flowery one of *H. niveus* and, most importantly, by the greyish-brownish wrinkled peridia of *H. arenarius* in contrast to the snow-white, smooth surface found in *H. niveus*. Likewise, *H. tener* sensu Soehner appears in a cluster of its own (cluster 11). It is distinguishable by its seasonal preference to early spring, its plain white peridia and its strong odour from *H. niveus*.

The large number of remaining clusters which have to be assigned to *H. niveus* sensu stricto indicate cryptic diversity. We were unable to detect morphological differences between these clusters. The observed specimens usually possess the “snow-white” basidiomata typical of *H. niveus* specimens sensu Soehner. We thus do not attempt to taxonomically split *H. niveus* but suggest to consider it as a species complex with significantly higher genetic diversity than the typical *Hymenogaster* species.

A set of specimens located in a cluster of their own (cluster 13) have apparently been misidentified as *H. pruinatus*. Like *H. pruinatus*, these share a plain white, partially greyish-brown peridia with *H. niveus* sensu stricto but verrucose, strongly wrinkled basidiospores with *H. griseus*. However, other characters deviate from *H. pruinatus*. Thus, a novel species, *H. huthii*, is proposed for these specimens; details are given below.

### A revised classification of *Hymenogaster*

#### *Hymenogaster arenarius*

Tul. & C. Tul. 1844 emend. Stielow et al. 2010

Including: *Hymenogaster minusculus* Soehner 1924, *Hymenogaster pusillus* Berk. & Broome 1846.

Cluster: 1

*Hymenogaster arenarius*, a species originally erected by the Tulasne brothers in 1844, but recently proposed to not constitute a species of its own [16], is easily distinguishable by several constant macromorphological characters [34]. The greyish-brownish peridium of *H. arenarius* is never found in specimens of *H. niveus*, which display a typical “snow-white” smooth and silky surface of the fruiting body. The nearly identical spore characteristics were most likely responsible for the merging of *H. arenarius* and *H. niveus*. Our results suggest to separate the two taxa but to include *Hymenogaster pusillus* Berk. & Broome 1846 in *H. arenarius* (see File S2).

#### *Hymenogaster bulliardii*

Vittad. 1831

Cluster: 2

Our analysis supports the isolated position of the type species of *Hymenogaster*. In particular, the absence of a perisporium and hence a smooth spore surface combined with dark reddish-brown, roundish, compressed gleba cells, a dendroid columella and an unpleasant smell are stable indicators to easily separate *H. bulliardii* from other species with smooth spores. Its closest relatives (Fig. 1), *H. citrinus* and *H. luteus*, are also known to lack a perisporium and to display completely or almost smooth basidiospores.

#### *Hymenogaster citrinus*

Vittad. 1831 emend. Stielow et al. 2010

Including: *Hymenogaster bucholtzii* Soehner (1924), *Hymenogaster citrinus* Vittad. 1831 =  *Gautieria citrina* (Vittad.) Bougher & Castellano 1993 =  *Splanchnomyces citrinus* (Vittad.) Corda 1854, *Hymenogaster olivaceus* Vittad. 1831, *Hymenogaster tomentellus* R. Hesse 1891. Probably also including: *Hymenogaster sulcatus* R. Hesse 1891.

Cluster: 17

Merging *H. olivaceus* and *H. citrinus* is in disagreement with the traditional understanding of these two very old species [25], [26]. However, in addition to the evidence presented here, dozens of specimens collected by Bratek et al. (data not shown) show an intermediate morphology, as also noted by [69]. We prefer the name “*H. citrinus*” over “*H. olivaceus*”, which was introduced in the same study [25], only for reasons of alphabetical sorting. *H. sulcatus* is not present in our collection but it is presumably conspecific to *H. citrinus* because [26] classified *H. sulcatus* into the same morphological group as *H. citrinus* and *H. tomentellus* due to their similar basidiospore development and their yellow-ochraceous gleba.

#### *Hymenogaster griseus*

Vittad. 1831 emend. Stielow et al. 2010

Including: *Hymenogaster hessei* Soehner 1923, *Hymenogaster lycoperdineus* Vittad. 1831, *Hymenogaster populetorum* Tul. & C. Tul. 1843, *Hymenogaster vulgaris* Tul. & C. Tul. 1846. Probably also including: *Hymenogaster lilacinus* Tul. & C. Tul. 1843, *Hymenogaster muticus* Berk. & Broome 1848.

Cluster: 18

One of the taxonomically most remarkable results of our study is the evidence for the conspecificity of the above-mentioned taxa, even though Montecchi and Sarasini [16] already preferred a wider species concept for *H. lycoperdineus*, *H. muticus* and *H. griseus*. The acceptance of an emended species *H. griseus* implies an enormous morphological variability among the merged taxa. As either “*H. griseus*” or “*H. lycoperdineus*” has priority [26], we prefer the former only for reasons of alphabetical sorting. *H. lilacinus*, which was not present in our dataset, was classified by [26] into the morphological group of *H. lycoperdineus* and *H. populetorum* which are conspecific to *H. muticus* (also missing in our dataset), *H. vulgaris* and *H. hessei* according to [16].

#### *Hymenogaster huthii*

Stielow et al. 2010, sp. nov. [urn:lsid:indexfungorum.org:names:518624]

Cluster: 13

*H. huthii* is apparently an ultra-rare species, collected at only three sites during 20 years, once in Hungary and twice in Germany. Surprisingly, this species appears as a highly supported clade of its own, corresponding to cluster 13 (Fig. 1), not forming a monophyletic group with other species with verrucose spores. A set of evidently stable characters such as the gently corrugated, pale-whitish basidiomata, a dark brown, thick gleba at full maturity, and verrucose spores surrounded by two parallel walls, a unique combination within the *Hymenogaster* spp. with verrucose spores, distinguishes this taxon from the otherwise similar *H. pruinatus* (see description in [26]). In *H. pruinatus* when ripening the peridia turns from pale whitish (similar to *H. huthii*) to dark-yellow-brown. The peridia of mature *H. huthii* fruiting bodies remains pale whitish (image 74 in the electronic File S1), similar to *H. niveus* sensu stricto (images 34 and 35). Basidiospores of *H. pruinatus* are citriform, light-brown and transparent [26], much like in its sister group *H. bulliardii* (Fig. 1) whereas those of *H. huthii* are broad-elongated, strongly verrucose, dark-brown and non-transparent. *H. huthii* and *H. pruinatus* are, however, similar regarding the colouration of their gleba, which turns dark black-brown (image 34), and their pleasant, aromatic smell.

#### Etymology

Named in honour of the German mycologist Manfred Huth, who dedicated more than 50 years of his life to exploring fungi, particularly the genus *Hymenogaster*.

#### Latin description

Basidiomata 15–25 mm diam., rogusa, globosa usque ad subglobosam, cavernis ad internum in basidiomatibus maturis, sulcis praesentibus. Peridia unius coloris alba in novellis subfuscis labidibus irregulariter diffusis in novellisque et maturis, 150–300 µm crassa. Gleba alba, canescens, fuscescens, fiat pulla plena maturitate. Columella absens. Basidia cylindrica, brevissima, 14-15×7-7.5 µm, cum duo sterigmata. Sporae oblongae-ellipsoides, fusoides, papillatae, convexae, rotundae in apice, 16-29×9-21 µm, longitudine media 22 µm, latitudine media 13 µm (ornamentationem inclusive, sed sine sterigmatibus reliquis et papilla), ornamentatio verrucosa, roguissima, inversa, robigine fusca in KOH et in aqua. Odor farinosus. Holotypus hic designatus Stolberg, Saxonia-Anhaltinum, montes continui Harz, Biosphere Reserve Southern Harz, leg. G. Hensel & U. Täglich 13 Iulii MMVIII (Herb. Nr. 130708GH), det. G. Hensel & B. Stielow.

#### English description

Basidiomata 15–25 mm in diameter, wrinkled, globose to subglobose, with cavities leading to the inner side in mature basidiomes, gouges present. Peridia plain white when young, with irregularly dispersed brownish spots in young and mature basidiomes, 150–300 µm thick. Gleba white, turning grey or brown, becoming brown-black at full maturity. Columella absent. Basidia cylindrical, very short, 14-15×7-7.5 µm, with two sterigmata. Spores broad elongated-ellipsoid, fusoid, papillate, convex, rounded at the apex, 16-29×9-21 µm, 22×13 µm on average (including ornamentation, but without remnants of sterigmata and papilla), their ornamentation verrucose, strongly wrinkled, folded, dark rusty brown in KOH and water. Odour pleasant. Assigned holotype from Stolberg, Sachsen-Anhalt, Germany (Harz mountain range, Biosphere Reserve Southern Harz), leg. G. Hensel & U. Täglich on 13^th^ July 2008 (Herb. Nr. 130708GH), det. G. Hensel & B. Stielow.

#### Further details

Immature basidiomata hypogeous, ovoid, roundish, plain white, with brownish-greyish spots, no mycelia cord or rhizomorphs visible, 10–20 mm broad. Basidiome consistency is compact. Immature gleba plain white, with grey tones, sponge-like consistency, not compressed, apparently no direct colour changes when exposed to air. Trama: immature, white; mature, grey-brown-black. Glebal trama 35–40 µm thick, composed of 1–4 µm broad hyphae (at the septa), with large inflated globose to subglobose cells, between 6–8 µm thick. Subhymenium weakly developed, interwoven hyphae. Cystidia not present. Clamp connections not present. Basidia: Sterigmata ca. 1×0.5 µm in length, basidium walls less than 0.5 µm thick, basidia generally hyaline in KOH. No distinctive reaction to Melzer's reagent. Spores' length/width quotient: minimum 1.45, average 1.79, maximum 2.15. Ornamentation strongly verrucose, surrounded by two parallel walls. Papilla and sterigmata remnants about the same length, 1-2×1 µm.

#### Distribution, habitat and seasonal appearance

*Hymenogaster huthii* is only known from the three collection sites, mainly from mixed woodlands at different elevations. In close proximity, three ectomycorrhizal hosts were identified: collection 211008GH, *Tilia platyphyllos*; collection 130708GH, *Corylus avellana*; and collection zb95, *Alnus glutinosa*.

#### Collections examined

See File S2.

#### Holotype deposit

Botanische Staatssammlung Munich; corresponding curator: Dr. D. Triebel; accession number of the holotype: M-0156226.

#### Mycobank Number

MB 518624.

#### *Hymenogaster intermedius*

Stielow et al. 2010, sp. nov. [urn:lsid:indexfungorum.org:names:518623]

Cluster: 6

The micromorphological characters of this species are almost identical to those of *H. tener* and *H. arenarius*, but its macromorphological similarities to *H. rehsteineri* have led us to wrongly assign these specimens to *H. rehsteineri*. The species *H. intermedius* apparently shares morphological characters of two distinct species complexes. None of the described *Hymenogaster* species displays this combination of features, which is indicative of a novel species, a conclusion that is supported by the ITS rDNA data. Spores of *H. intermedius* are significantly shorter than those of *H. rehsteineri*, yielding a lower length/width ratio (Fig. 2; images 61–64 in electronic File S1). The basidiomata are, however, extremely similar (images 96–100). Only three collections of *H. intermedius* are known so far, one from Hungary and two from Germany.

#### Etymology

Named in accordance to its intermediate morphological characters between *Hymenogaster rehsteineri* on the one hand and *Hymenogaster niveus*, *H. arenarius* and *H. tener* on the other hand.

#### Latin description

Basidiomata 10–15 mm in diam., tuberosa, globosa usque ad subglobosam. Peridia unius coloris alba in novellis, mutantia in pallidam ochream in basidiomatibus maturis, maxime similaria coloratione typicae *H. rehsteineri*, mutatione colorationis lentissima, 200 µm crassa, pseudoparenchymata ad internum, insolvabiles a parenchymati glebae. Gleba alba in novellis mutans per colores violaceos, mutans in clarum calidum fuscum plena maturitate, sed permanens clariorum colorum typicalis *H. nivei* specimentis. Columella praesens, cum corda mycelia in fundamento. Basidia ampullacea, cylindrica, 17.5-20×5-7 µm, cum duo sterigmata. Sporae globosae usque ad subglobosas, ovoides; ornamentaione spinea-verrucosa; 10-14×6-9 µm, longitudine media 12 µm, latitudine media 8 µm (ornamentatione inclusive, sed sine sterigmatibus reliquis et papilla), sporae gilvae-fuscae in KOH et in aqua. Odor farinosus, similaris cucumi. Holotypus hic designatus Stolberg, Saxonia-Anhaltinum, montes continui Harz, Biosphere Reserve Southern Harz, in calli ad *Fagus sylvatica*, leg. G. Hensel, 13 Iulii MMVIII, (Herb. Nr. 060708GH), det. G. Hensel & B. Stielow.

#### English description

Basidiomata 10–15 mm in diameter, tuberous, globose to subglobose. Peridia plain white when young, very slowly turning to pale-ochraceous in mature basidiomes, very similar to the typical colouration of *H. rehsteineri*, 200 µm thick, turning pseudoparenchymatically inwards, not detachable from the gleba tissue. Gleba white when young, passing through gentle lilac tones, turning to brilliant warm-brown at full maturity, but remaining more light-coloured than in typical *H. niveus* specimens. Columella present, with mycelial cord at the base. Basidia flask-shaped, cylindrical, 17.5-20×5-7 µm, with two sterigmata. Spores globose to subglobose, ovoid; ornamentation gently thorny-spiny; spores 10-14×6-9 µm, 12×8 µm on average; (including ornamentation, but without sterigmata remnants and papilla); spores yellow-brown in KOH and water. Odour floury, cucumber-like. Assigned holotype from Stolberg, Sachsen-Anhalt, Germany (Harz mountain range, Biosphere Reserve Southern Harz), at forest trail at *Fagus sylvatica*, leg. G. Hensel G., 13^th^ July 2008 (Herb. Nr. 060708GH).

#### Further details

Immature basidiomata hypogeous, globose, tuberous, plain white, mycelial cord visible, 10–15 mm broad. Basidiome consistency is compact. Immature gleba not compressed, apparently no direct colour changes when exposed to air; very slow colour changes when basidiome is exposed to air. Mature gleba: Spore ripening process might be irregular, causing discolouration with brownish spots dispersed on the mature gleba. Gleba colouration stays brighter brown than in *H. niveus* with its darker reddish-brownish tones. Trama: Immature, white; mature, (light)-brown. Trama walls elongated and convoluted. Glebal trama 40–45 µm thick, composed of 2.5–6.25 µm broad hyphae (at the septum), with large inflated globose to subglobose, 4.5–7.5 µm thick cells. Subhymenium weakly developed, interwoven hyphae. Cystidia not present. Clamp connections not present. Basidia: Sterigmata ca. 2 µm long, basidial walls less than 0.8 µm thick, basidia hyaline in KOH. No distinctive reaction to Melzer's reagent. Spores' length/width quotient of spores: minimum 1.34, average 1.49, maximum 1.73. Ornamentation thorny-spiny-warted; yellow-brown, in KOH and water. Papilla and sterigmata remnants about the same length, 1×1 µm.

#### Distribution, habitat and seasonal appearance

*Hymenogaster intermedius* is known only from the three collection sites, mainly from mixed woodlands at different elevations. In close proximity to the collection sites *Fagus sylvatica* was identified as putative ectomycorrhizal host for both German specimens, while *Tilia platyphyllos*, *Carpinus betulus*, *Quercus petraea*, *Corylus avellana* were identified as putative hosts for the specimen from Hungary.

#### Collections examined

See File S2. **Holotype deposit:** Botanische Staatssammlung Munich; corresponding curator: Dr. D. Triebel; accession number of the holotype: N-0156227.

#### Mycobank Number

MB 518623.

#### *Hymenogaster luteus*

Vittad. 1831

Cluster: 12

Our analysis is in accordance with the species status of *H. luteus*. The varieties described for this species (*Hymenogaster luteus* var. *luteus* Vittad. 1831, *Hymenogaster luteus* f. *trigonosporus* Vacek 1948, *Hymenogaster luteus* var. *subfuscus* Soehner 1924 and *Hymenogaster luteus* var. *berkeleyanus* Corda 1854) all appear in the same cluster; moreover, they do not form monophyletic groups within the *H. luteus* clade.

#### *Hymenogaster megasporus*

Soehner 1952

Cluster: 4

*H. megasporus* is certainly a very rare taxon, but might be widely distributed across Central Europe, as it is known from exsiccates collected both in Hungary and Germany. Soehner noted that his specimens (931, 1666, 693) were always restricted to beech; in contrast, our collections were associated with *Tilia*, *Quercus* and *Carpinus*. *H. megasporus* basidiospores are not always larger than those of *H. griseus*, but at least some spores within a probe exceed 35 µm, which is not typical of *H. griseus*.

#### *Hymenogaster niveus*

Vittad. 1831 emend. Stielow et al. 2010

Including: *Hymenogaster niveus* Vittad. 1831 sensu lato  =  *Cortinomyces niveus* Bougher & Castellano 1993 =  *Protoglosum niveum* (Vittad.) T.W. May 1995, *Hymenogaster mutabilis* (Soehner) Zeller & C.W. Dodge 1934.

Clusters: 0, 3, 7, 15, 20, 21, 22

The taxa mentioned above are to be considered as synonyms until better concepts to cope with the cryptic diversity within this species complex are available. Our analysis is in disagreement with the reclassification of *Hymenogaster niveus* as *Cortinomyces niveus* [29]. The latter study placed several taxa of *Hymenogaster* into the gomphoid-phalloid fungi (Phallomycetidae), for instance to *Gautieria*. This rearrangement was not supported by molecular data [37].

#### *Hymenogaster pruinatus*

Hesse 1891

Cluster: 5

The most important characters of *H. pruinatus* are the gentle, pleasant odour, the whitish, later yellowish-brownish basidiomata and citriform, light-brown, transparent basidiospores [26]. In accordance to our results, the specimen within cluster 5 identified as *H. pruinatus* is distinguishable by the macromorphological characters outlined by [26] from *H. huthii*, originally identified by us as *H. pruinatus*. ITS sequences indicated that the collection zb1485 is unique and well differentiated from its sister taxon *H. bulliardii*. This ultra-rare taxon requires further attention and documentation in future studies.

#### *Hymenogaster rehsteineri*

Bucholtz 1901 emend. Stielow et al. 2010

Including: *Hymenogaster decorus* Tul. & C. Tul 1843.

Clusters: 8, 10, 14

According to Soehner [26], *H. rehsteineri* was one of the most abundant species in southern Germany during the early 20th century. Soehner described this taxon literally as a “jack of all trades”. However, its distribution among three distinct clusters indicates cryptic species, whose ecological preferences might be more specific. Interestingly, *Hymenogaster rehsteineri* and *H. niveus* share verrucose-thorny, papillate and partially citriform, greenish basidiospores.

#### *Hymenogaster tener*

Berk. & Broome 1844

Cluster: 11

As described above, in the case of *H. tener* Soehner's concept is in higher agreement with our results than the one of Montecchi & Sarasini [16]. Its unique spicy, rancid, floury odour and plain snow-white peridia, as well the largest spores of all species with thorny-spiny spores, differentiates *H. tener* from all other species occurring in early spring such as *H. arenarius* or *H. luteus*.

#### *Hymenogaster thwaitesii*

Berk. & Broome 1846

Cluster: 16

*H. thwaitesii*'s globose-subglobose, verrucose and dark brown spores rounded at the apex and its compact compressed gleba cells are very similar to the morphological characters of *H. griseus*. However, the globose basidiospores, the most striking and constant character of this taxon besides the yellow-brown gleba, evinces that this ultra-rare group is a distinct species and not a synonym of *H. muticus* as supposed by [16].

### North American taxa

#### *Hymenogaster gardneri*

Zeller & C.W. Dodge 1934

Probably including: *Hymenogaster subalpinus* A.H Smith 1966, *Hymenogaster parksii* Zeller & C.W. Dodge 1934, *Hymenogaster raphanodorus* M.E. Sm. & Trappe 2005

Clusters: 9, 19

Our knowledge of North American taxa is very limited. Their sequences included in our sample are originating from a large study on the evolution of sequestrate, secotioid and epigeous cortinarioid fungi [8] or were not yet used in any published datasets (AY945302, FJ789604, DQ328212). Unfortunately, a morphological comparison is impossible because descriptions and specimens have been unavailable. The synonymy of the above-mentioned species has to be treated with caution. North American species are so far only reported from the western United States (Oregon, California and Idaho). Smith [70] noted that *H. raphanodorus* and *H. rubyensis*, both restricted to dry habitats, display the same basidiospore shape, but are phylogenetically unrelated, in agreement with our findings (cluster 19 vs. cluster 20).

### Nomenclature

The electronic version of this document in itself does not represent a published work according to the International Code of Botanical Nomenclature [71], and hence the new names contained in the electronic version are not effectively published under that Code from the electronic edition alone. Therefore, a separate edition of this document was produced by a method that assures numerous identical printed copies, and those copies were simultaneously distributed (on the publication date noted on the first page of this article) for the purpose of providing a public and permanent scientific record, in accordance with Article 29 of the Code. Copies of the print-only edition of this article were distributed on the publication date to botanical or generally accessible libraries of the following institutions: Botanische Staatssammlung München, Munich, Germany; Botanical Garden Berlin-Dahlem, Berlin, Germany; Herbarium of the Martin-Luther University Halle, Halle, Germany; CABI Bioservices, Surrey, United Kingdom; CBS-KNAW, Utrecht, The Netherlands; Mycological Herbarium of the ETH Zürich, IBZ, Zurich, Switzerland). The separate print-only edition is available on request from PLoS (Public Library of Science) by sending a request to *PLoS ONE*, Public Library of Science, 1160 Battery Street, Suite 100, San Francisco, CA 94111, USA along with a check for $10 (to cover printing and postage) payable to “Public Library of Science”.

In addition, new names contained in this work have been submitted to Mycobank [24], from where they will be made available to the Global Names Index. The Mycobank LSIDs (Life Science Identifiers) can be resolved and the associated information viewed through any standard web browser. The online version of this work is archived and available from the following digital repositories: PubMedCentral and LOCKSS.

### Conclusion

The lack of a detailed study on the genus *Hymenogaster*, in the light of to the contradictory opinions of early mycologists, has caused considerable taxonomic confusion in the past. Here, we have analysed the largest morphological and molecular dataset assembled so far from taxa within family *Hymenogasteraceae*. Using the clustering optimization software OPTSIL we were able to follow a strategy involving four steps: (i) morphologically determining the material using distinct identification keys with distinct underlying species concepts; (ii) selecting the species concept from the literature which results in the highest agreement with the molecular data; (iii) localizing the remaining discrepancies between clusters and taxa; (iv) returning to morphology for interpreting and resolving these discrepancies. Based on these results, we here present a revised classification and a novel species concept for most European taxa of *Hymenogaster*. Most discrepancies between morphological and molecular data could be resolved by adopting a classification modified from Montecchi & Sarasini [16] by incorporating some of Soehner's [26] views. Only two species complexes apparently characterized by cryptic diversity [38], [72] remained, i.e. *Hymenogaster niveus* and *H. rehsteineri*. Conversely, the large clade comprising *H. griseus* sensu lato, *H. thwaitesii* and *H. megasporus* indicates that macro- and micromorphological variability does not directly coincide with a large number of cryptic species. Apparently morphology-based *Hymenogaster* taxonomy has been hindered by the presence of too constant as well as of too homoplasious characters.

It is fair to say that the genus *Hymenogaster* encompasses a number of rarely recorded taxa; some of them most likely remained as yet undescribed. Collecting and determining ultra-rare species of cortinarioid fungi remains a difficult task. Cortinarioid-hebelomatoid fungi are probably the taxonomically most difficult group of Basidiomycetes, and only a large and diverse number of well documented exsiccates collected over a long period of time is sufficient to revise systematic opinions from the past and to reclassify ultra-rare false truffles. Molecular data, especially DNA sequences, provide robust markers for mycological novelties even when the material for study is limited, such as in the case of the tiny basidiomata of *Hymenogaster*.

Clustering optimization is a valuable method for classification as it bridges the gap between traditional and modern taxonomic disciplines by directly addressing the question of how to optimally account for both genetic divergence and given taxonomic concepts [54]. While proposed as a technique of general applicability, clustering optimization might not always be able to significantly optimize the parameters. For instance, if the reference data are biologically largely meaningless (as in the case of a very poor classification) or if the gene used is unresolved at the taxonomic level of interest, the agreement between reference and clustering partitions might be hardly be modifiable by changing the clustering parameters. However, such cases can easily be recognized by globally optimal MRI values significantly below 1.0 [54]. Another potential problem regarding the interpretability of the outcome of clustering optimization is that the obtained clusters might be non-monophyletic with significant support. Nevertheless, the results presented here and elsewhere [54], [59] indicate that such problems might by negligible in practice.

There are important differences in perspective between clustering optimization and algorithms such as the general mixed Yule-coalescent model (GMYK) [73], [74], ecotype simulation [75] and the biological species concept for Bacteria [76]. The latter are based on population theory, allowing one to not only delineate species but also to test hypotheses about the modes of speciation. Depending on the underlying assumptions, the speciation models might or might not be applicable to the organisms under study; for instance, the limitations of GMYK have been highlighted in [75] (see also the problems of applying GMYK to ITS data described in [77]). Moreover, the models might contradict each other as in the case of the conflict between the ecotype model [75] and the biological species concept for Bacteria [76]. For this reason, it has been called into question whether a robust species concept can be obtained using theory-based algorithms [78]. In contrast, clustering optimization only aims at maximizing conservatism (in the sense of minimal changes of the previous classification) and consistency (in the sense of minimal deviations in character divergence between the resulting clusters) [54]. This flexibility also allows its use in optimizing distance functions for molecular taxonomy [59]. Whether species are ‘real’ or not is not of interest for clustering optimization, much like in [78], but we maintain that biologists can hardly do without the species category in practice because of its importance as a means for managing the deposition of and the access to biological material [79]. It is likely that such a pragmatic view on the species category will remain dominant in the future; for instance, current Prokaryote taxonomy appears to be largely unaffected by the proposed ‘paradigm shift’ [75] in species delineation. In contrast, species delimitation in Archaea and Bacteria is likely to remain to be ultimately based on the 70% similarity threshold in DNA-DNA reassociation, which was established to mimic phenotype-based species concepts in Enterobacteria [80] and has recently be successfully emulated itself by the comparison of genome sequences [81], [82]. Adapting a more suitable method for classification to a previous taxonomy is precisely the logic behind clustering optimization [54].

## Supporting Information

File S1 **Taxonomic descriptions based on the 142 dried and fresh basidiomata collected by the authors (** **Table 1** **) and illustrated identification key for the Central European** ***Hymenogaster*** **species based on the revised classification (PDF format).**
(PDF)

File S2 **CSV file with list of specimens used for sequence and comparative morphological analysis, identification results obtained with the three distinct keys and corresponding literature reference for each specimen, taxonomic affiliation according to the revised classification, and Genbank accession numbers.**
(CSV)

File S3 **Compressed archive including the inferred alignment in FASTA format and the trees in Newick format.**
(TAR)

File S4 **Measurements of basidiospore length and width in CSV format.**
(CSV)
