# Supplementary material for: Species Delimitation in Taxonomically Difficult Fungi: The Case of Hymenogaster
Source: PLoS One. 2011 Jan 25;6(1):e15614. doi: 10.1371/journal.pone.0015614 (PMC3027480; doi:10.1371/journal.pone.0015614)

Identification key to the Central European species of the genus *Hymenogaster*

Authors:

Stielow, B., Bratek, Z., Orczán, K.A., Hensel, G., Hoffmann P., Klenk, H.-P.,

Göker, M.

Identification key to *Hymenogaster*

Content

1. **Background information**
2. **Addresses of the authors**
3. **Identification key to the European *Hymenogaster* species**
4. **List of figures**
5. **Species descriptions**

Identification key to *Hymenogaster*

1. Background information

This key is published as supplementary material to the paper ”Species delimitation in taxonomically difficult fungi: The case of *Hymenogaster*“. It is useful as a stand-alone document for the identification of the many taxonomically difficult *Hymenogaster* species occurring in Central Europe. The key is specifically excluding all Mediterranean species. A broad overview including more than 100 pictures and a detailed key is provided, necessary for the identification of every considered species based on macro- and micromorphological characters. The specimens IDs for all images are listed in chapter four. The complete collection data are available in Table 1 of the full paper.

This key is published under the terms of the Creative Commons Attributions Licence (http://creativecommons.org/licenses/by/3.0/).

Identification key to *Hymenogaster*

2. Addresses of the authors

Stielow, B.[1], Bratek, Z.[2], Orczán, K.A.[2], Hensel, G.[3], Hoffmann P.[1], Klenk, H.-P.[1], Göker, M.[1]

[1] DSMZ – German Collection of Microorganisms and Cell Cultures GmbH, Inhoffenstraße 7 B, 38124 Braunschweig, Germany

[2] Department of Plant Physiology and Molecular Plant Biology, ELTE University, Pázmány Péter sétány 1/C, 1518 Budapest, Hungary

[3] Fungarium Gunnar Hensel, Alte Lauchstädter Straße 22, 06217 Merseburg, Germany

Identification key to *Hymenogaster*

3. Identification key to the Central European Hymenogaster species

(1) a. Spores smooth, yellow, whitish-yellowish, yellowish-brownish, transparent; perisporium absent………..…………….....…………….…2

(1) b. Spores verrucose, wrinkled, warted, thorny, dark coloured, blackish-brownish, reddish-brownish, greenish-yellowish; perisporium always present…...……………...…...…...4

(2) a. Peridia whitish-grey-yellowish to pale grey-white; gleba dark black to light brown-black with yellow coloured trama; odour of mature basidiomata sweet fruity, sour fruity, rancid…..…........…*H. citrinus* (p. 9)

(2) b. Peridia without intensive yellow tones (compare to *H. citrinus;* alternatively to *H. thwaitesii*); gleba predominantly reddish, brownish (-yellowish), yellowish, blackish……………………………..……….…....3

(3) a. Peridia white, turning brown-blackish with whitish tones; gleba reddish; odour unpleasant……....……………………..*H. bulliardii* (p. 15)

(3) b. Peridia pale to brilliant white; gleba yellowish; odour pleasant, reminding of fruits…………...…*H. luteus* (p. 18)

(4) a. Peridia bright yellow to dark yellow-black to pale grey-white(-yellow); gleba dark black to light brown-black with yellow coloured trama; globose-verrucose basidiospores not predominant; odour of mature basidiomata sweet fruity, sour fruity, rancid…..……….....*H. citrinus* (p. 15)

(4) b. Peridia with various colourations; basidiospores of various shapes, with verrucose or spiny-thorny ornamentation (note: the peridia may

have a yellow-brown colouration, but never as intense as those of *H. citrinus*, see images starting on p. 15)……...........…………….............5

(5) a. Average spore length less than 18µm…………………...…………6

(5) b. Average spore length more than 18 µm……………….……………9

(6) a. Peridia brilliant white; basidiospores with pronounced spiny ornamentation; basidiomata with strong, pleasant floury odour, unpleasant when mature; basidiomata found in early spring time (January to March)………..…*H. tener* (p. 24)

(6) b. Peridia brilliant white to pale grey-white; basidiomata not found in early spring. Alternatively, basidiospores without pronounced spiny ornamentation, peridia pale grey-white to brown-carmine...….......7

(7) a. Peridia brilliant white; gleba grey-white to grey-brown to dark reddish; odour of basidiomata not unpleasant rancid/floury/cucumber-like; basidiomata not found in early spring, basidiospores ovoid-citriform, yellow, yellow-light brown……………...…..……...……*H. niveus* (p. 26)

(7) b. Peridia pale grey-white to brown-carmine or white to creamy-ochraceous; odour of basidiomata unpleasant, rancid garlic-like to pleasant floury/cucumber-like……..…………………………………8

(8) a. Peridia grey-white to brown-grey-carmine; odour very unpleasant, rancid, garlic-like, basidiospores broad ellipsoid, to citriform or spindle-like shape…………………...…………...…………*H. arenarius* (p. 33)

Identification key to *Hymenogaster*

(8) b. Peridia whitish to creamy-ochraceous (similar to *H. rehsteineri*); odour of basidiomata pleasant floury/cucumber-like; basidiospores similar to those of *H. niveus*...………………………*H. intermedius* (p. 38)

(9) a. Peridia yellow-brown; basidiospores (10-)21(-29) x (7-)13(-24), predominant shape globose-subglobose to ovoid (Q=1.6), but mucronate-fusoid spores always present……….......*H. thwaitesii* (p. 41)

(9) b. Peridia yellow brown, grey, brown-white, pale mixed tones; basidiospores 10-40 x 5-21 µm, globose to ovoid shape not predominant, rather mucronate-papillate-fusoid-lanceolate, ornamentation verrucose, warted or wrinkled……………..……………10

(10)a. Peridia with lilac-purple tones; basidiospores partially very large (>35 µm), but small spores present in most specimens.. ……………………........……………..….*H. megasporus* (p. 43)

(10)b. Other peridia colours predominant; spores not unusually large (<35 µm)……………………………………........…...…………….11

(11)a. Peridia of juvenile and mature basidiomes brilliant white (reminding of *H. niveus*); basidiospores ellipsoid, broadly fusoid, strongly wrinkled, folded inwards, dark to light rusty-brown………….. ……………………………………………..*H. huthii* (p. 46)

(11)b. Peridia not brilliant white, predominantly pale brownish-greyish-yellowish with whitish tones............………………………....…...12

(12)a. Macro- and micromorphological characters of basidiomata similar to *H. huthii* (p. 46); peridia plain white, at maturity with dispersed yellowish-brownish spots; basidiospores citriform, brown-verrucose,

transparent; odour of basidiomata pleasant………………….....*H. pruinatus*

Note: *H. pruinatus* is an ultra-rare taxon that could be falsely determined as *H. griseus*. However, *H. griseus* can probably only distinguished from *H. pruinatus* by DNA sequence analysis. *H. griseus* and *H. rehsteineri* are more common taxa and more likely to be found. *H. pruinatus*, as the only taxon, is not present in part 5, species descriptions, as no images are available of this species.

(12)b. Basidiospores strongly verrucose; basidiomata extremely variable in colouration from light to dark (yellow-)brown to whitish-brown to entirely grey; odour of basidiomata unpleasant to pleasant...... ……………………………………………………………….13

(13)a. Spores predominantly asymmetrical, fusoid-mucronate, partially ribbed, strongly verrucose, but not spiny or warted; basidiomata extremely variable in colouration…......…..*H. griseus* (p. 49)

(13)b. Spores predominantly symmetrical, elongated-lanceolate, ornamentation verrucose-warted, slightly spiny; gleba light to dark brown; basidiomata grey-ochraceus (similar to *H. intermedius*): ……………………………....................*H. rehsteineri* (p. 56)

Notes: Always compare with macro- and micromorphological images of both taxa as *H. rehsteineri* and *H. griseus* are the two most variable *Hymenogaster* taxa.

Identification key to *Hymenogaster*

List of figures

Figure	Taxon	Character	Specimen ID
1	*Hymenogaster citrinus*	Basidiomata	it8_1
2	*Hymenogaster citrinus*	Basidiomata	it6_5_1
3	*Hymenogaster citrinus* (*olivaceus*)	Basidiomata	not in dataset
4	*Hymenogaster citrinus* (*bucholtzii*)	Basidiomata	it8_3_1
5	*Hymenogaster citrinus* (*olivaceus*)	Basidiomata	zb3857
6	*Hymenogaster citrinus* (*olivaceus*)	Basidiomata	zb2300
7	*Hymenogaster citrinus* (sp.)	Basidiomata	it7_2
8	*Hymenogaster citrinus*	Basidiospores	it6_5_1
9	*Hymenogaster citrinus*	Basidiospores	it8_1
10	*Hymenogaster citrinus*	Basidiospores	it8_1
11	*Hymenogaster citrinus*	Basidiospores	it4_2
12	*Hymenogaster citrinus*	Basidiospores	it15_1_1
13	*Hymenogaster citrinus*	Basidiospores	it15_1_1
14	*Hymenogaster bulliardii*	Basidiomata	it20_4_1
15	*Hymenogaster bulliardii*	Basidiomata	it20_4
16	*Hymenogaster bulliardii*	Basidiomata	zb3722
17	*Hymenogaster bulliardii*	Basidiospores	it20_4_1
18	*Hymenogaster bulliardii*	Basidiospores	it20_4_1
19	*Hymenogaster bulliardii*	Basidiospores	it5_21
20	*Hymenogaster bulliardii*	Basidiospores	it5_21
21	*Hymenogaster luteus*	Basidiomata	it6_22
22	*Hymenogaster luteus* (var. *luteus*)	Basidiomata	not in dataset
23	*Hymenogaster luteus* (var. *subfuscus*)	Basidiomata	it5_21_6
24	*Hymenogaster bulliardii*	Basidiomata	it5_21
25	*Hymenogaster luteus* (var. *pilosiusculus*)	Basidiomata	it11_3_1
26	*Hymenogaster luteus*	Basidiomata	zb2603
27	*Hymenogaster luteus*	Basidiospores	it6_22
28	*Hymenogaster luteus*	Basidiospores	it11_2
29	*Hymenogaster luteus*	Basidiospores	it6_22
30	*Hymenogaster luteus*	Basidiospores	it11_2
31	*Hymenogaster luteus*	Basidiospores	it5_21_6
32	*Hymenogaster tener*	Basidiomata	it15_2
33	*Hymenogaster tener*	Basidiospores	it15_2
34	*Hymenogaster niveus* (*mutabilis*)	Basidiomata	it9_5_1
35	*Hymenogaster niveus*	Basidiomata	it17_3
36	*Hymenogaster niveus*	Basidiospores	it10_5_1
37	*Hymenogaster niveus*	Basidiospores	it10_5_1
38	*Hymenogaster niveus* (*mutabilis*)	Basidiospores	it9_5_1
39	*Hymenogaster niveus* (*mutabilis*)	Basidiospores	it9_5_1
40	*Hymenogaster niveus*	Basidiospores	it17_3
41	*Hymenogaster niveus*	Basidiospores	it17_3
42	*Hymenogaster niveus*	Basidiospores	it18_3
43	*Hymenogaster rehsteineri*	Basidiospores	it2_4_1
44	*Hymenogaster arenarius*	Basidiomata	it10_26
45	*Hymenogaster arenarius*	Basidiomata	it5_2
46	*Hymenogaster arenarius*	Basidiomata	not in dataset
47	*Hymenogaster arenarius*	Basidiomata	not in dataset
48	*Hymenogaster arenarius*	Basidiospores	it10_26
49	*Hymenogaster arenarius*	Basidiospores	it10_26
50	*Hymenogaster arenarius*	Basidiospores	it5_2

51	*Hymenogaster arenarius*	Basidiospores	it5_2
52	*Hymenogaster arenarius*	Basidiospores	it5_2
53	*Hymenogaster arenarius*	Basidiospores	it5_2
54	*Hymenogaster arenarius* (*pusillus*)	Basidiomata	it1_2
55	*Hymenogaster arenarius* (*pusillus*)	Basidiomata	it1_2
56	*Hymenogaster arenarius* (*pusillus*)	Basidiospores	it1_2
57	*Hymenogaster arenarius* (*pusillus*)	Basidiospores	it1_2
58	*Hymenogaster arenarius* (*pusillus*)	Basidiospores	it1_2
59	*Hymenogaster arenarius*	Basidiospores	it5_2
60	*Hymenogaster arenarius*	Basidiospores	it10_26_2
61	*Hymenogaster intermedius*, holotype	Basidiomata	it16_2
62	*Hymenogaster intermedius*, holotype	Basidiospores	it16_2
63	*Hymenogaster intermedius*, holotype	Basidiospores	it16_2
64	*Hymenogaster intermedius*, holotype	Basidiospores	it16_2
65	*Hymenogaster thwaitesii*	Basidiomata	it10_2
66	*Hymenogaster thwaitesii*	Basidiospores	it10_2
67	*Hymenogaster thwaitesii*	Basidiospores	it10_2
68	*Hymenogaster thwaitesii*	Basidiospores	it3_2
69	*Hymenogaster thwaitesii*	Basidiospores	it3_2
70	*Hymenogaster megasporus*	Basidiomata	it12_1
71	*Hymenogaster megasporus*	Basidiospores	it12_1
72	*Hymenogaster megasporus*	Basidiospores	it8_5_1
73	*Hymenogaster megasporus*	Basidiospores	it12_1
74	*Hymenogaster huthii*, holotype	Basidiomata	it12_3_1
75	*Hymenogaster huthii*, holotype	Basidiospores	it12_3_1
76	*Hymenogaster huthii*, holotype	Cylindrical basidia and basidiospores	it12_3_1
77	*Hymenogaster huthii*, holotype	Abnormal roundish three-spored basidia	it12_3_1
78	*Hymenogaster griseus* (*hessei*)	Basidiomata	it3_19
79	*Hymenogaster griseus* (*hessei*)	Basidiomata	it2_18
80	*Hymenogaster griseus* (*populetorum*)	Basidiomata	it16_1
81	*Hymenogaster griseus* (*populetorum*)	Basidiomata	it16_1_1
82	*Hymenogaster griseus* (*griseus*)	Basidiomata	it1_17
83	*Hymenogaster griseus* (*vulgaris*)	Basidiomata	it13_2
84	*Hymenogaster griseus* (*vulgaris*)	Basidiomata	not in dataset
85	*Hymenogaster griseus* (*vulgaris*)	Basidiomata	not in dataset
86	*Hymenogaster griseus* (*vulgaris*)	Basidiomata	not in dataset
87	*Hymenogaster griseus* (*vulgaris*)	Basidiomata	not in dataset
88	*Hymenogaster griseus*	Basidiospores	it13_2
89	*Hymenogaster griseus*	Basidiospores	it14_2
90	*Hymenogaster griseus*	Basidiospores	it14_2
91	*Hymenogaster griseus*	Basidiospores	it3_5_1
92	*Hymenogaster griseus*	Basidiospores	it1_5_1
93	*Hymenogaster griseus*	Basidiospores	it4_5_1
94	*Hymenogaster griseus*	Basidiospores	it16_1_1
95	*Hymenogaster griseus*	Basidiospores	it1_17
96	*Hymenogaster rehsteineri*	Basidiomata	it2_4_1
97	*Hymenogaster rehsteineri*	Basidiospores	it2_4_1
98	*Hymenogaster rehsteineri*	Basidiomata	it3_4_1
99	*Hymenogaster rehsteineri*	Basidiospores	it3_4_1
100	*Hymenogaster rehsteineri*	Basidiospores	it3_4_1

Identification key to *Hymenogaster*

5. Species descriptions

Hymenogaster citrinus Vittad. 1831 emend. Stielow et al. 2010

Spores: (10-) 26 (-50) x (7-) 13 (-23) µm in diameter; broad to elongated, citriform, fusoid.

Ornamentation: Verrucose to wrinkled (or entirely smooth; sometimes only one type is present), many different graduations and intermediate forms.

Basidiomata: Regularly roundish, oval, silky-shiny appearance, ca. 1-7 cm in diameter.

Peridia: Surface smooth, wrinkled, colouration whitish-brownish-greyish-yellowish when juvenile, turning dark black-brown, sometimes with predominant white-yellow-orange tones at maturity.

Gleba: Consistency compact, enduring, not compressed, strongly chambered, labyrinthoid; whitish-ochraceous-greyish when juvenile, becoming very dark coloured, with blackish-brownish but alternatively also with bride yellowish-orange tones at maturity; trama plates whitish-yellowish-lemon (an unstable character which is not always present).

Odour: Sweet to rancid, reminding of fruits and nuts.

Ecology: The species can be found in various soils and associated with a wide range of deciduous trees; when associated to coniferous trees, than only with *Pinaceae*.

Images:

Basidiomata: No. 1-7. Basidiospores: No. 8-13. Montecchi & Sarasini 2000, p. 468, 490.

Identification key to *Hymenogaster*

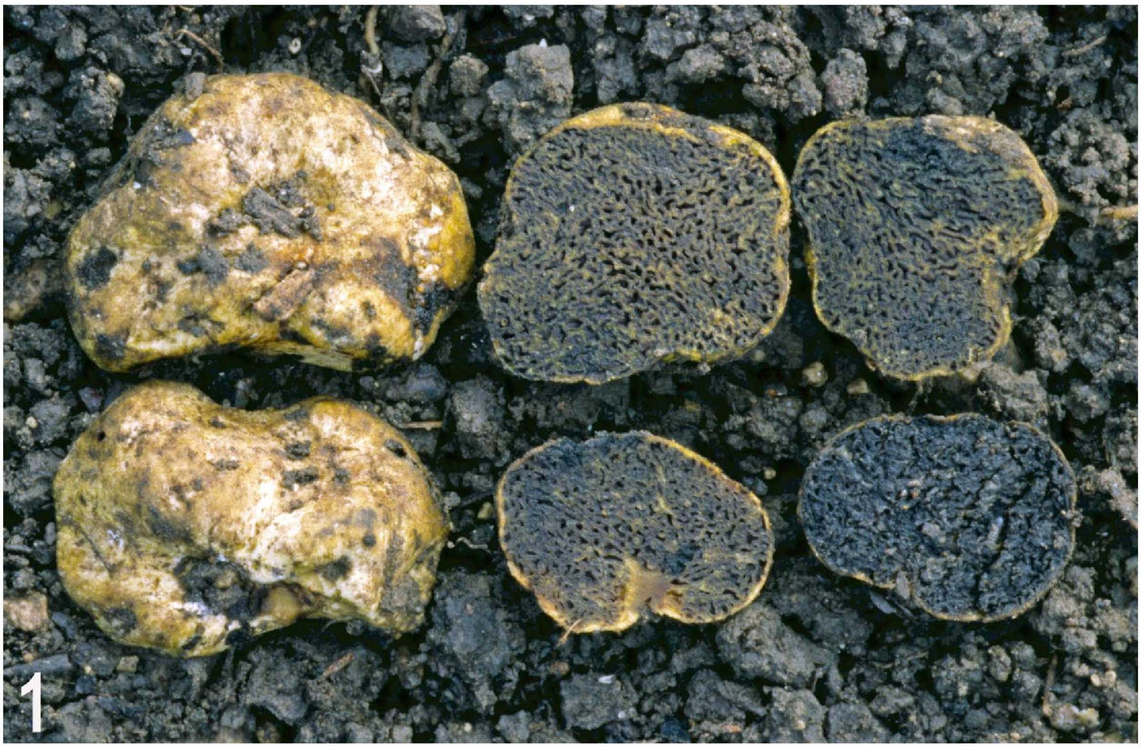

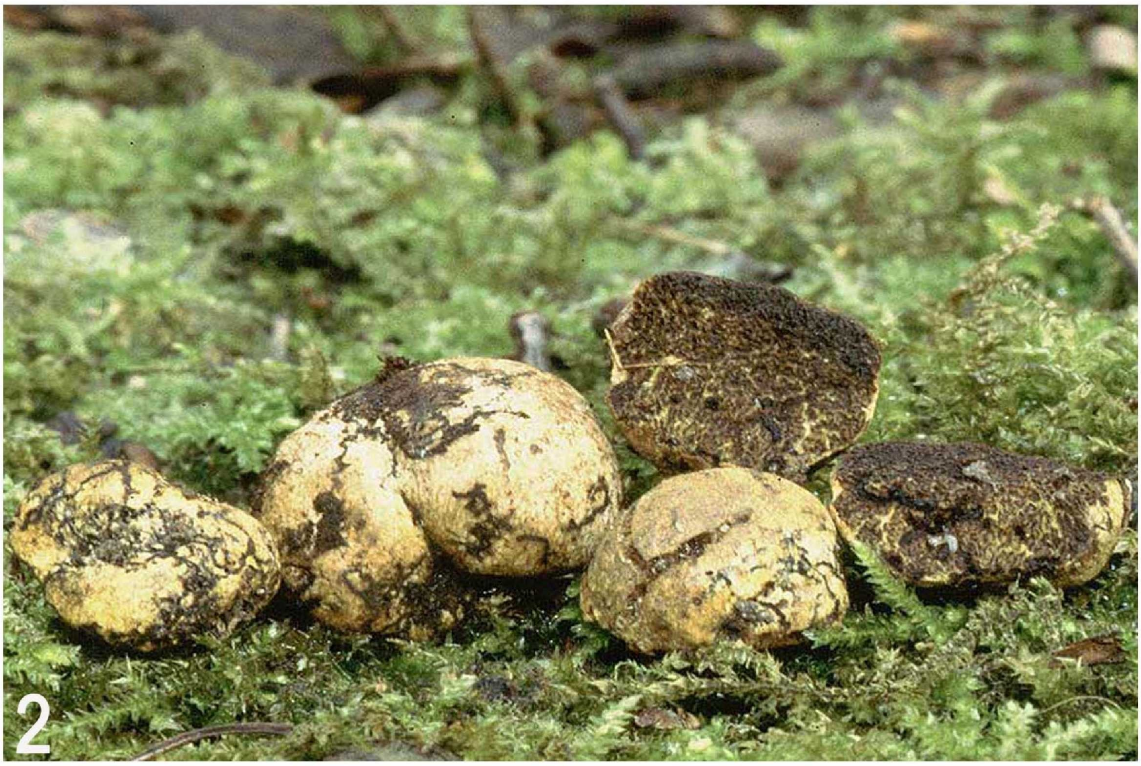

Identification key to *Hymenogaster*

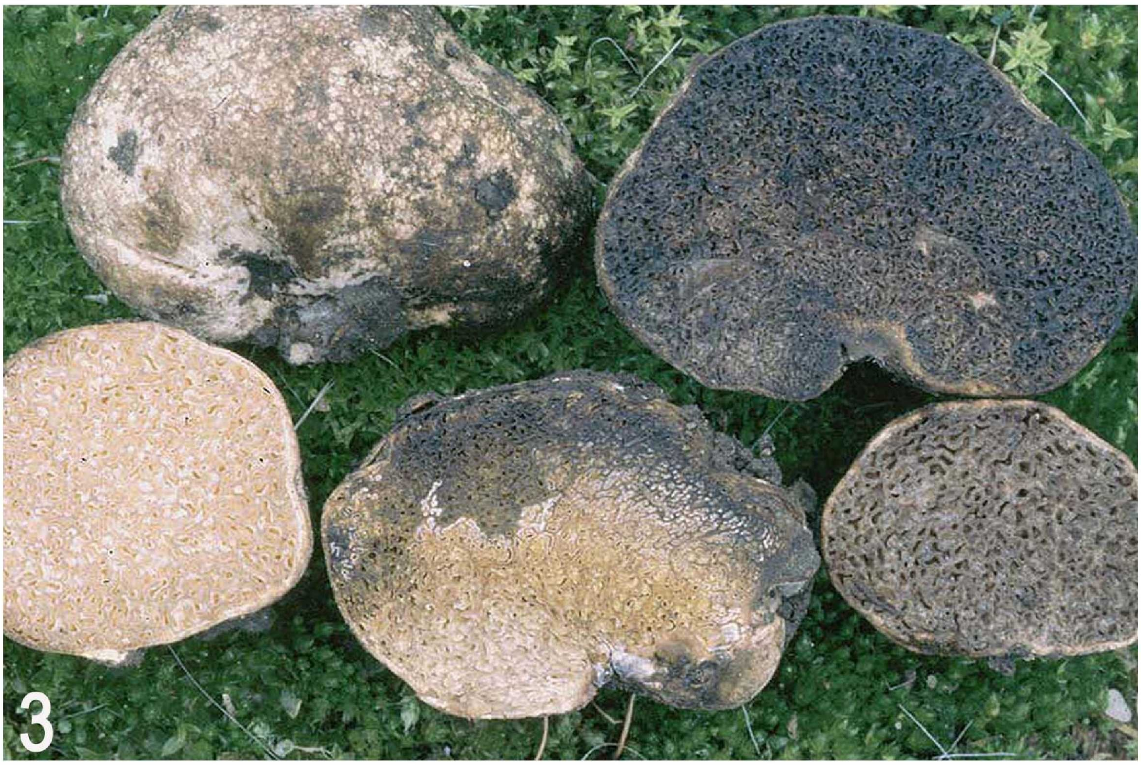

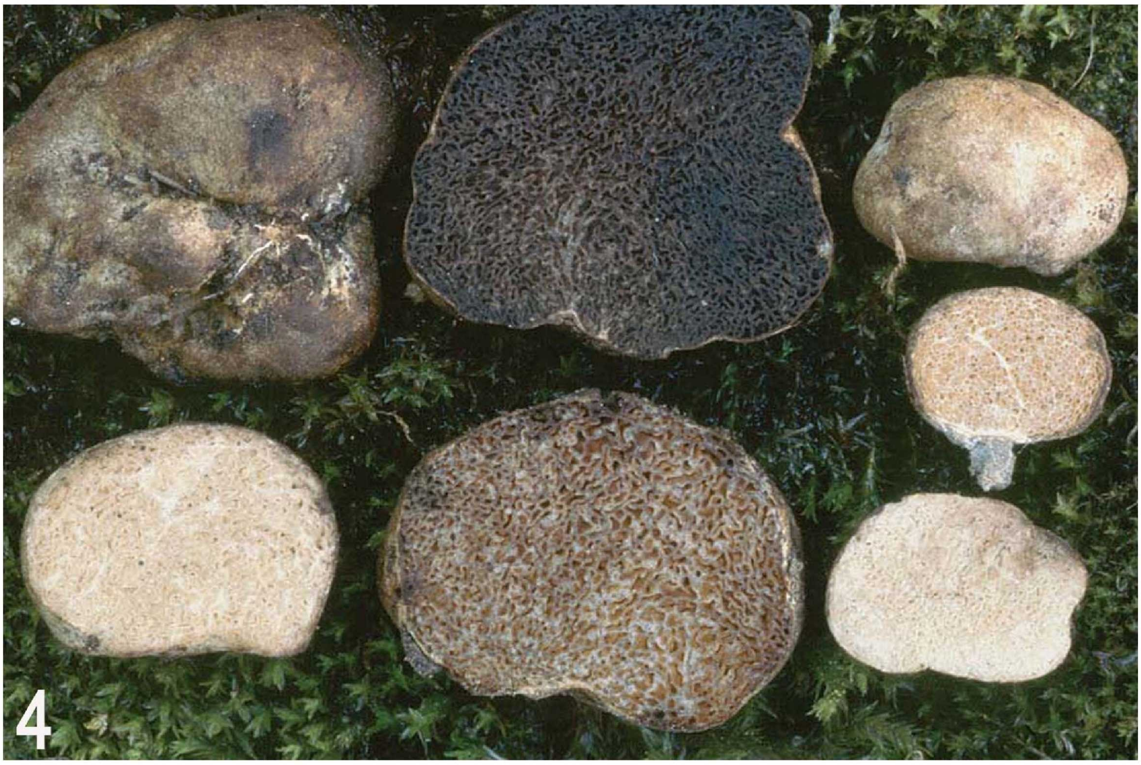

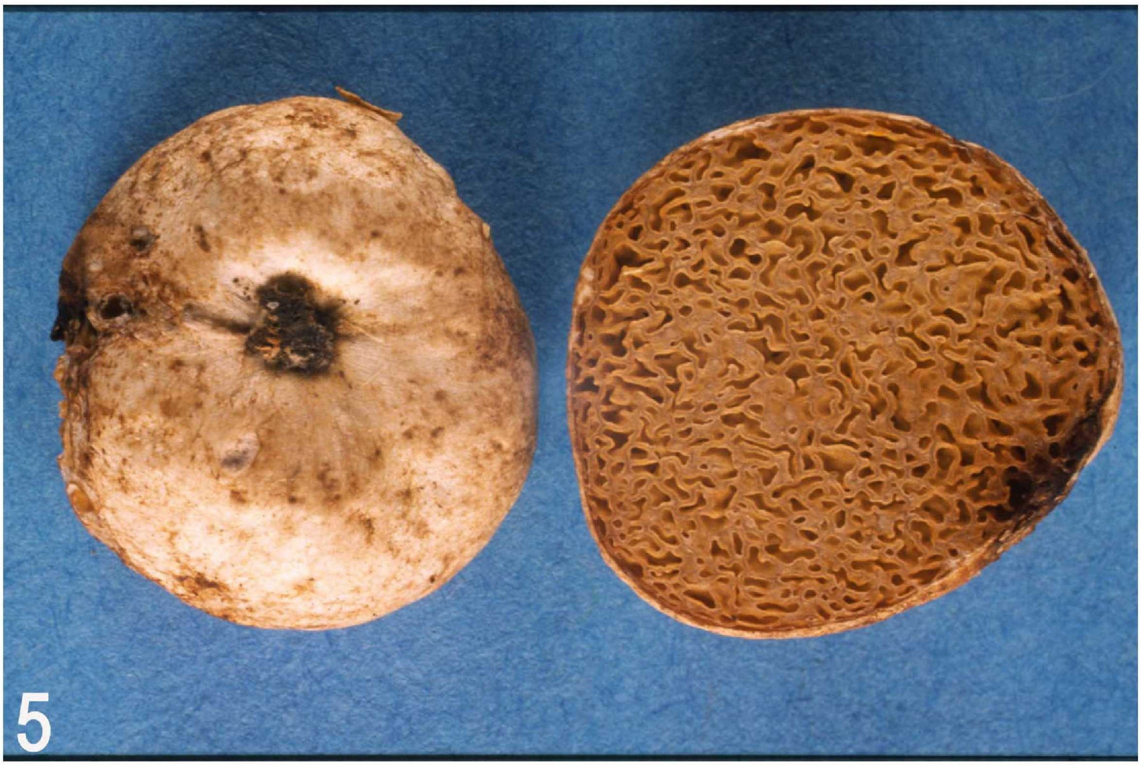
5

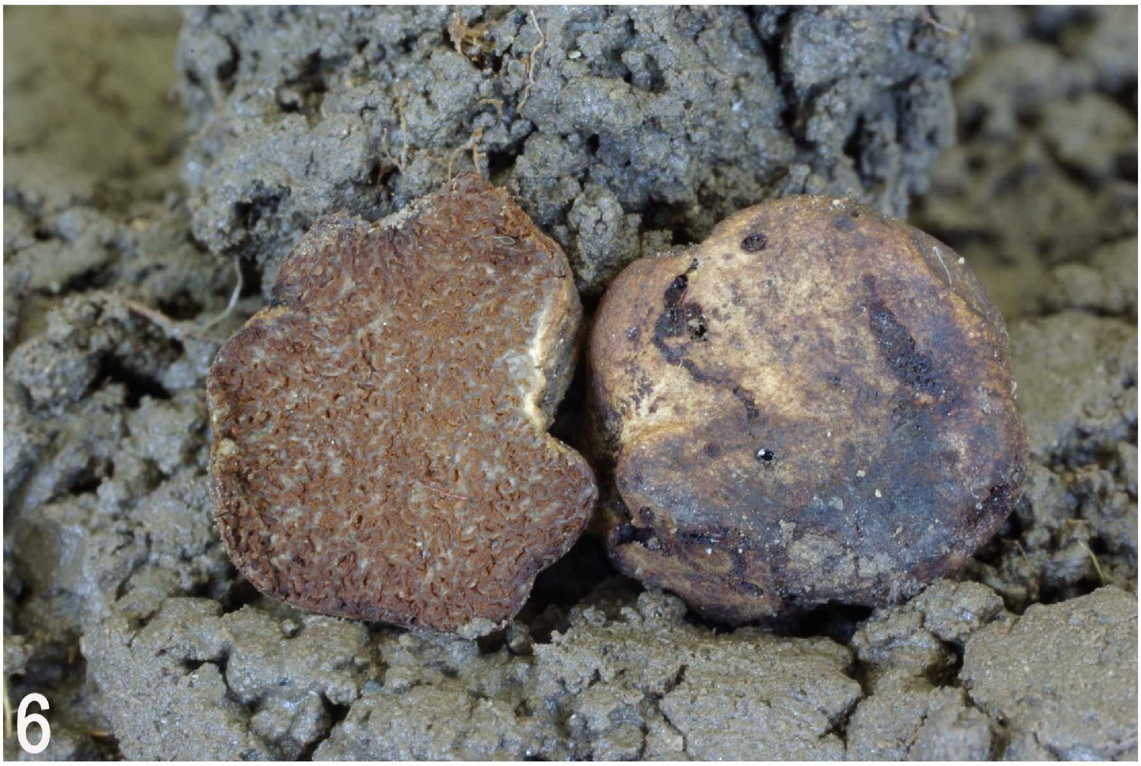
6

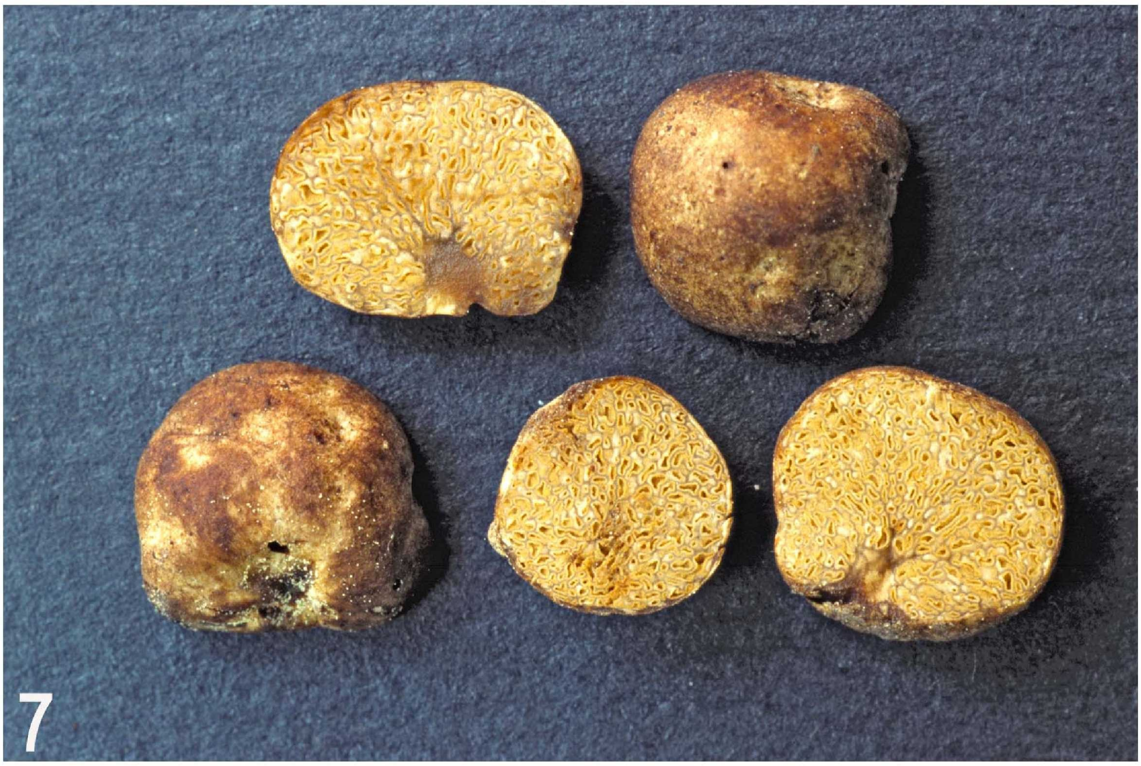
7

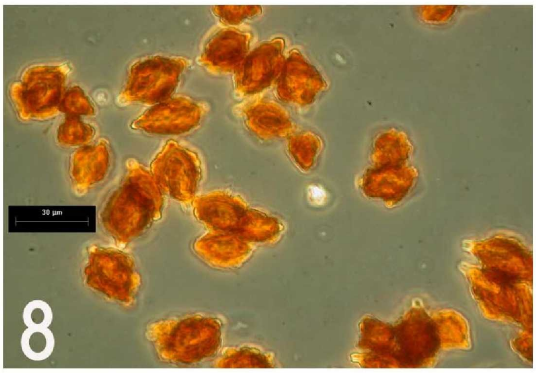
8

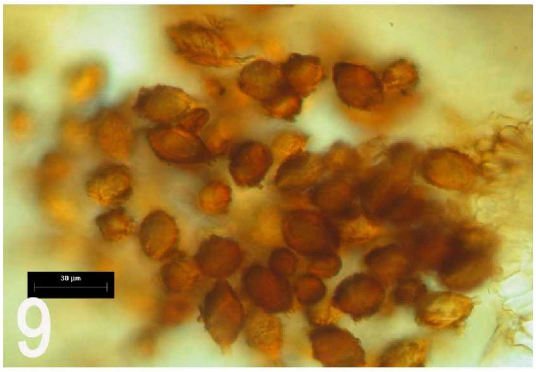
9

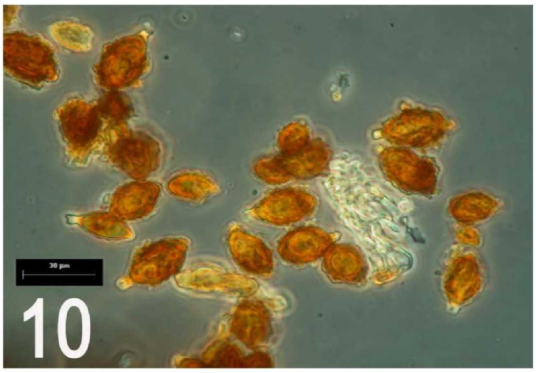
10

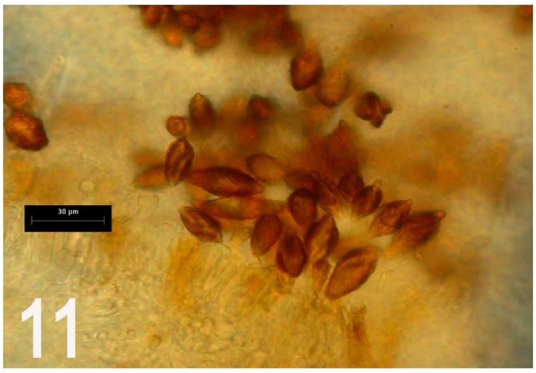
11

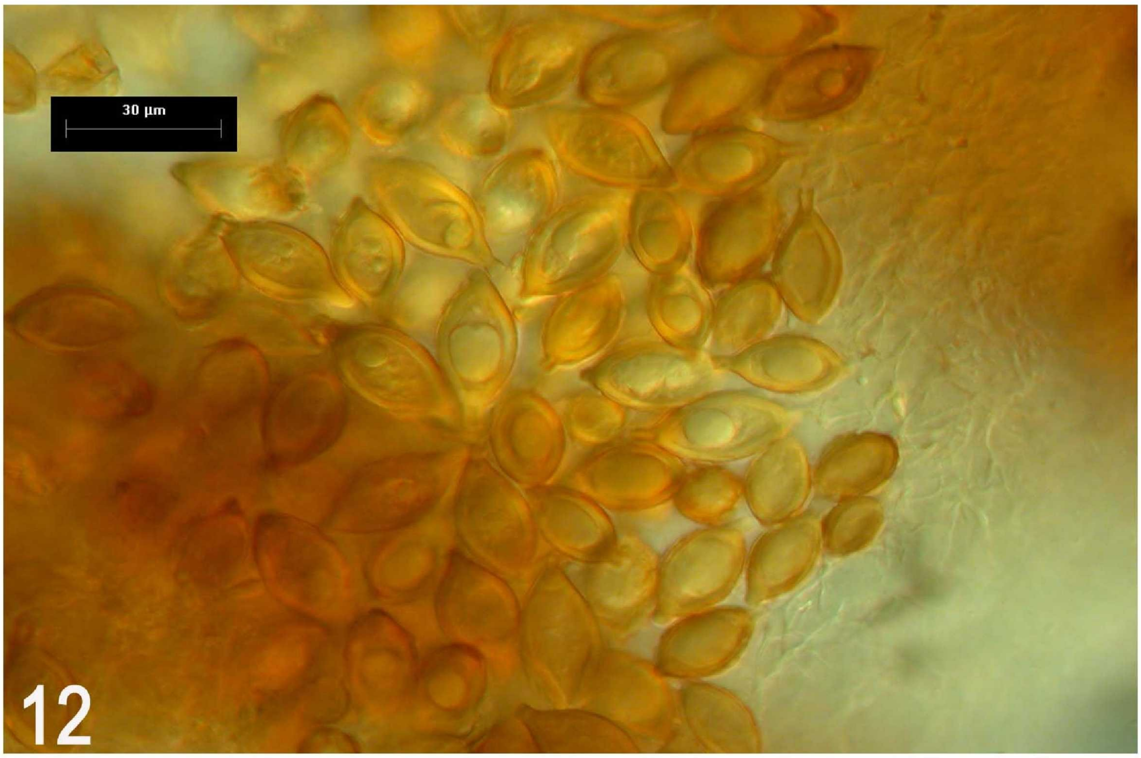
30 µm
12

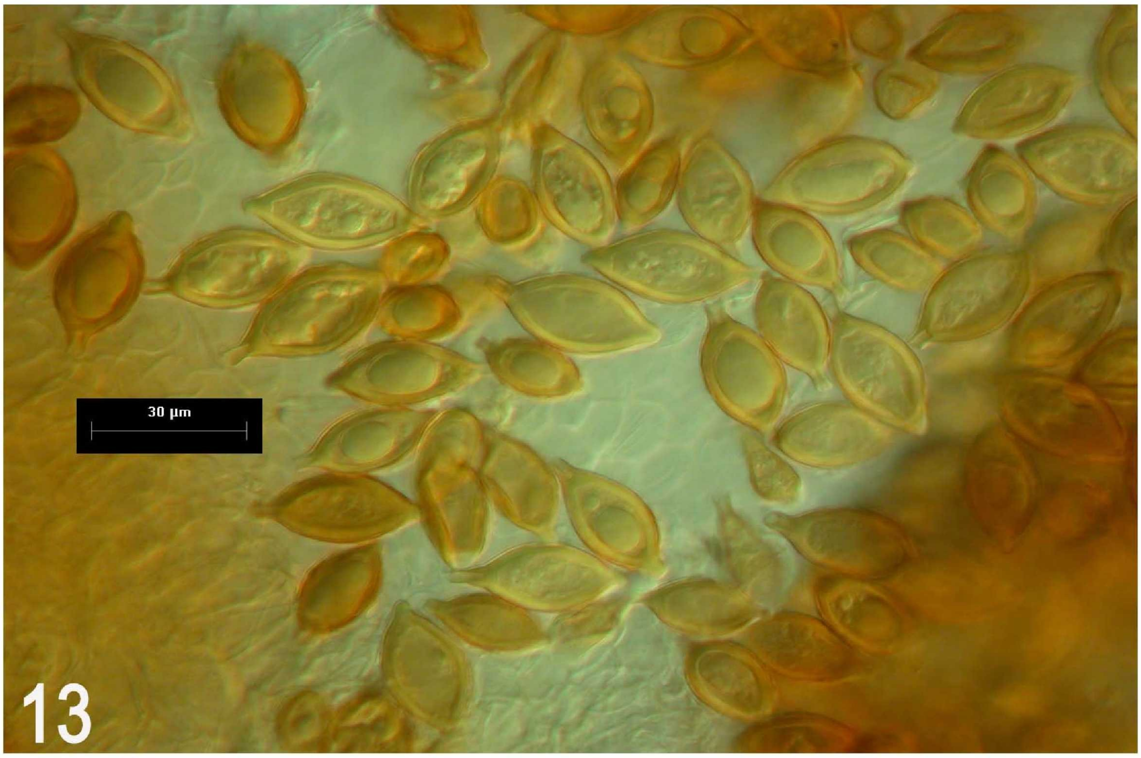
30 µm
13

Identification key to *Hymenogaster*

Hymenogaster bulliardii Vittad. 1831

Spores: (12-) 20 (-31) x (7-) 11 (-19) µm in diameter; smooth surface, not ornamented; transparent; fusoid, elongated to ovoid. Spores with distinct abnormalities (secondary spores) always present and a distinct species character for *H. bulliardii*, often triangle- or cup-shaped.

Basidiomata: Roundish, knotted, partially wrinkled, ca. 1.5-4 cm in diameter.

Peridia: Yellowish-brownish, ochraceous coloured, smooth, thin, slightly furrowed.

Gleba: Consistency very compact and compressed; composed of small roundish cells; very constantly brownish-reddish(-black) coloured at maturity, yellowish-(whitish) coloured when juvenile, shimmed by reddish tones (Images 14-15).

Odour: Very unpleasant, reminding of putrescent vegetables.

Ecology: The species is always associated with deciduous trees in habitats on calcareous soils (can be confounded with *H. luteus* var. *berkeleyanus*).

Images:

Basidiomata: No. 14-16. Basidiospores: No. 17-20 (secondary spores: No. 17-19 or Montecchi & Sarasini 2000, p. 463)

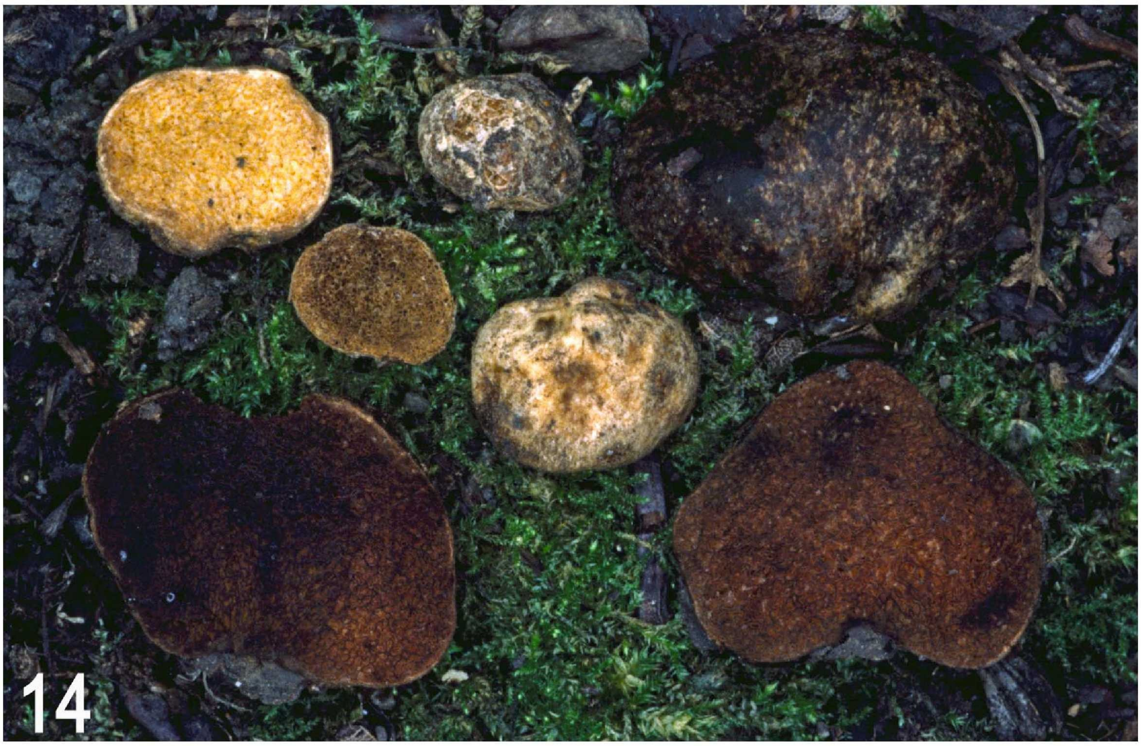
14

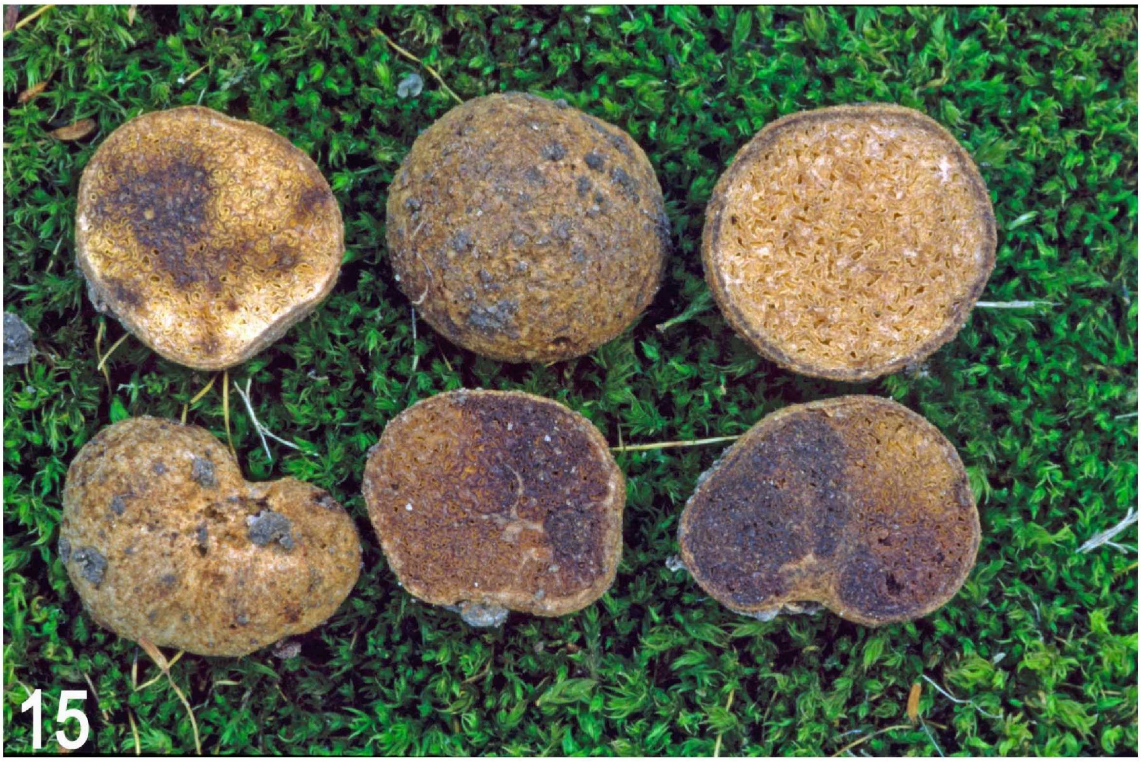
15

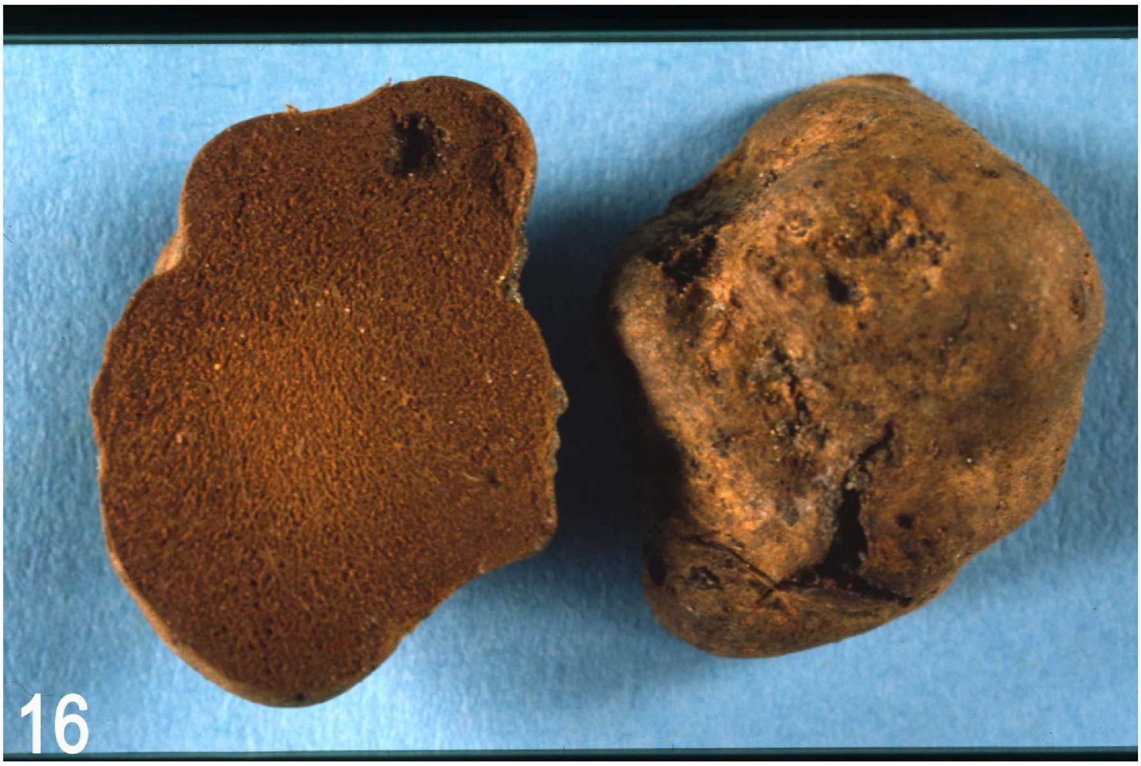
16

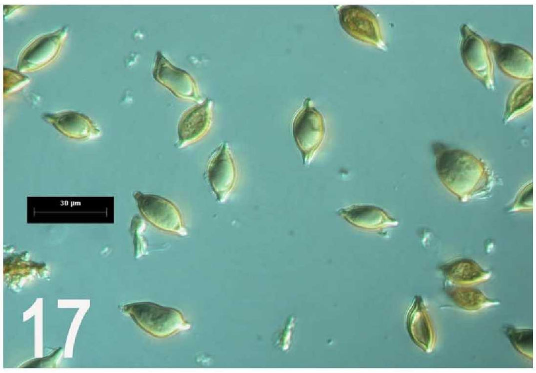
17

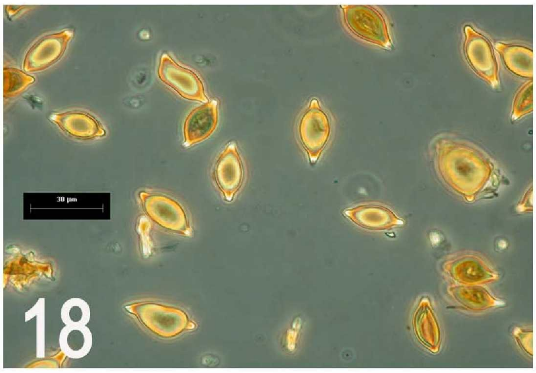
18

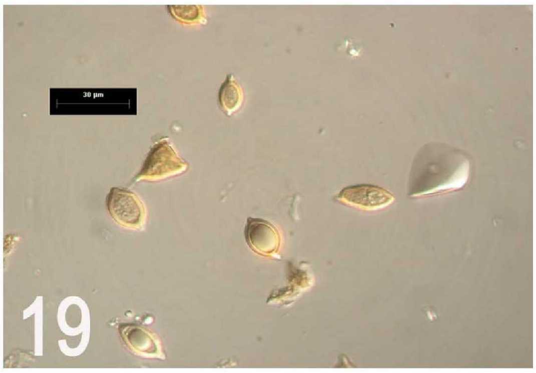
19

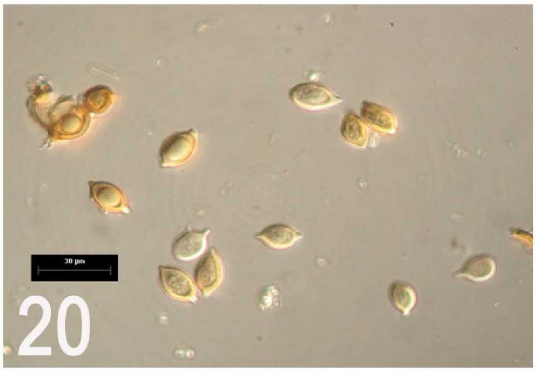
20

Identification key to *Hymenogaster*

Hymenogaster luteus Vittad. 1831

Spores: (14-) 20 (-36) x (6-) 9 (-14) µm in diameter; fusoid, elongated to ovoid; spores with distinct abnormalities rarely present.

Ornamentation: None, spore surface smooth. (The opinion of Montecchi & Sarasini regarding the brownish ornamentation of spores can not be confirmed; see Montecchi & Sarasini 2000, p. 479.)

Basidiomata: Globose or subglobose, ca. 0.2 - 2 cm in diameter.

Peridia: Pale white to grey-white, fluffy-smooth, very thin. Whitish-greyish at maturity but entirely whitish when juvenile.

Gleba: Consistency very compact and compressed; composed of small roundish cells; very constant characters are the bright predominant yellow to pale yellowish-ochre-brownish colouration at maturity. *H. luteus* var. subfuscus contains more distinct reddish-ochre tones according to Soehner (1962) (see image 23); differentiation from *H. luteus* var. *luteus* is ambiguous. The varieties of *H. luteus* can easily be confounded with *H. bulliardii* (compare image 23, most likely *H. luteus* var. *subfuscus*, with image 24, representing *H. bulliardii*).

Odour: Pleasant, reminding of fruits and flowers.

Ecology: A species that is only found on calcareous soils and under deciduous trees, from late October to early spring time, a typical winter species.

Images: Basidiomata: No. 21-26. Basidiospores: No. 27-31.

Identification key to *Hymenogaster*

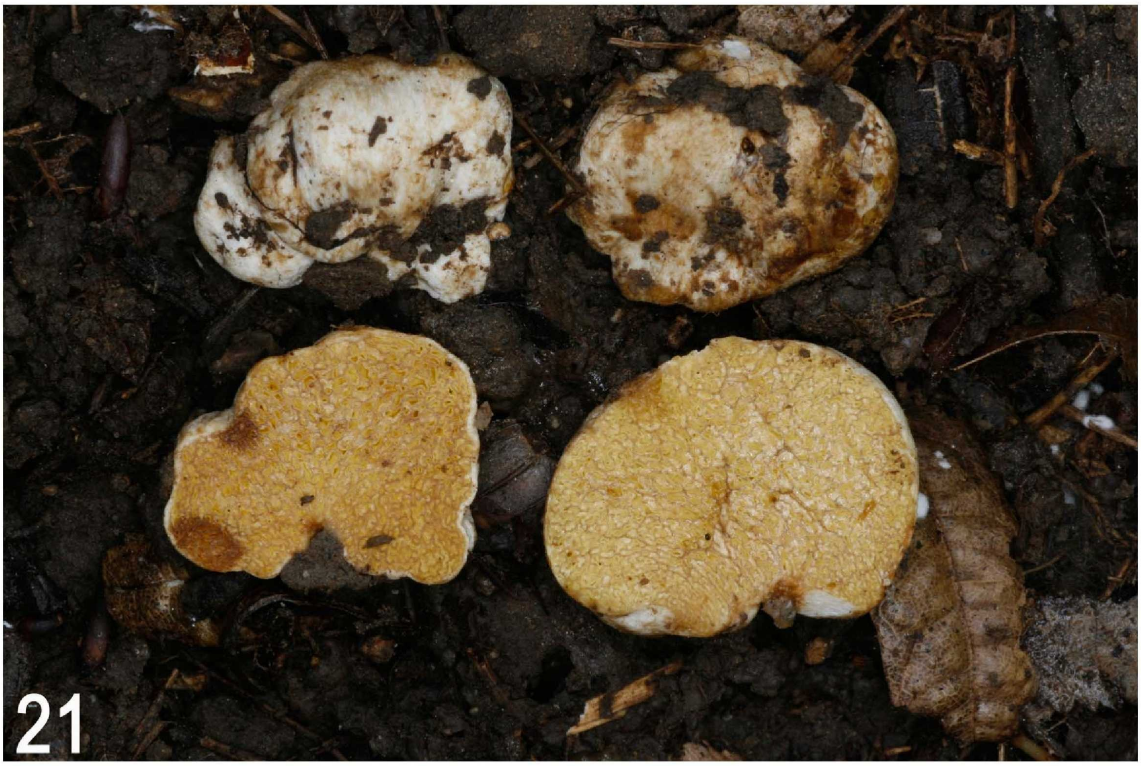

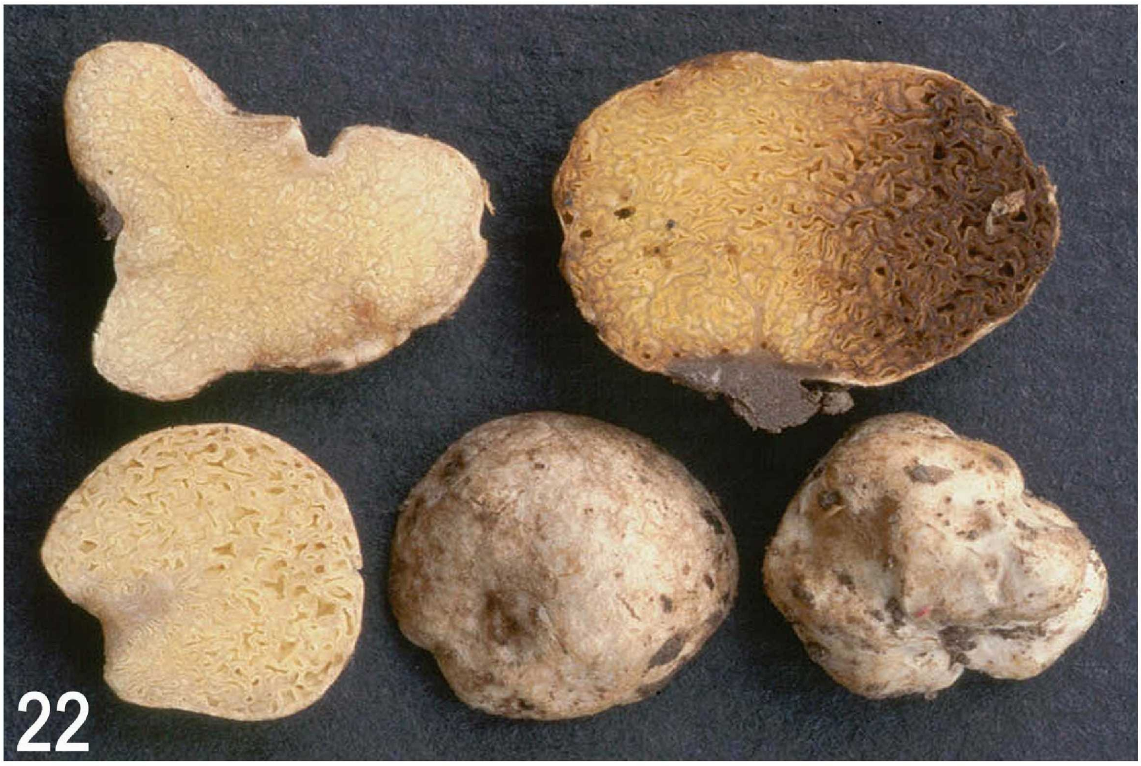

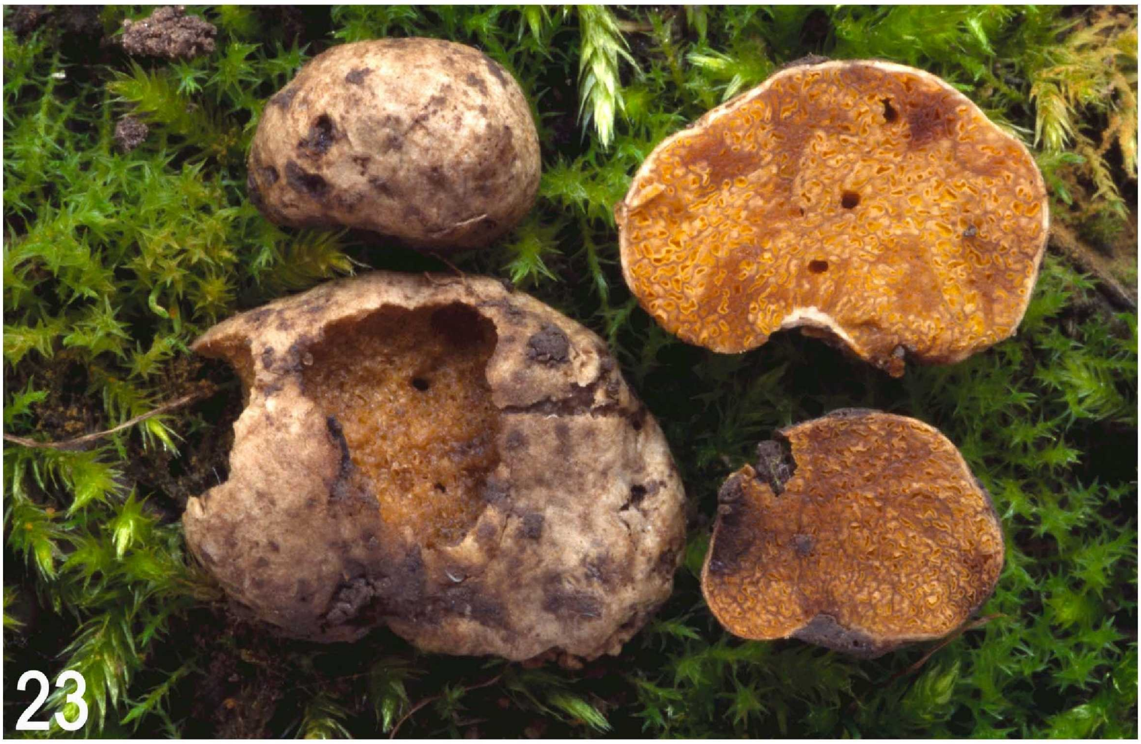

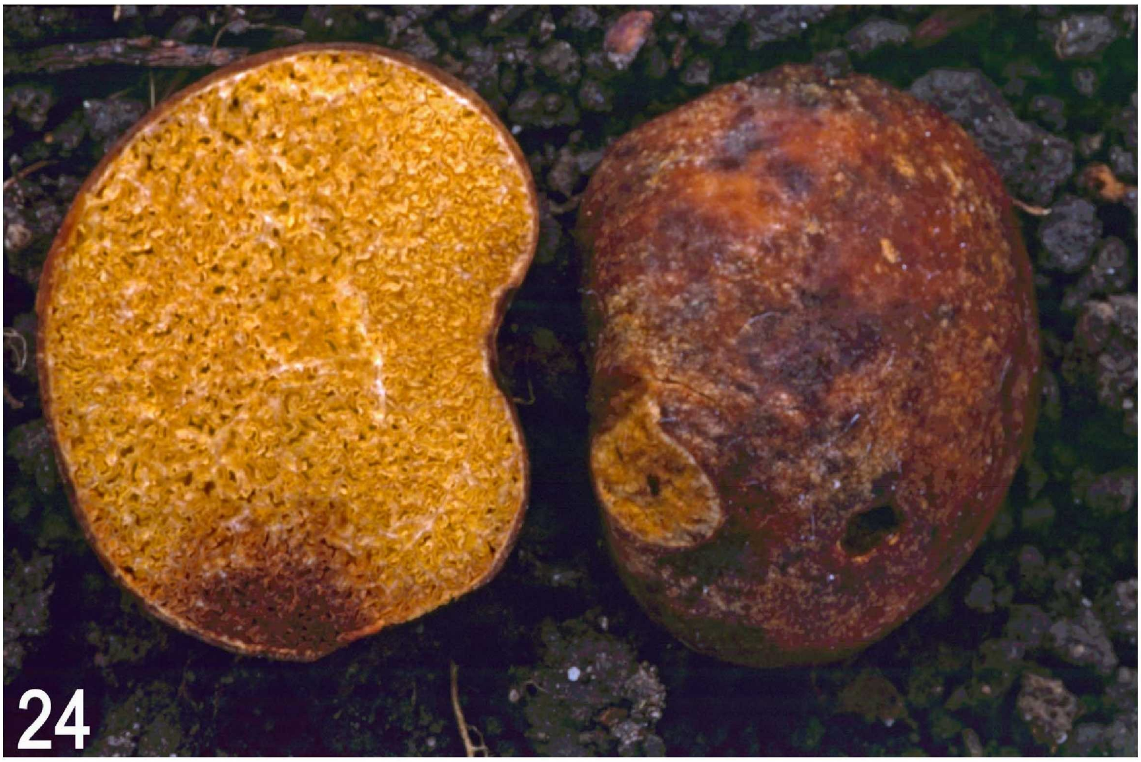

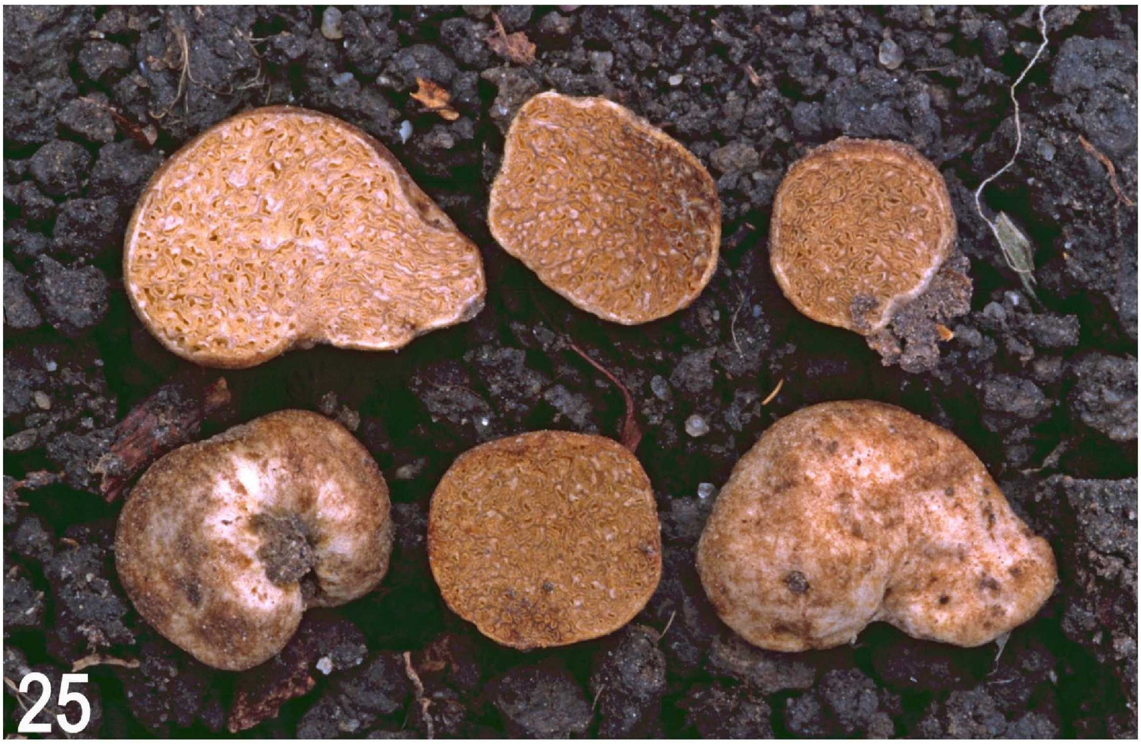
25

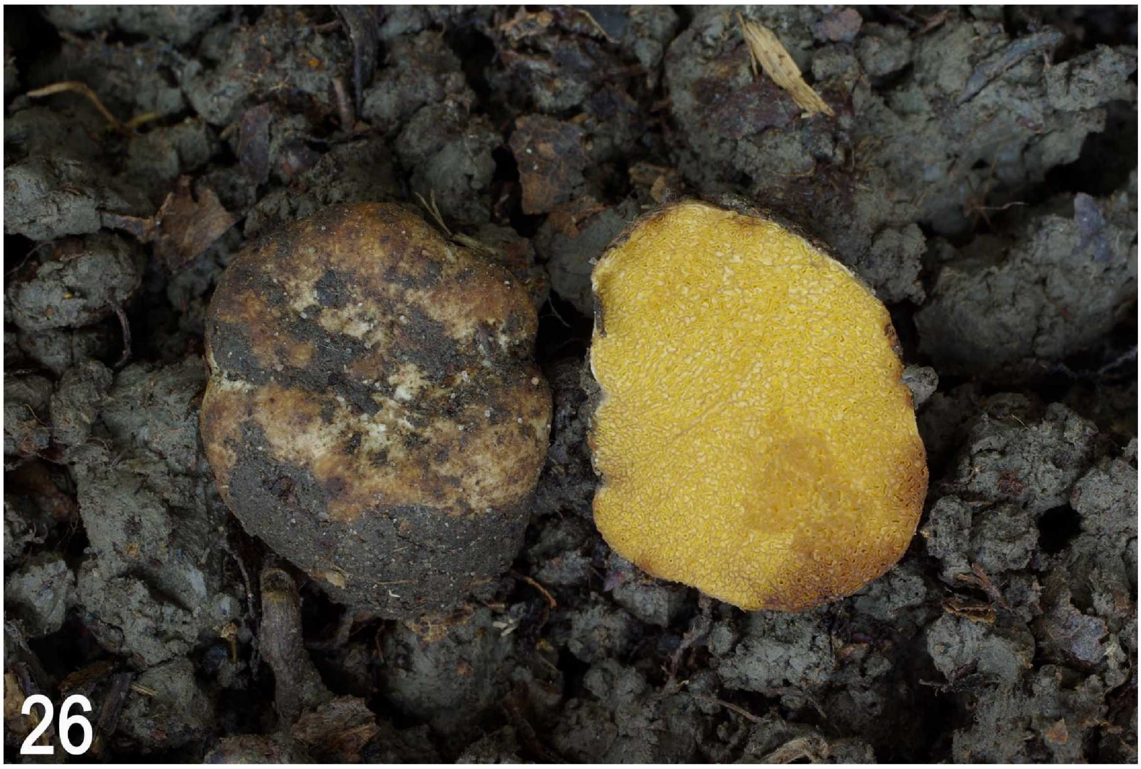
26

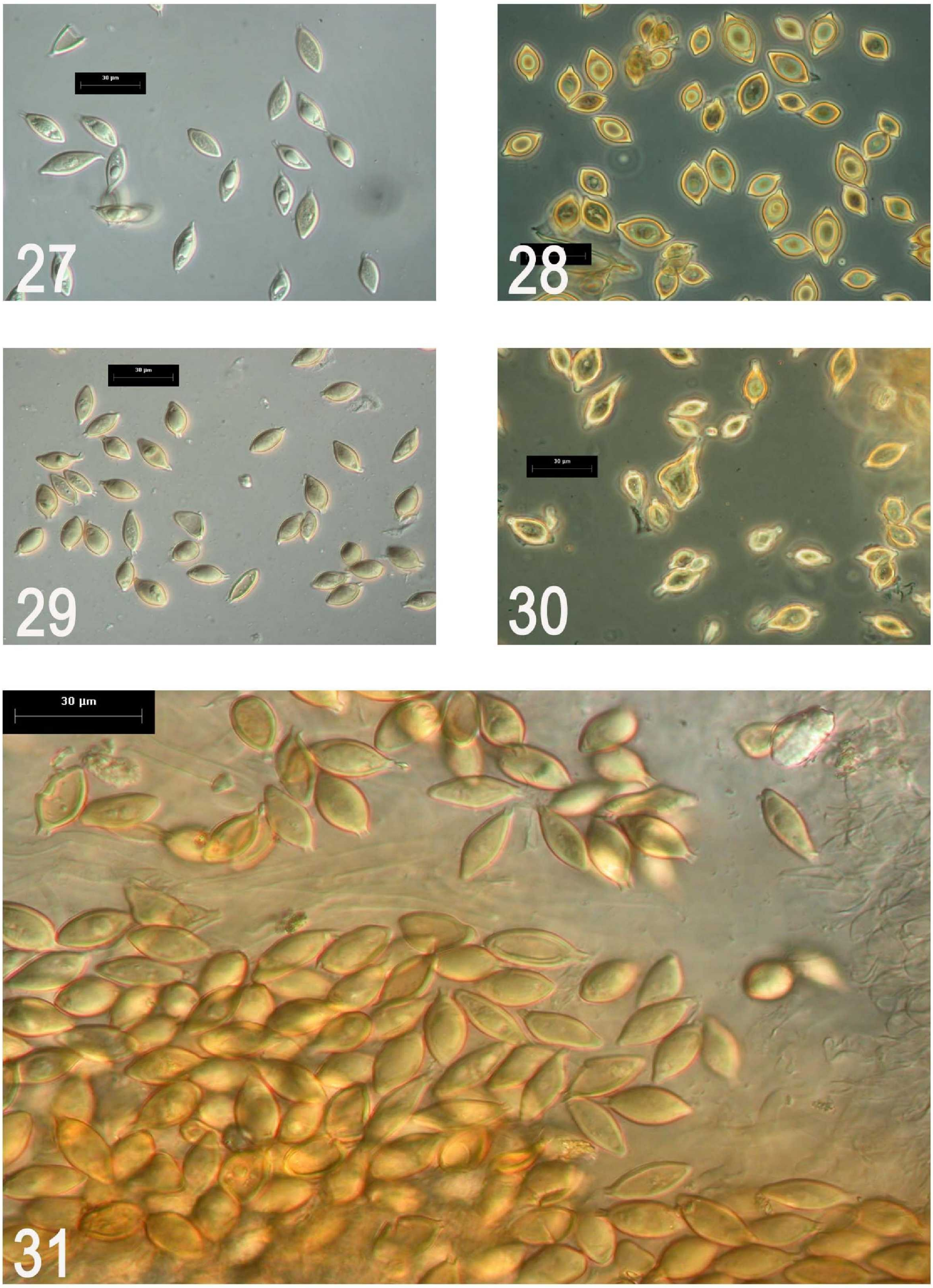
27
28
29
30
30 µm
31

Identification key to *Hymenogaster*

Hymenogaster tener Berk. & Broome 1844

Spores: (13-) 14 (-16) x (8-) 9 (-11) µm in diameter; broad, ovoid to slightly citriform.

Ornamentation: Pronouncedly thorny-spiny; important species-delimitating character (when compared to *H. arenarius, H. niveus* or *H. intermedius*).

Basidiomata: Globose or subglobose, sometimes strongly wrinkled; gouges and cavities present, often with pronounced yellowish dehisced apex, ca. 0.5 - 4 cm in diameter.

Peridia: Brilliant white in juvenile and intermediate stage (image 32), with distinctive yellowish spots at the apex; surface strongly wrinkled; kidney-like at maturity, entirely even when juvenile; colour at maturity turning to pale yellow-brownish-ochraceous.

Gleba: Juvenile to intermediate stages brilliant white; turning grey-brown to rose-brown at full maturity.

Odour: Pleasant, strong floury, *H. tener*-like.

Ecology: The species is strictly found in Central European spring time (Late Jan. – Mar.) under various deciduous trees and soils.

Images: Basidiomata: No. 32. Basidiospores: No. 33.

Identification key to *Hymenogaster*

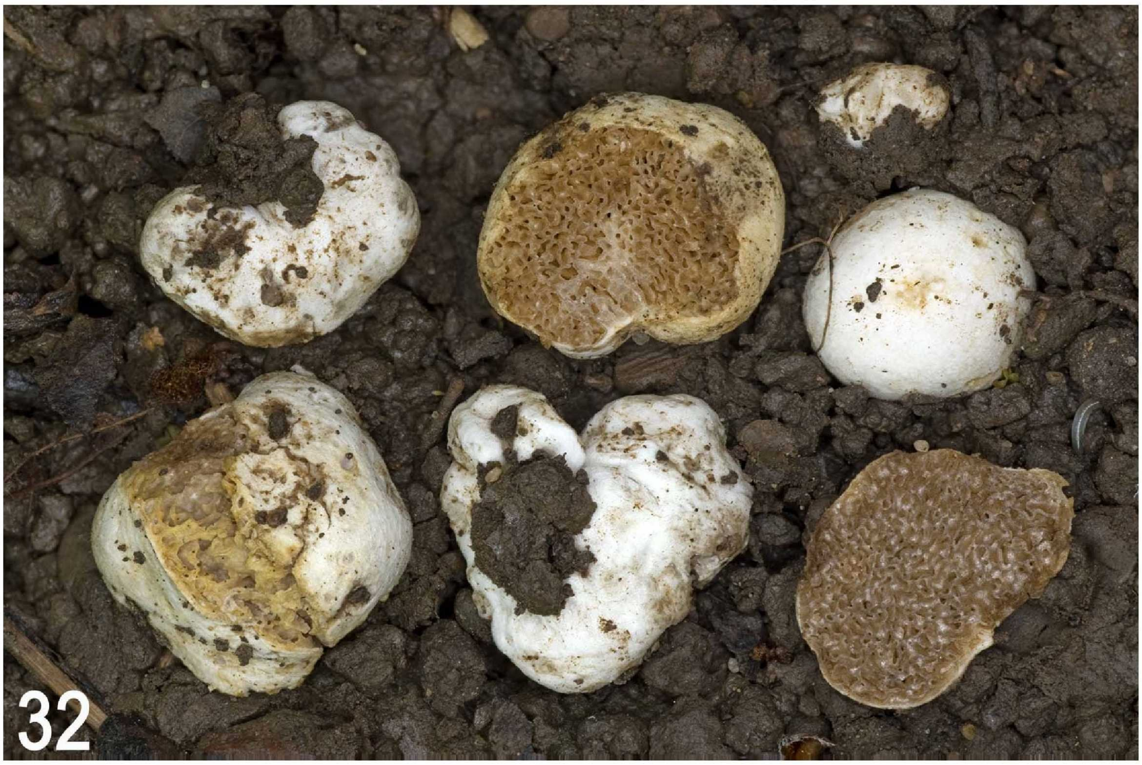

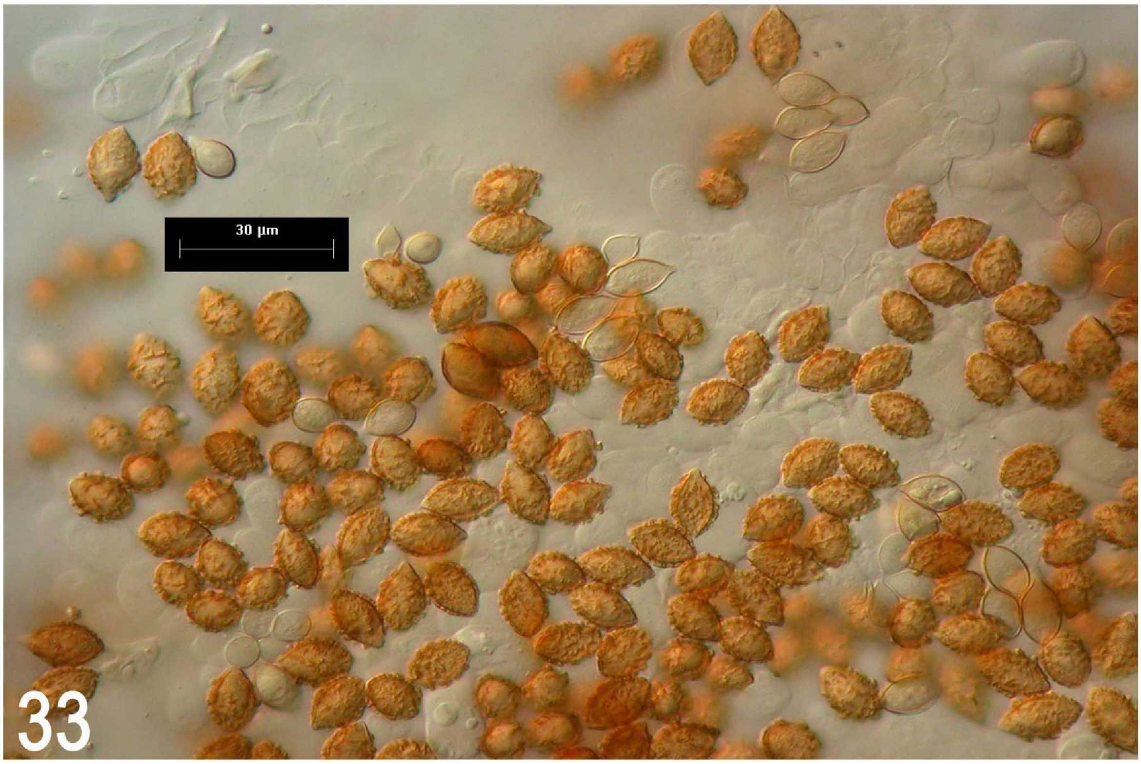

Identification key to *Hymenogaster*

Hymenogaster niveus Vittad. 1831 emend. Stielow et al. 2010

Spores: (9-) 14 (-18) x (6-) 8 (-10) µm in diameter; broad-ellipsoid, citriform, ovoid.

Ornamentation: Warty, spiny, never verrucose.

Basidiomata: Globose or subglobose; often with a pronouncedly dark coloured dehisced apex contrasting *H. tener*; ca. 0.5-2.5 cm in diameter.

Peridia: Smooth, very thin, even, with cavities; colouration predominantly shiny snow-white to pale white (images 34-35); in most cases not changing and present in both juvenile and mature fruiting bodies.

Gleba: Consistency compact, elastic, with small chambers, labyrinthoid; whitish when juvenile, greyish-brownish to dark reddish-black at maturity.

Odour: Rancid, floury but not strikingly unpleasant.

Ecology: Found under various deciduous and coniferous trees in many soil types, from early summer till autumn.

Taxonomic notes: *H. niveus* sensu stricto was found to be genetically very heterogeneous but stable regarding its morphological characters. *H. niveus* consists of at least six cryptic species that require a more detailed analysis and verification. The insufficient number of exsiccates analysed in our dataset did not allow to further separate this very difficult species complex. In the future, morphological differences between the cryptic species might be found regarding spore shape (quotient Q of spore length and width).

Images:

- Basidiomata: No. 34-35. Basidiospores: No. 36-42.

Identification key to *Hymenogaster*

- Cryptic species one (= cluster 0): spores (9-) 14 (-18) x (6-) 10 (-13), Q = 1.4 (images 36-37, 40-41).
- Cryptic species two (= cluster 15): spores (10-) 13 (-16) x (6-) 9 (-11), Q = 1.44
- (image 43).
- Cryptic species three (cluster 3): spores (15-) 16 (-18) x (10-) 11 (-12), Q = 1.45.
- Cryptic species four (cluster 22): spores (10-) 14 (-17) x (8-) 11 (-15), Q = 1.27
- (images 38-39).
- Cryptic species five (cluster 21): spores (9-) 12 (-14) x (7-) 9 (-12), Q = 1.33.
- Cryptic species six (cluster 7): spores (10-) 12 (-14) x (7-) 9 (-10), Q = 1.33.

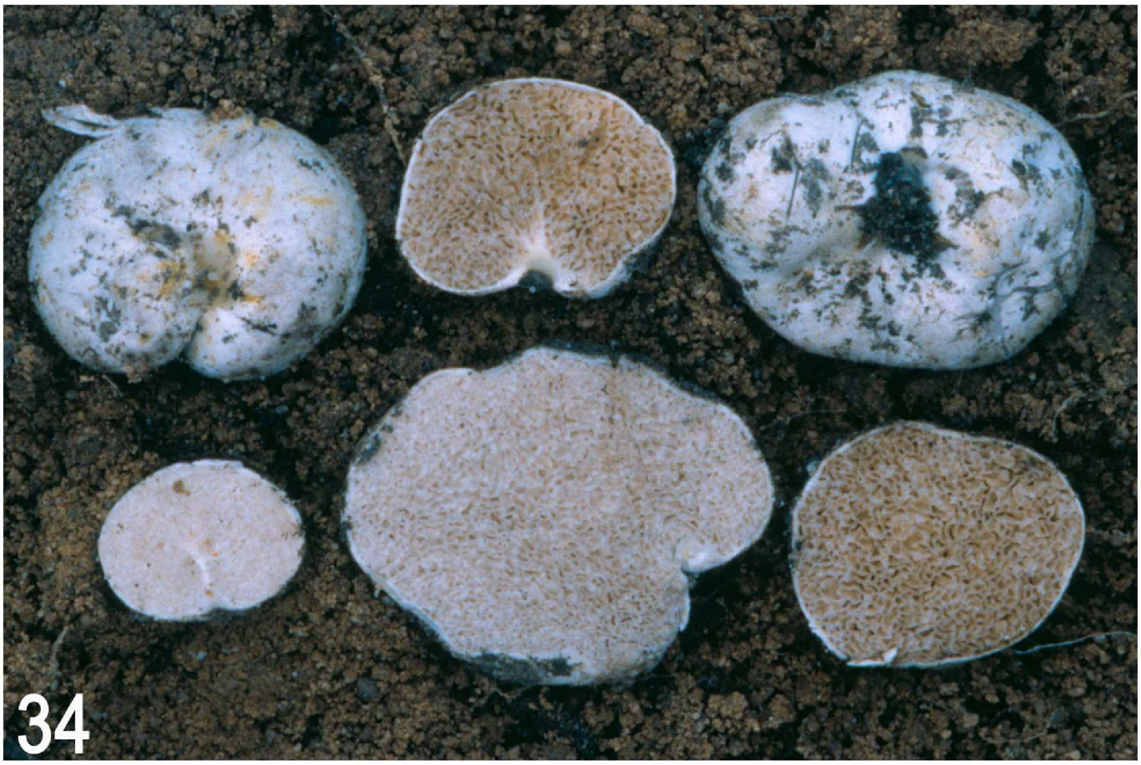
34

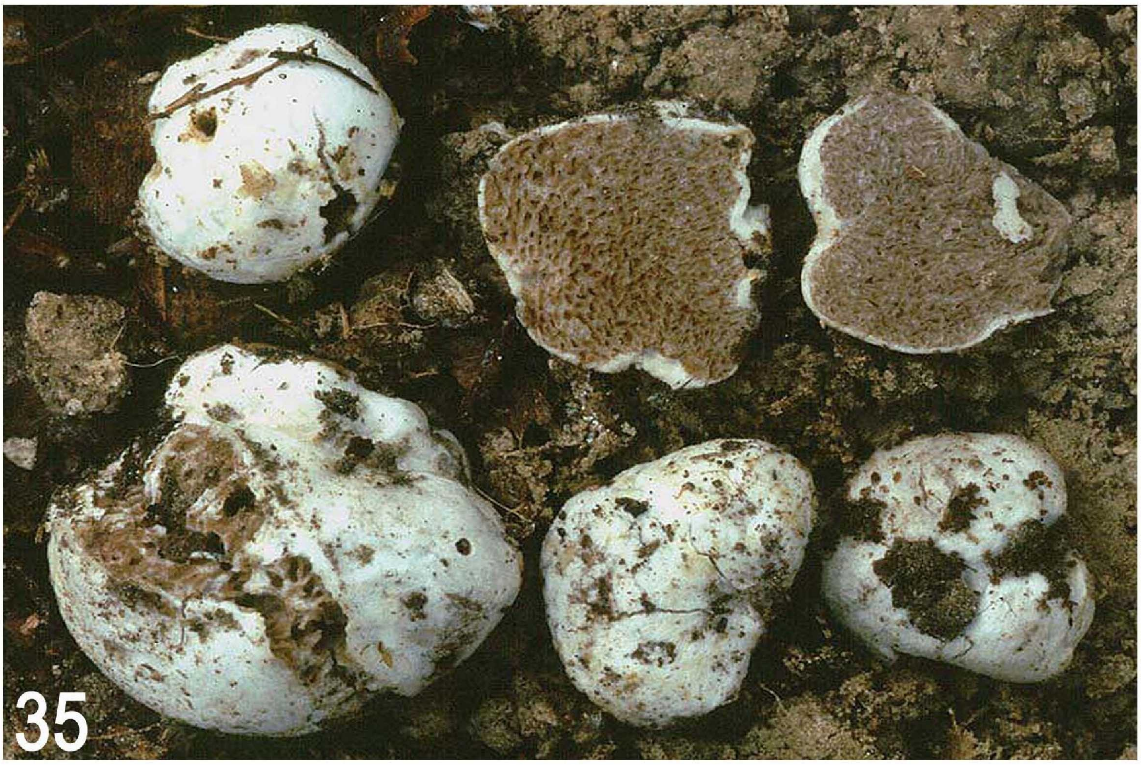
35

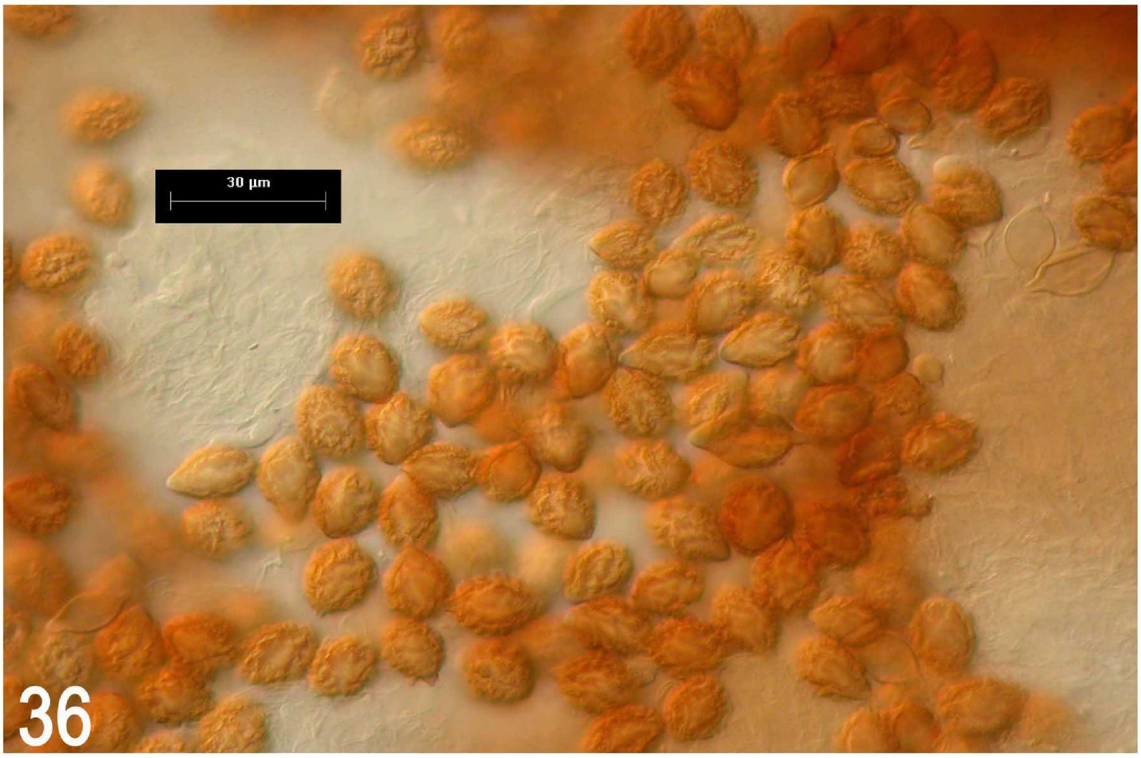
30 µm
36

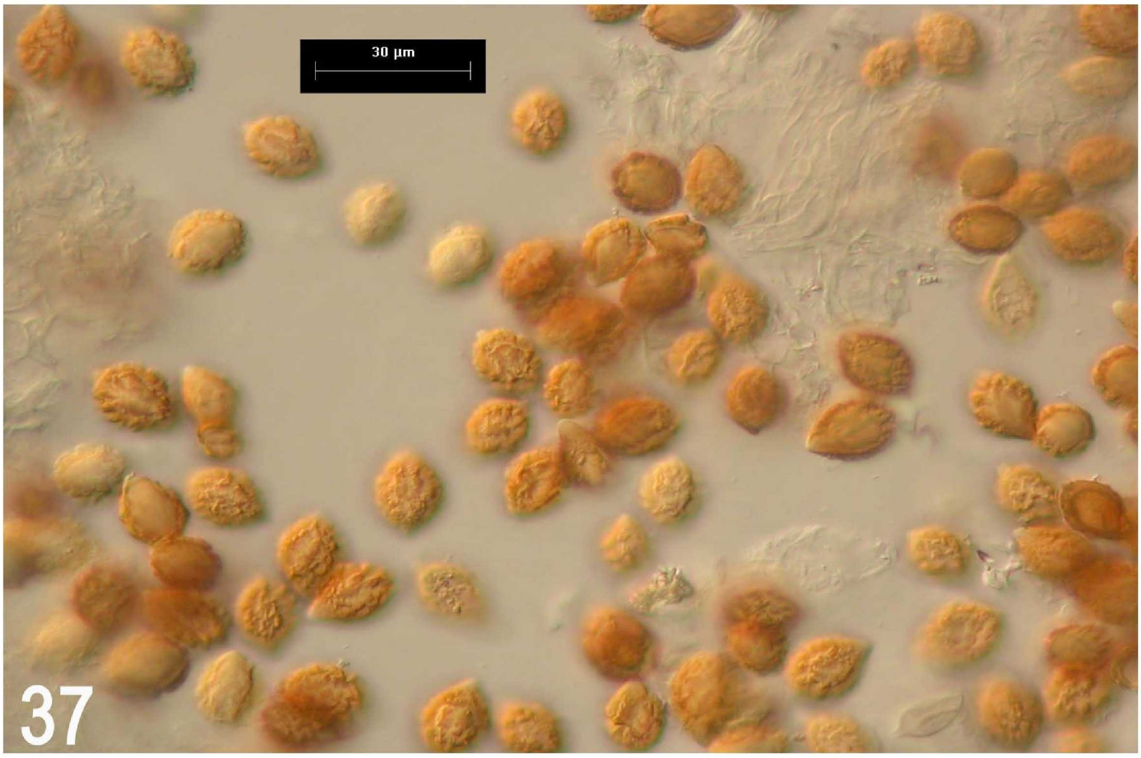
30 µm
37

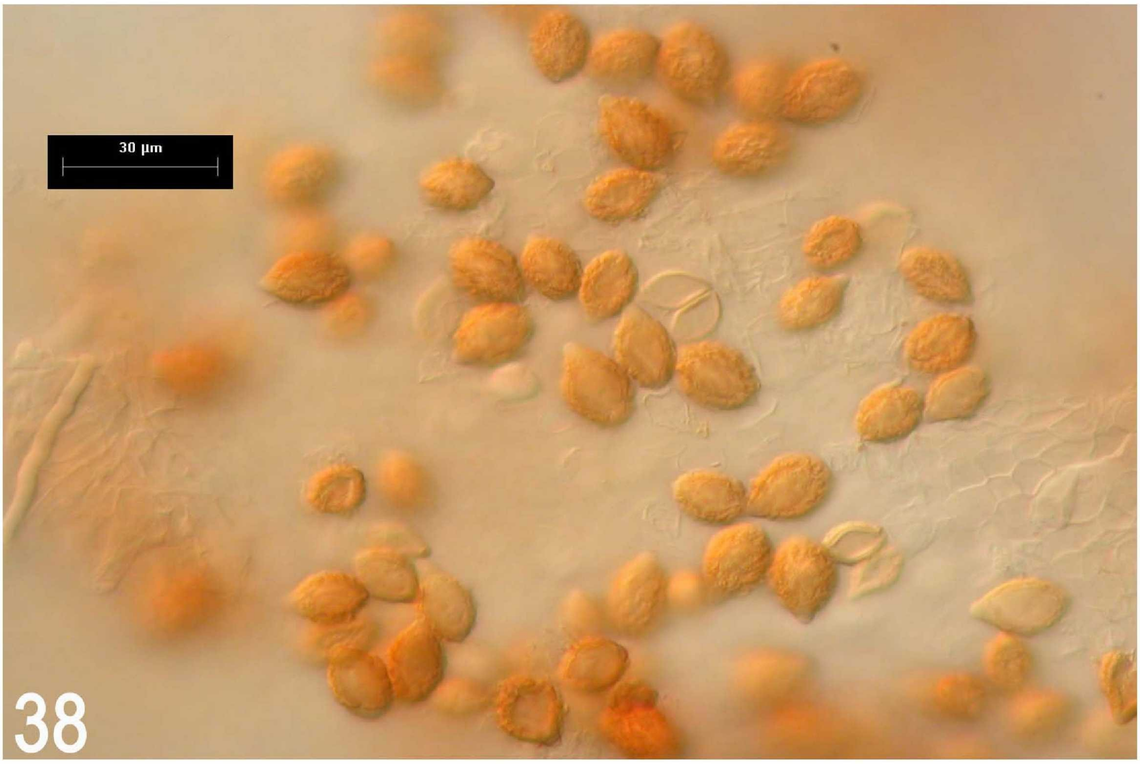
30 µm
38

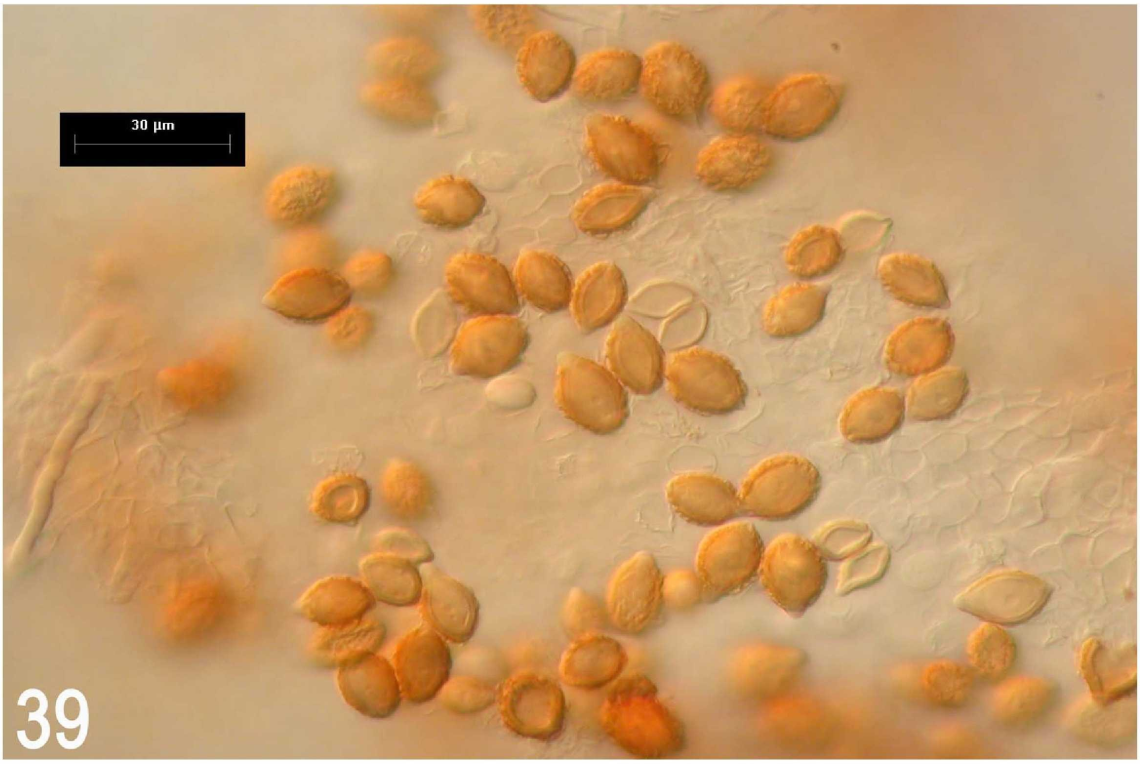
30 µm
39

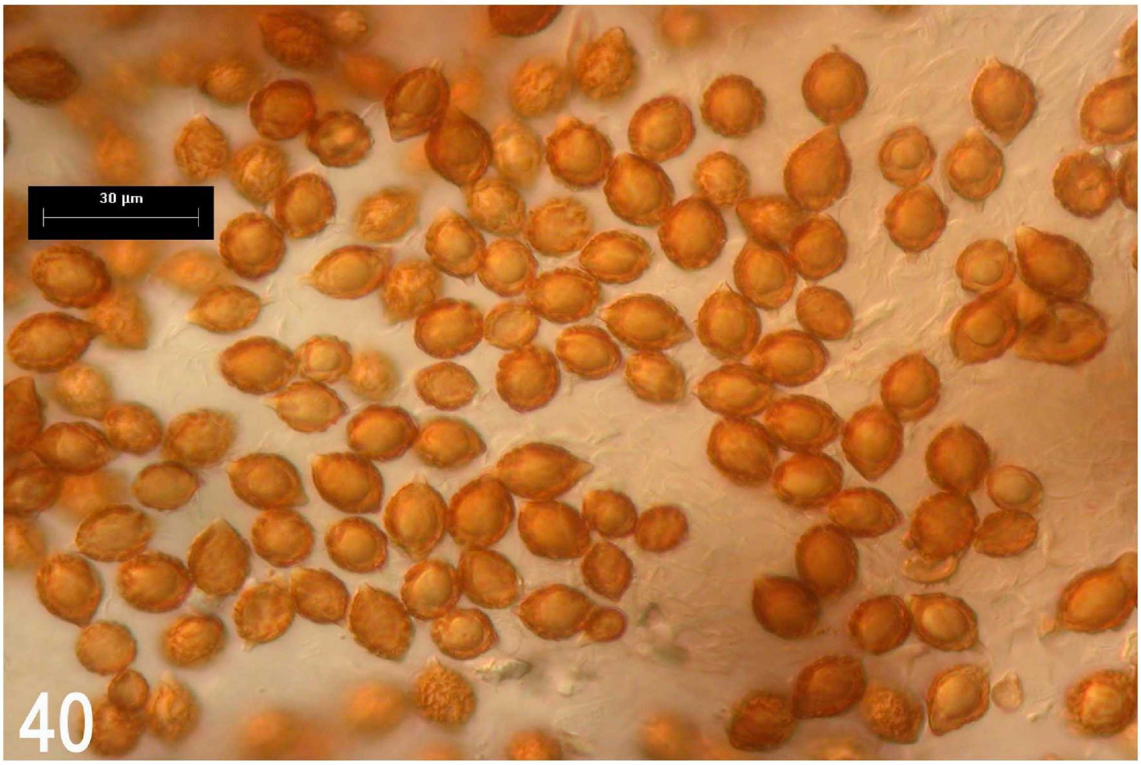
30 µm
40

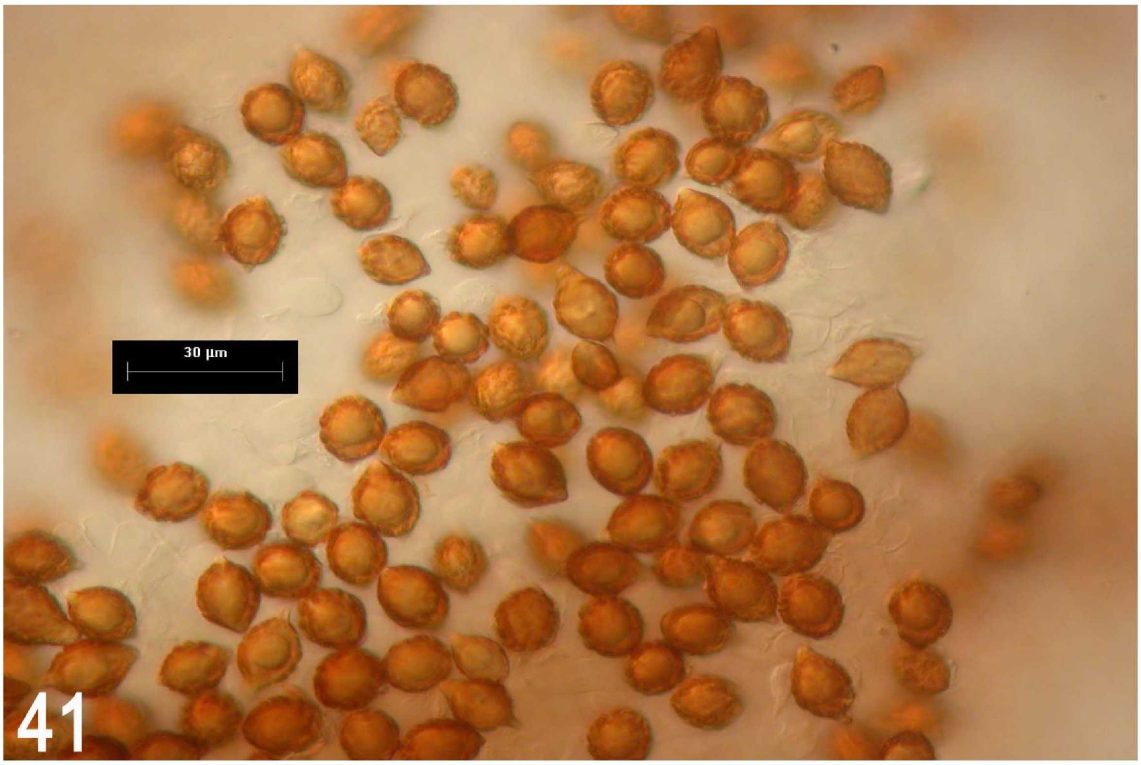
30 µm
41

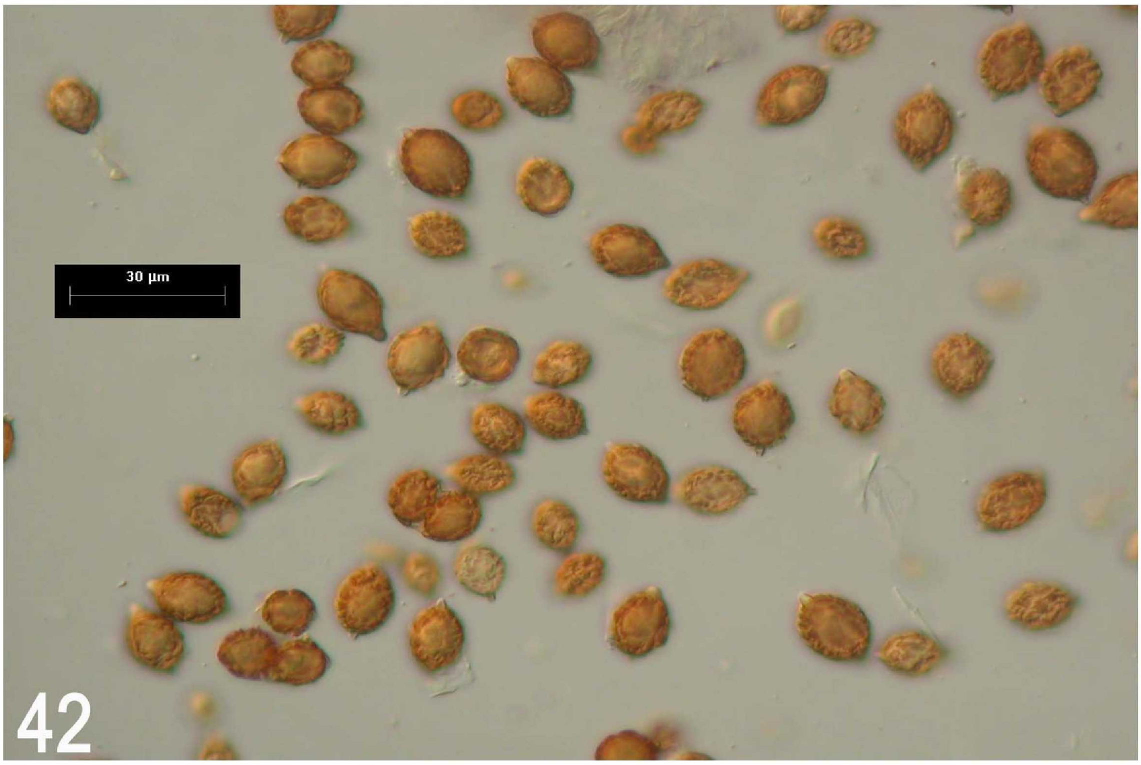
30 µm
42

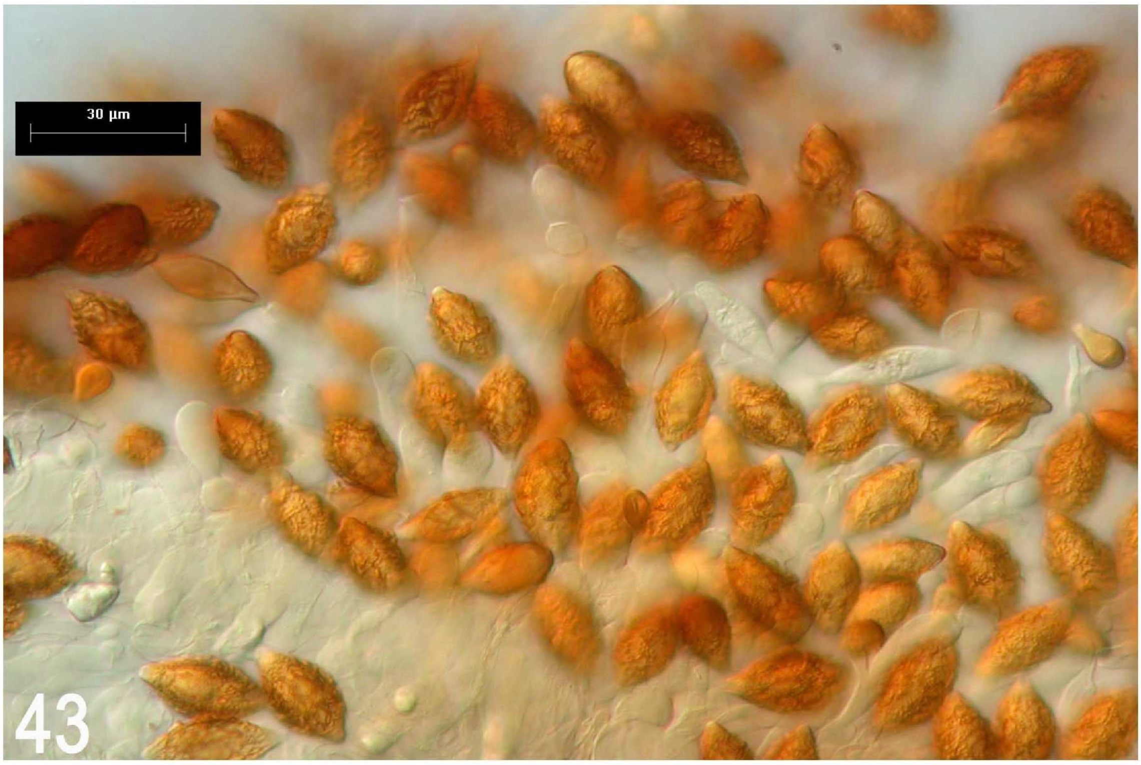
30 µm
43

Identification key to *Hymenogaster*

Hymenogaster arenarius Tul. & C. Tul. 1844

Spores: (10-) 14 (-17) x (6-) 10 (-13) µm in diameter; broad, ovoid-citriform; fusoid deviations present.

Ornamentation: Warty, thorny, never verrucose.

Basidiomata: Globose, subglobose, wrinkled, ca. 0.5-2.5 cm in diameter.

Peridia: Pale grey-whitish when juvenile, with brown tones, surface greasily-waxy-shiny.

Gleba: Consistency compact; composed of small chambers, labyrinthoid; emerging radially from a sterile base; greyish-whitish to rose-greyish when juvenile; brownish-carmine and rusty-red tones at maturity; trama appears waxy.

Odour: Very unpleasant, reminding of putrescent garlic or onion.

Ecology: The species is found under various deciduous trees, often in park-like habitats; it prefers loose sandy soils. Typical *Hymenogaster* species in Central European late spring time, never grows in the summer season.

Images: Basidiomata: No. 44-47, 54-55. Basidiospores: No. 48-53, 56-61.

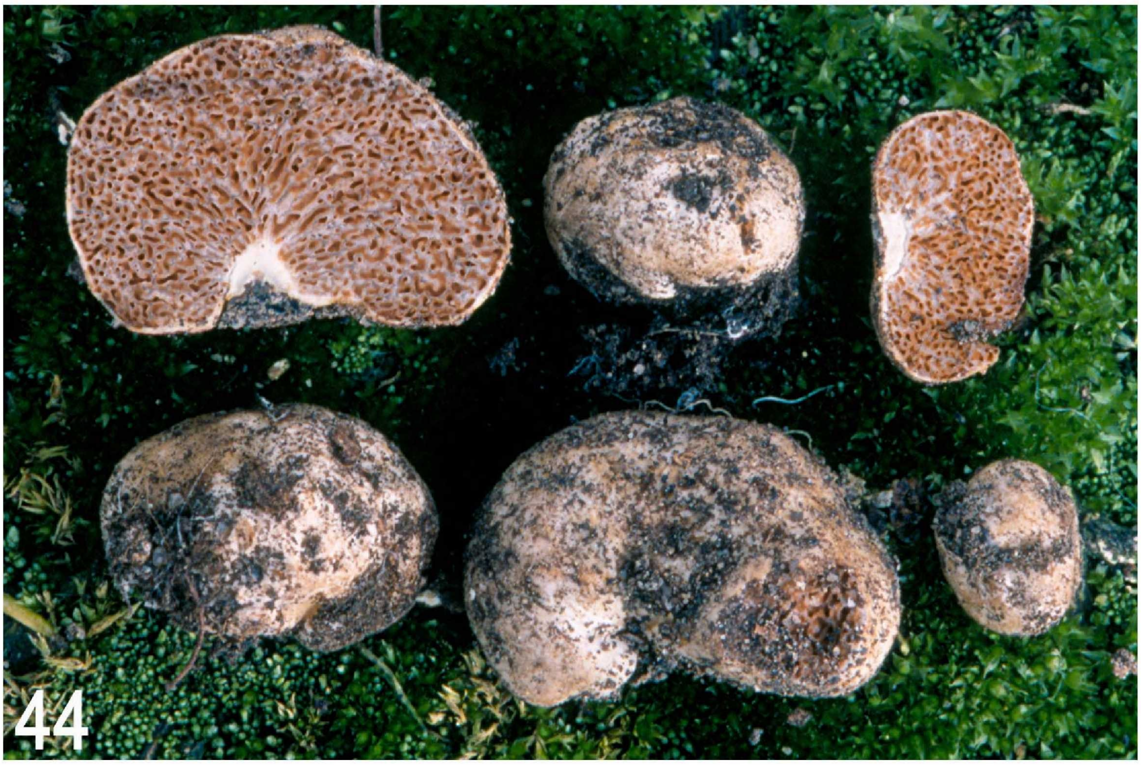
44

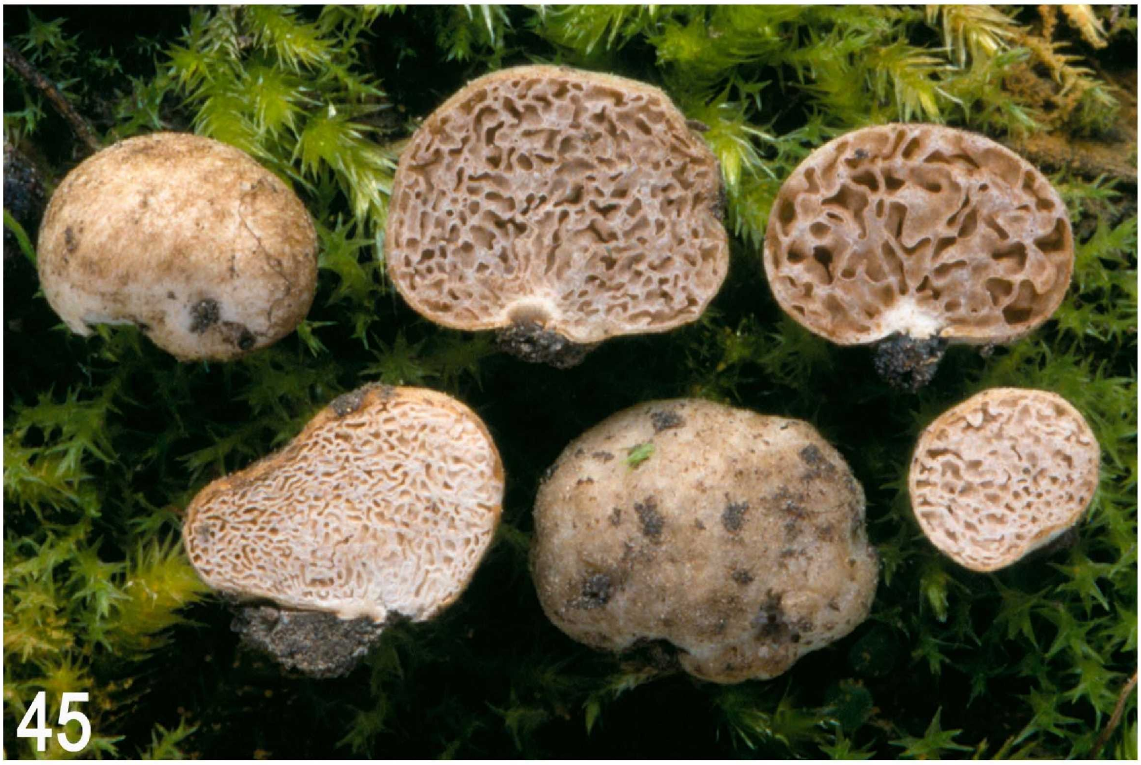
45

Identification key to *Hymenogaster*

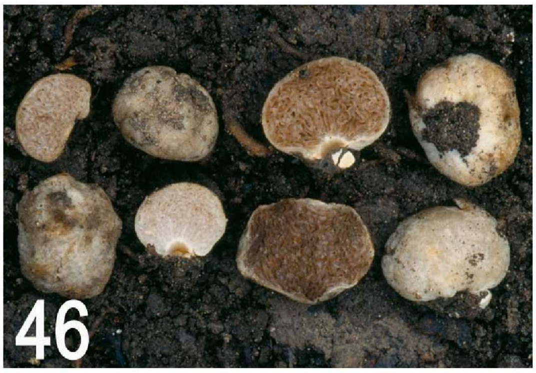

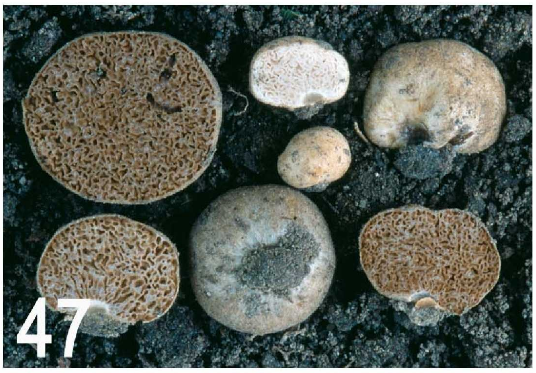

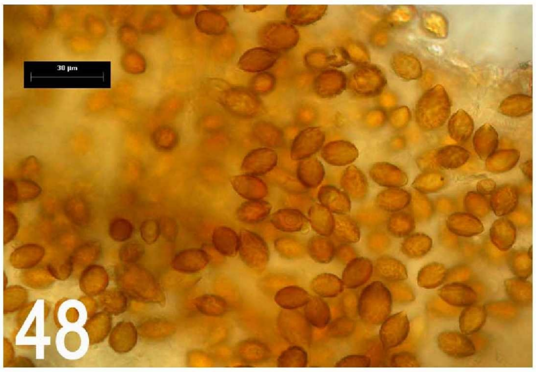

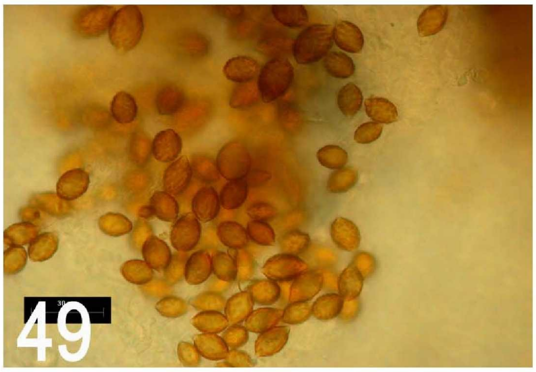

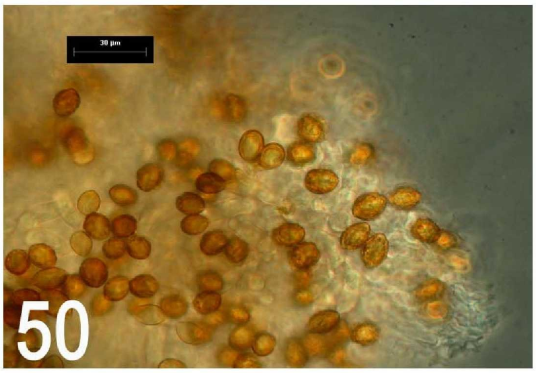

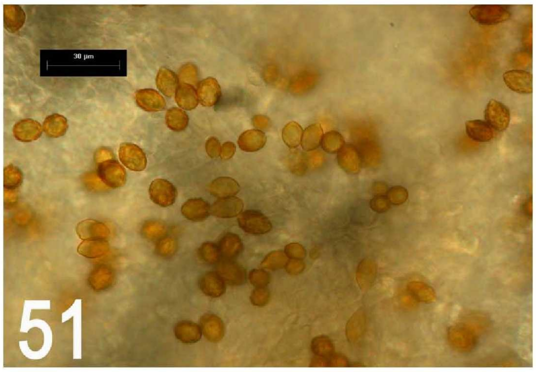

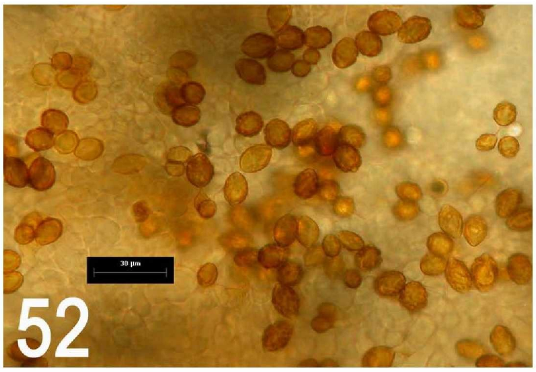

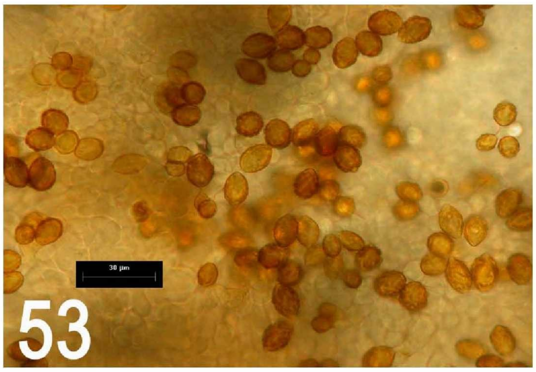

Identification key to *Hymenogaster*

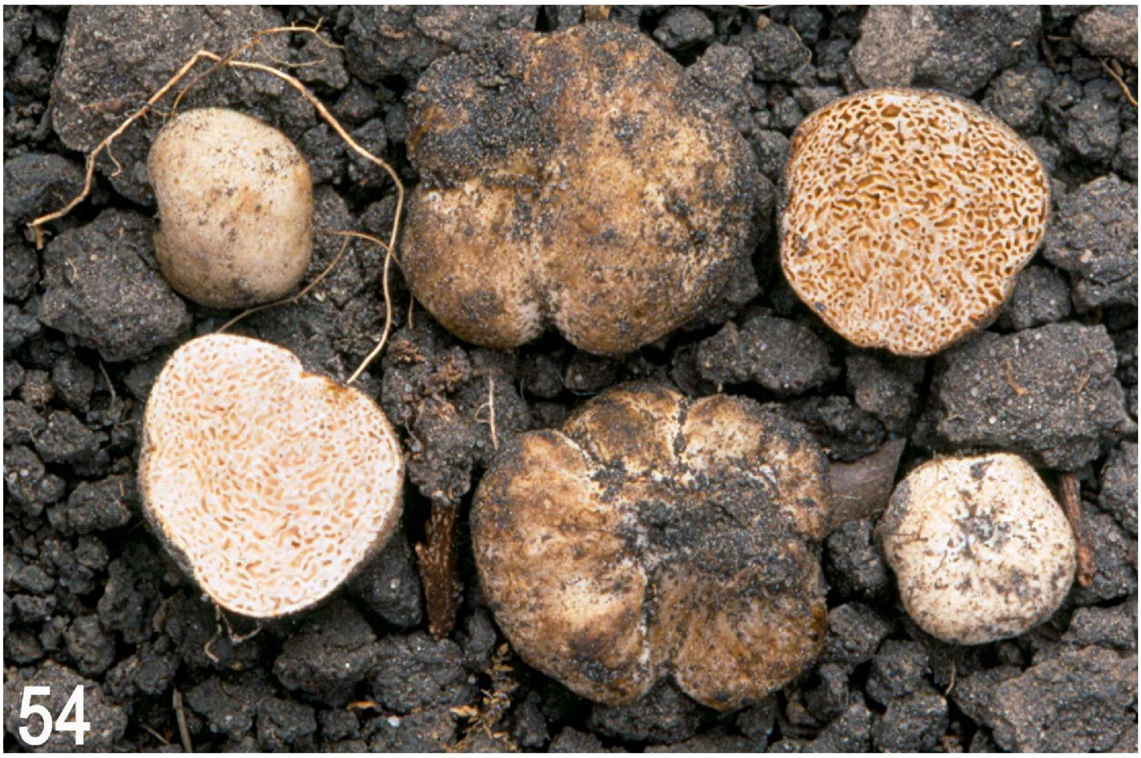

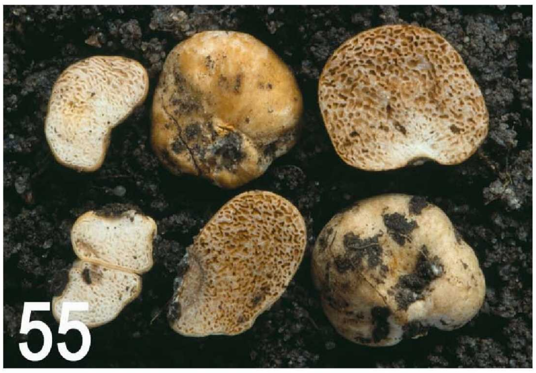

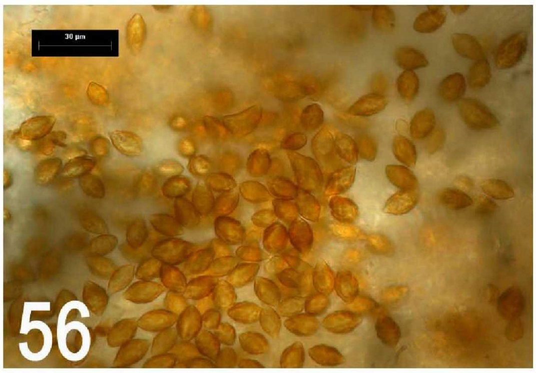

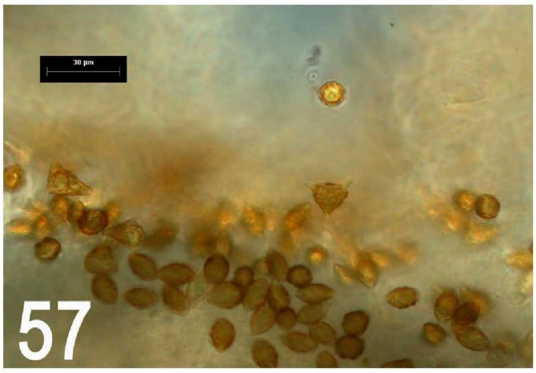

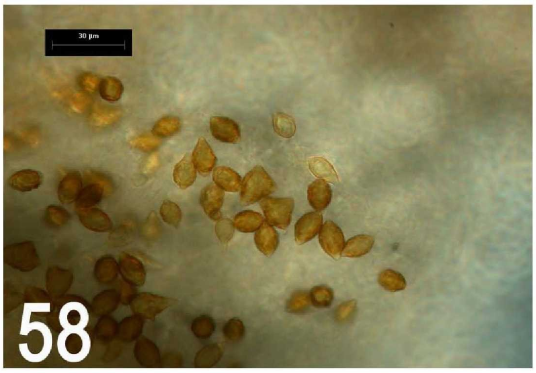

Identification key to *Hymenogaster*

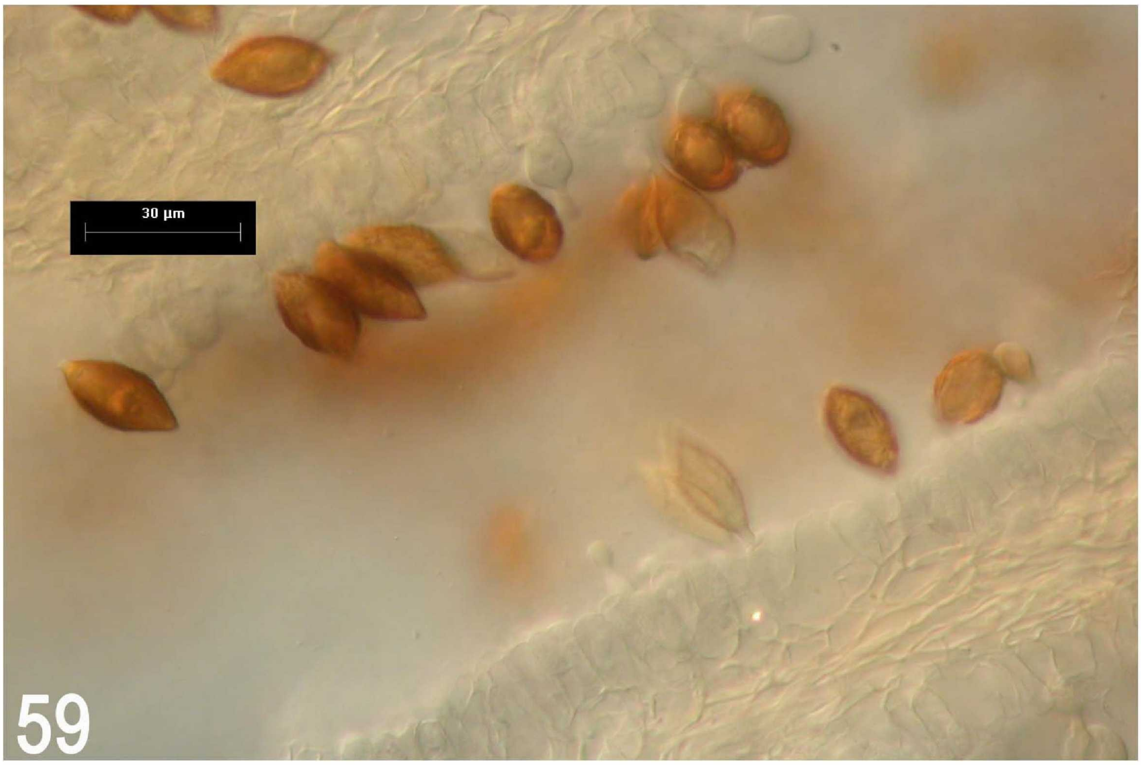

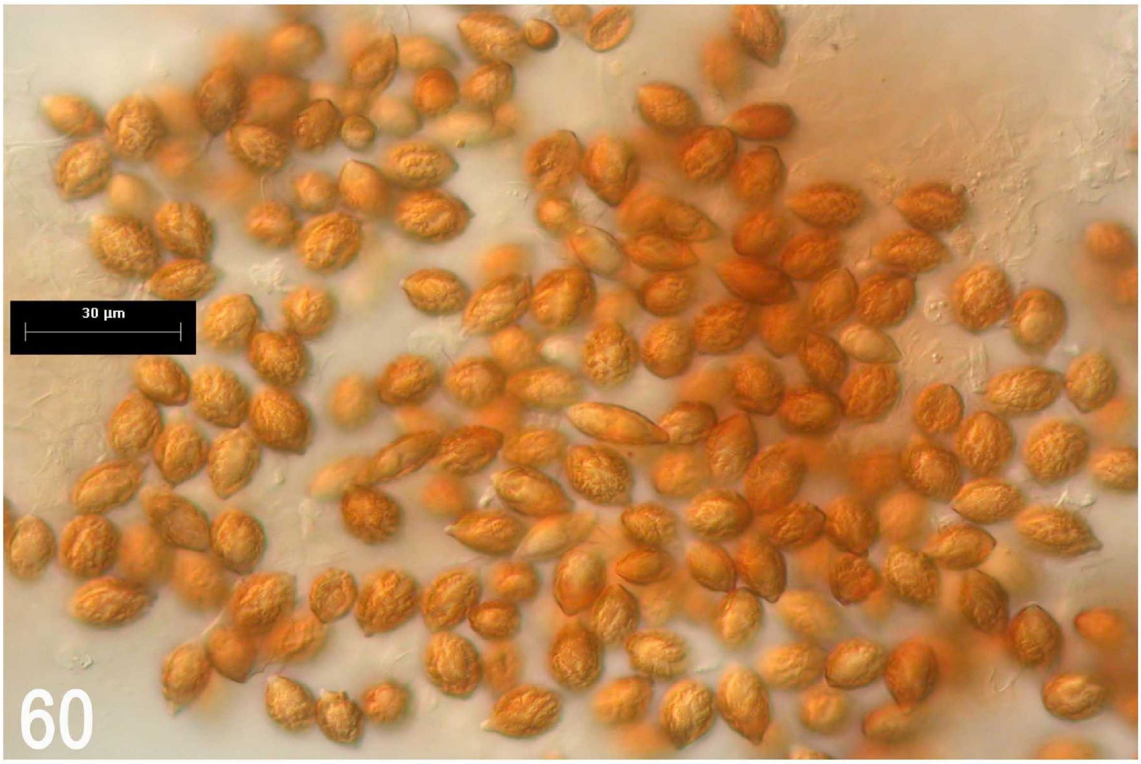

Hymenogaster intermedius Stielow et al. 2010, sp. nov.

Spores: Spores (10-) 12 (-14) x (6-) 8 (-9) µm in diameter; globose to subglobose or ovoid, spores yellow-brown.

Ornamentation: Very similar to *H. arenarius* and *H. niveus*, not spiny but rather thorny-verrucose.

Basidiomata: Tuberous, globose to subglobose; ca. 1 - 1.5 cm in diameter.

Peridia: Plain white when young, turning to pale-ochraceous in mature basidiomes, very similar to the typical colouration of *H. rehsteineri*; change of colouration very slow.

Gleba: White when juvenile, passing through gentle lilac tones, turning to brilliant warm-brown at maturity, but remaining more light-coloured than in typical *H. rehsteineri* specimens. Columella present, with mycelial cord at the base.

Odour: Floury- or cucumber like, pleasant.

Ecology: Present under various deciduous trees, so far reported from northern and eastern Germany to be only associated with *Fagus sylvatica*, in Hungary with various host trees. Very rare taxon.

Images: Basidiomata: No. 62. Basidiospores: No. 63-65.

Identification key to *Hymenogaster*

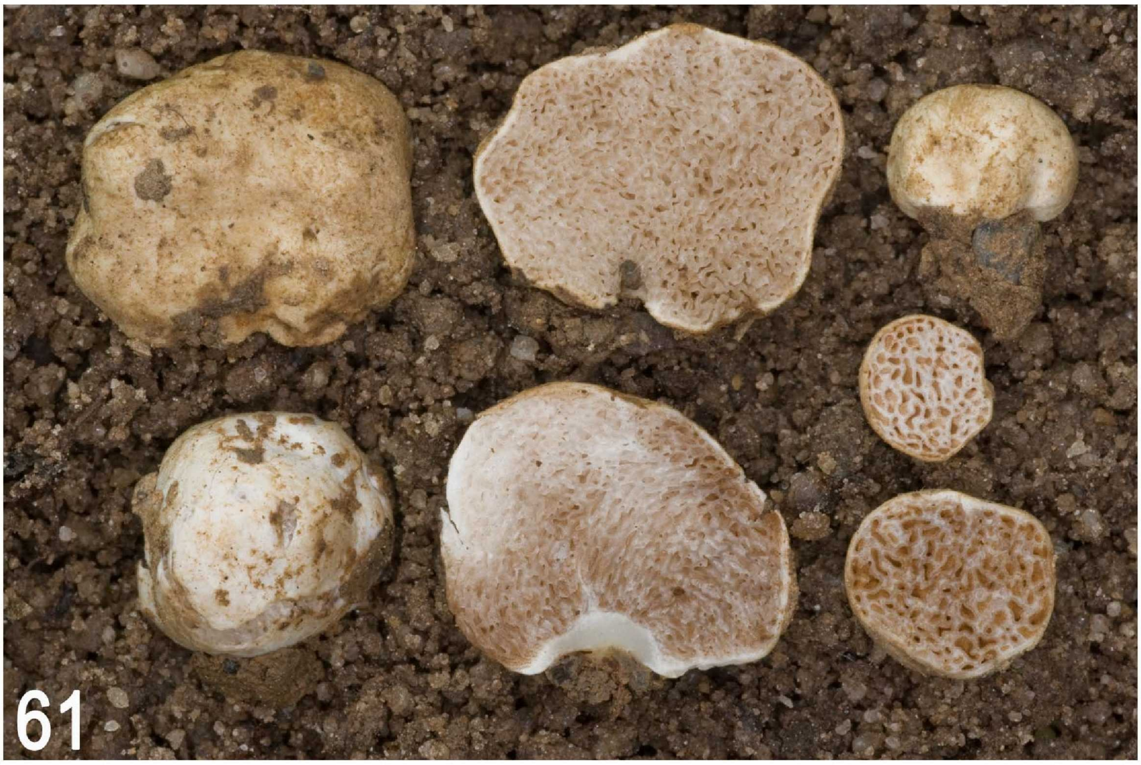

61

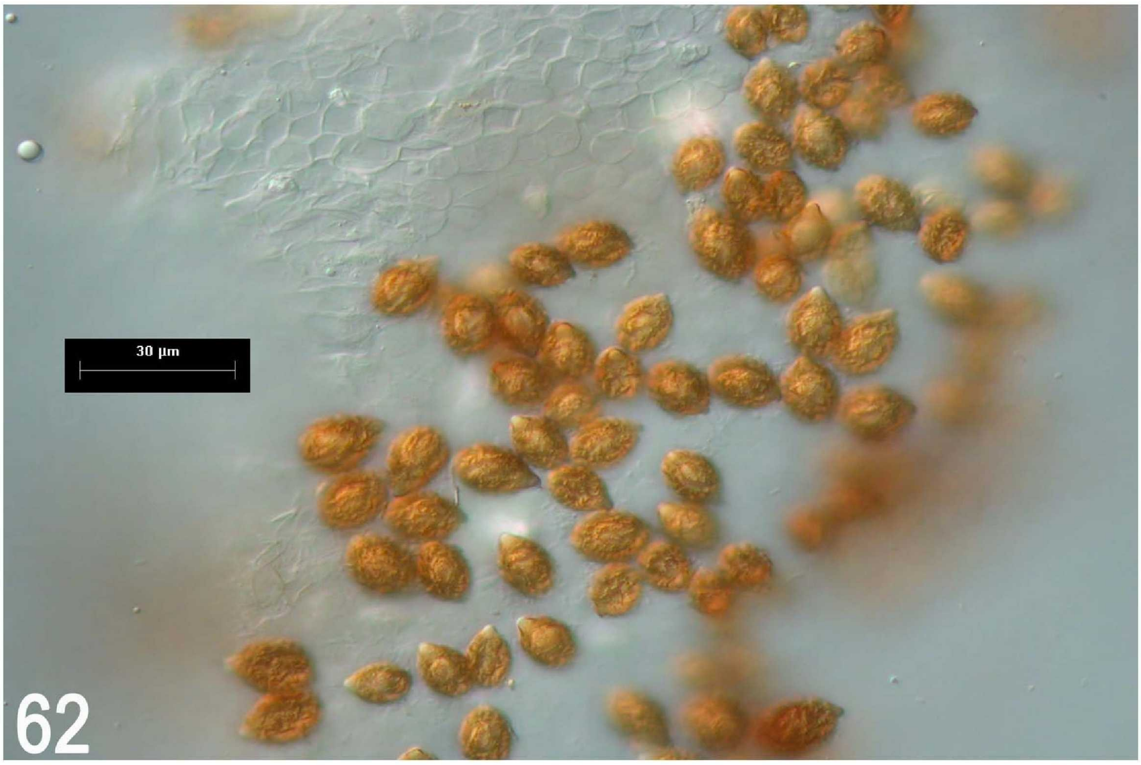

62

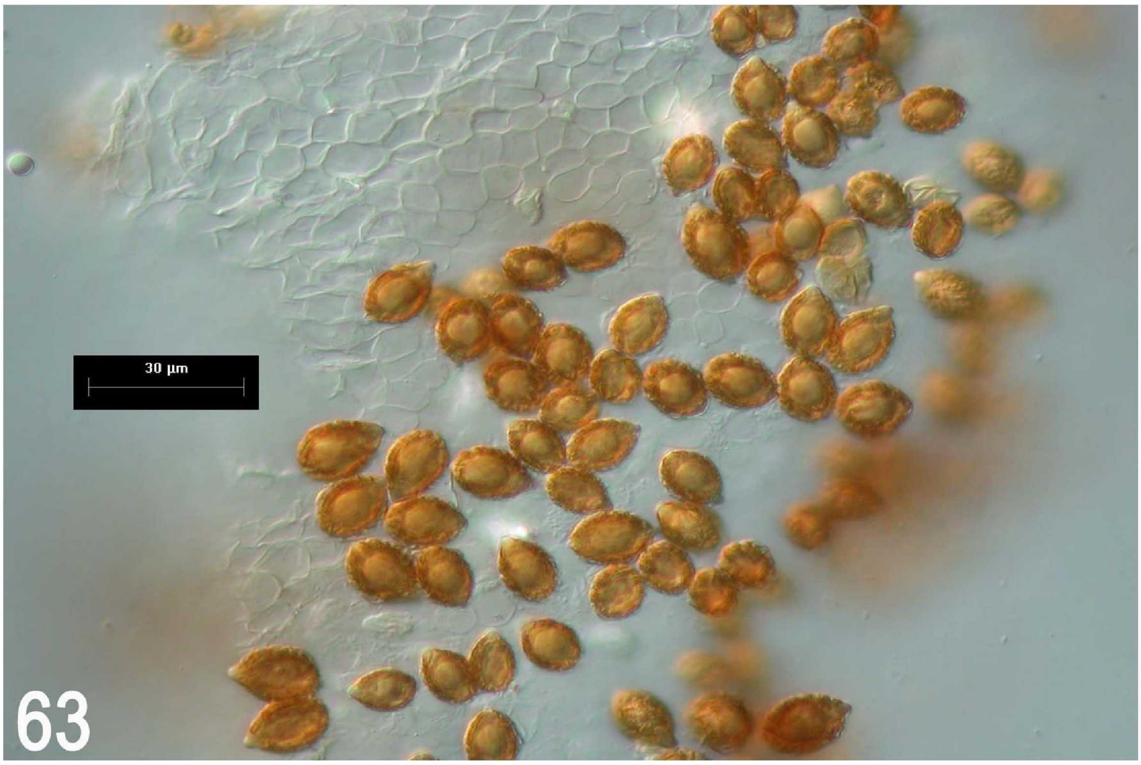
30 µm
63

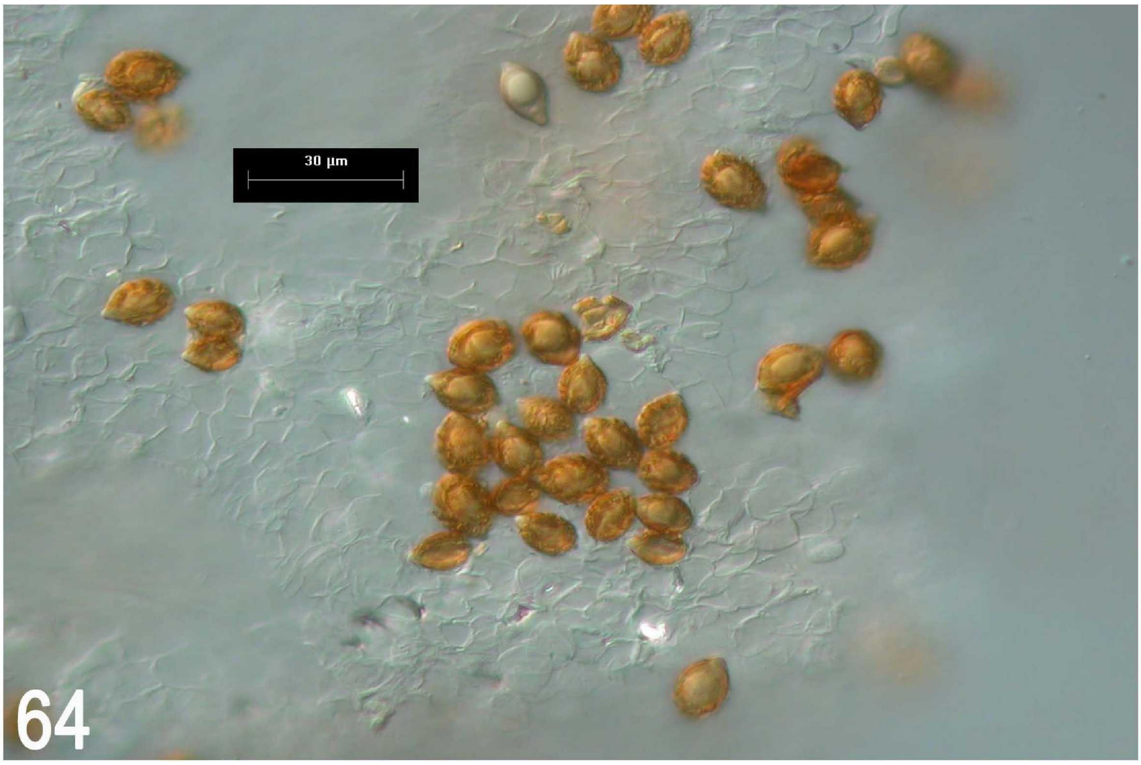
30 µm
64

Identification key to *Hymenogaster*

Hymenogaster thwaitesii Berk. & Broome 1846

Spores: (10-) 21 (-29) x (7-) 13 (-24) µm in diameter; predominantly globose-subglobose to ovoid (Q= 1.6), but mucronate-fusoid spores always present.

Ornamentation: Verrucose to wrinkled.

Basidiomata: Globose, knotted, lobated; usually very small, ca. 0.5 - 1 cm in diameter.

Peridia: Uneven, surface wrinkled; whitish-yellowish when juvenile, yellowish-brownish at maturity, with all possible brown tones during the ripening process.

Gleba: Compact, not compressed; enduring small chambers, labyrinthoid; yellowish-beige when juvenile, dark-brown to cinnamon at maturity.

Odour: Soil- or swamp-like.

Ecology: Under deciduous and coniferous trees and in various soils, also in montane habitats. Often at wet, shady sites. Very rare taxon across Europe.

Images: Basidiomata: No. 66. Basidiospores: No. 67-70.

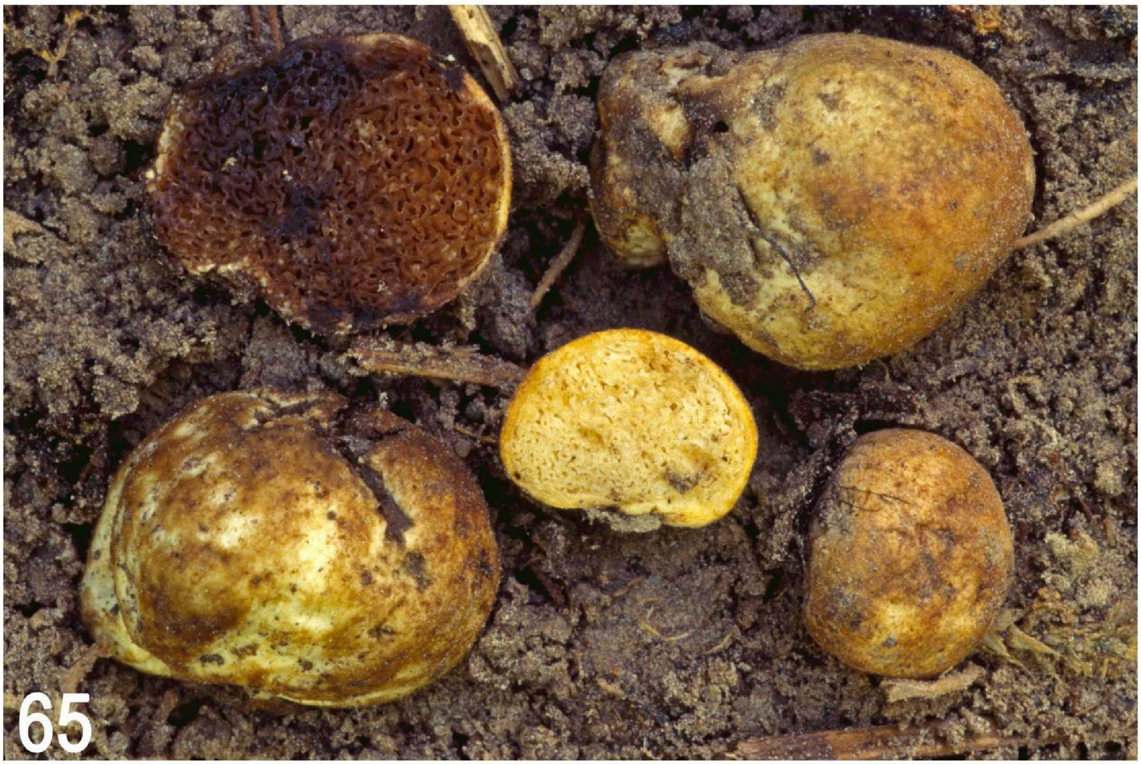
65

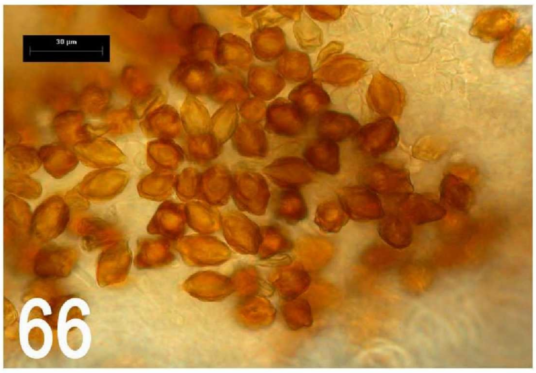
66

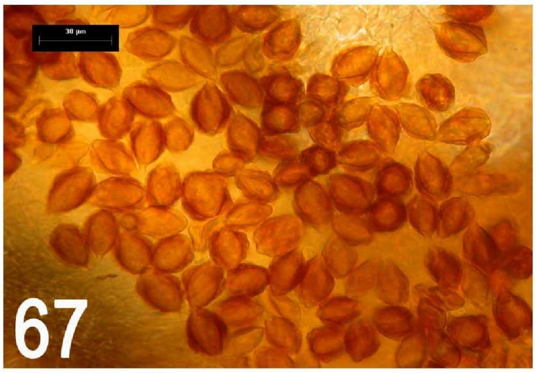
67

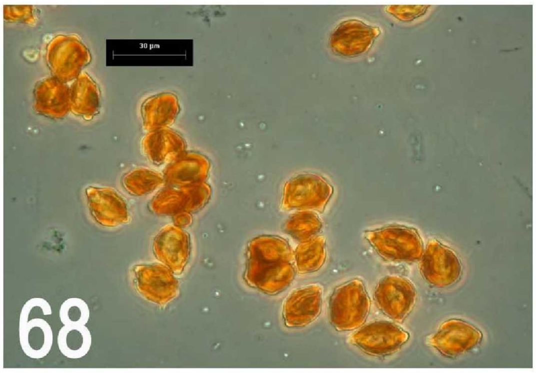
68

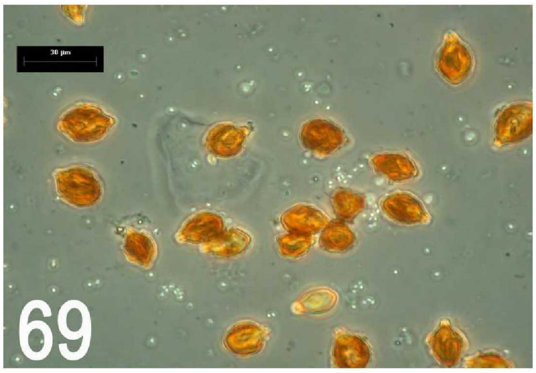
69

Identification key to *Hymenogaster*

Hymenogaster megasporus Soehner 1952

Spores: (15-) 25 (-37) x (6-) 13 (-18) µm in diameter; elongated, fusoid-lanceolate, spores unusually large on average in some exsiccates > 35 µm.

Ornamentation: Vigorously verrucose (similar to *H. griseus*).

Basidiomata: Globose, roundish; ca. 0.5 -1 cm in diameter.

Peridia: Surface slightly uneven, but not wrinkled, furrowed or with cavities; whitish-grey when juvenile turning grey-brown-purple with predominant lilac tones at maturity.

Gleba: Consistency very compact, with large chambers, not compressed, labyrinthoid orientation; with constant and predominant lilac and blue purple tones during ripening (rare character state in other *Hymenogaster* species); this distinct colouration is independent of the degree of maturity; juvenile gleba whitish-purple.

Odour: Insignificant.

Ecology: A species found under various deciduous trees, ultra rare taxon or rarely found across Europe.

Images: Basidiomata: No. 71. Basidiospores: No. 72-74.

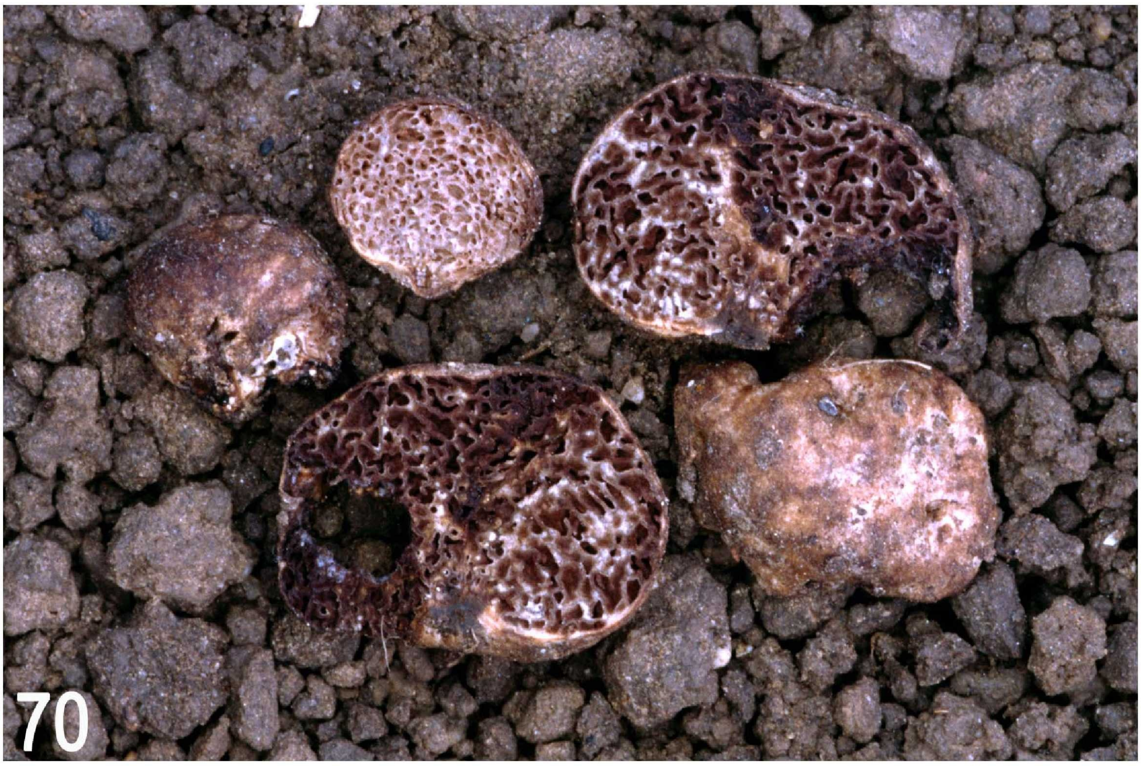
70

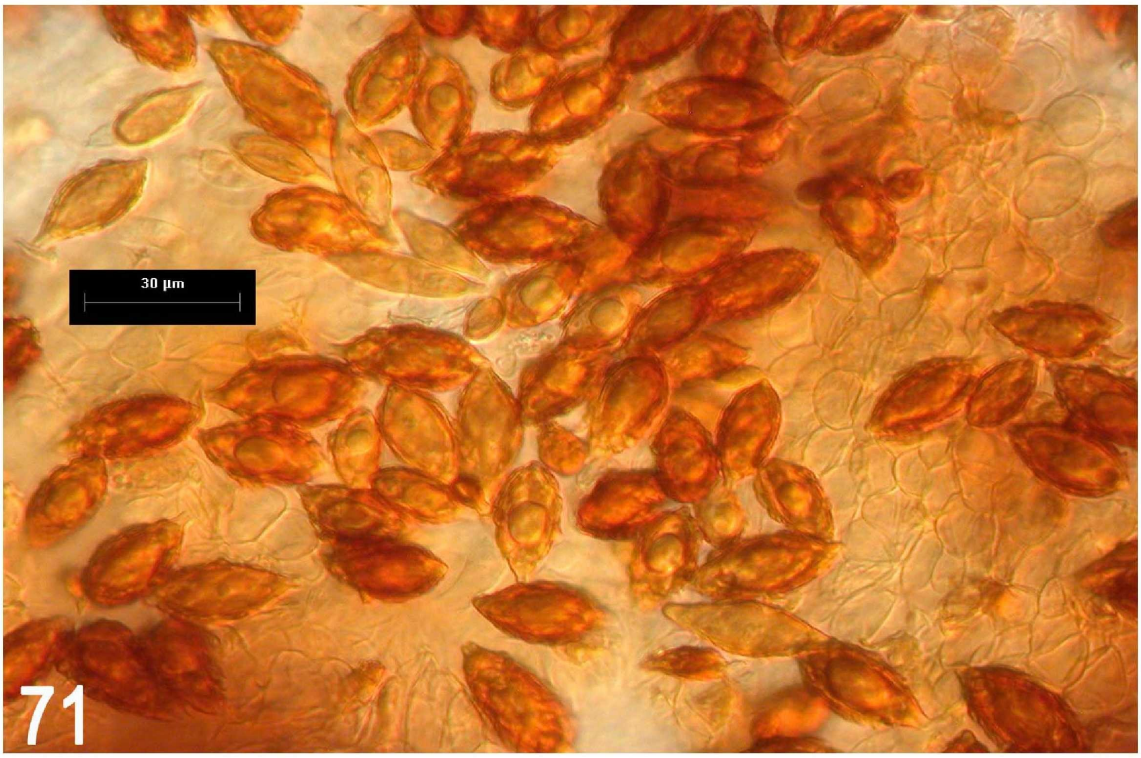
30 µm
71

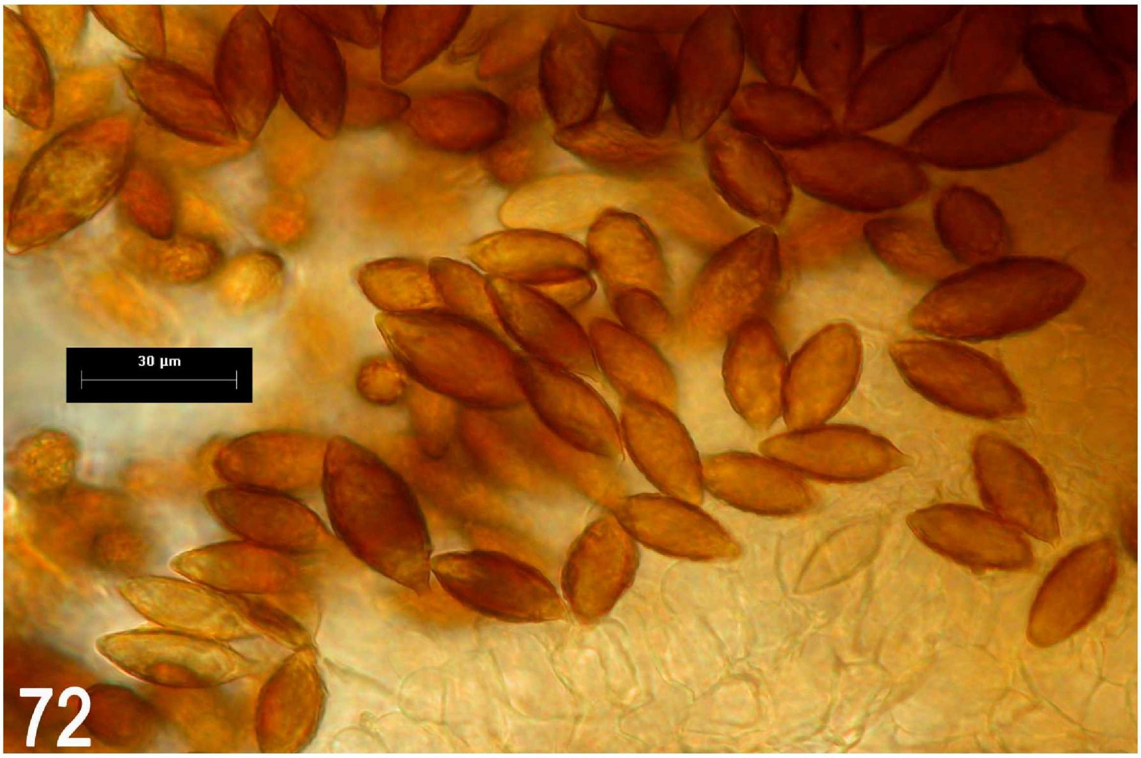
30 µm
72

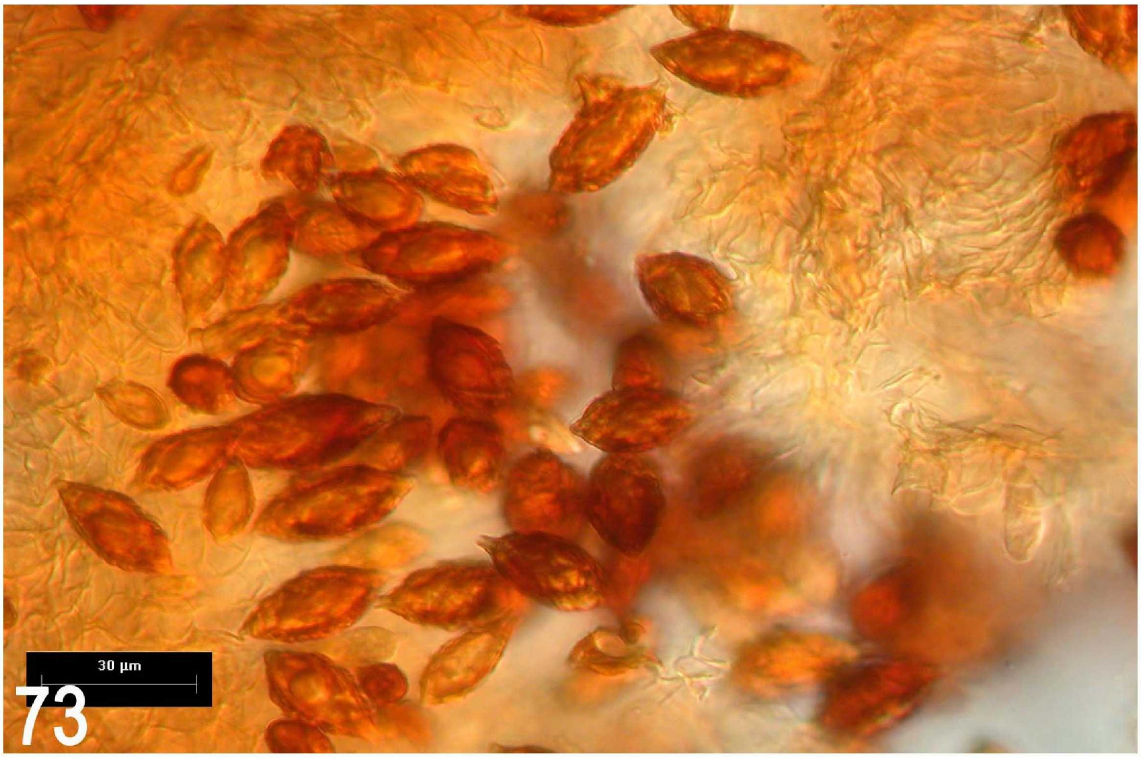
30 µm
73

Identification key to *Hymenogaster*

Hymenogaster huthii Stielow et al. 2010, sp. nov.

Spores: (16-) 22 (-29) x (9-) 13 (-21) µm in diameter; lanceolate, elongated, broadly fusoid, papillate, rounded at the apex (similar to *H. griseus* group like basidiospores).

Ornamentation: Verrucose, strongly wrinkled, infolded inwards; dark rusty-brown.

Basidiomata: Wrinkled, globose to subglobose, with cavities leading to the inner side in mature basidiomes; gouges present; ca. 1.5-2.5 cm in diameter.

Peridia: Plain brilliant white when young, reminding of *H. niveus* and *H. tener* basidiomata, with irregularly dispersed brownish spots in young and mature basidiomes.

Gleba: White, turning grey-brown, becoming brown-black at full maturity, columella absent.

Odour: Pleasant, cucumber like.

Ecology: The species is so far only known to be associated with *Tilia* species, *Corylus avellana* and *Alnus glutinosa* in habitats with limestone and basic soils.

Images: Basidiomata: No. 75. Basidiospores: No. 76-78. Two-spored-basidia: No. 77. Three-spored-basidia: No. 78

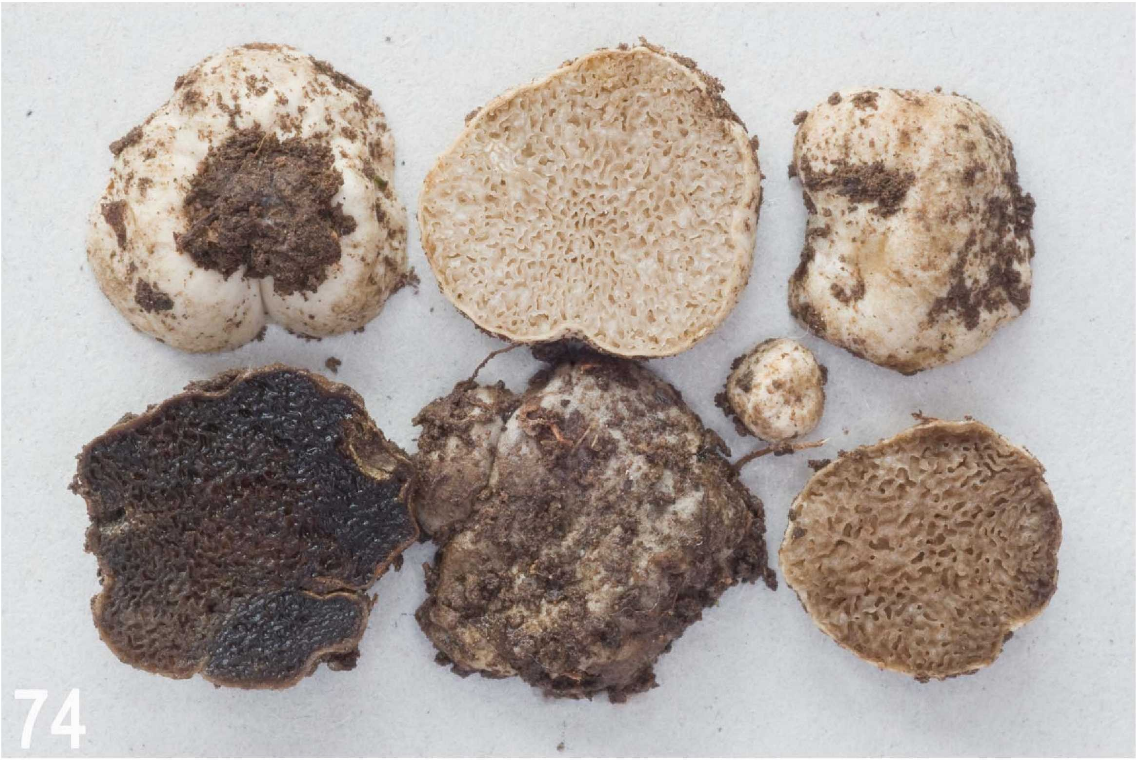
74

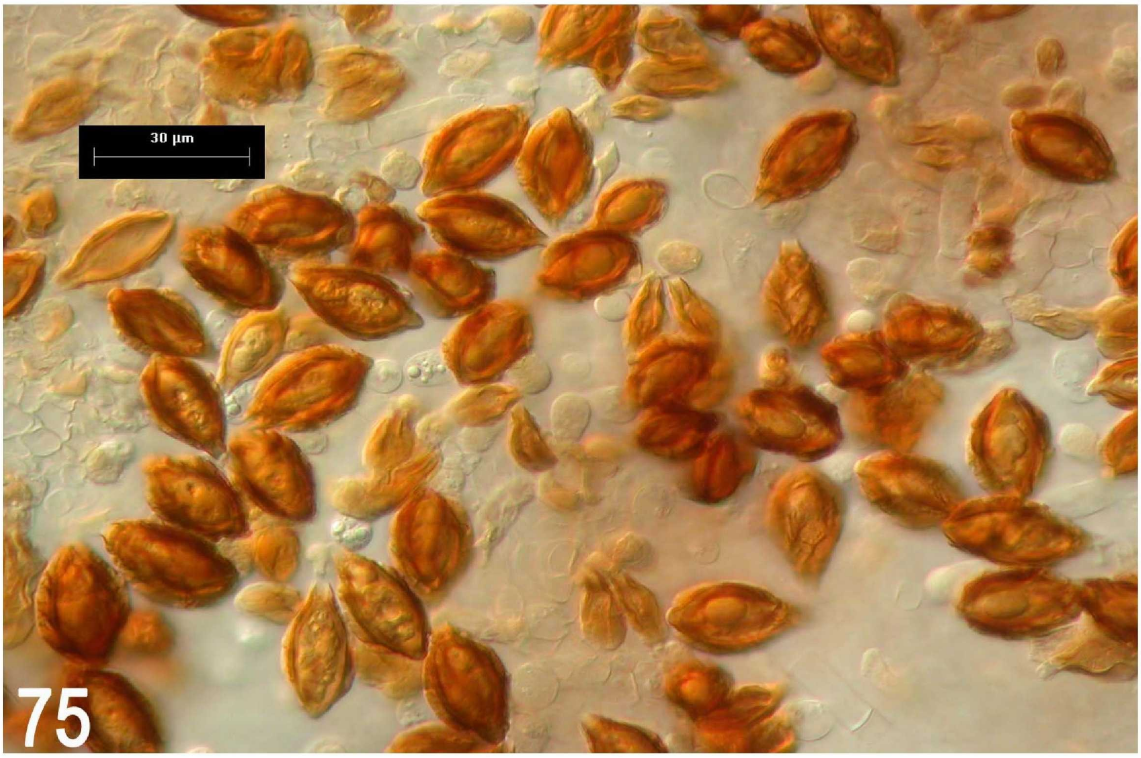
30 µm
75

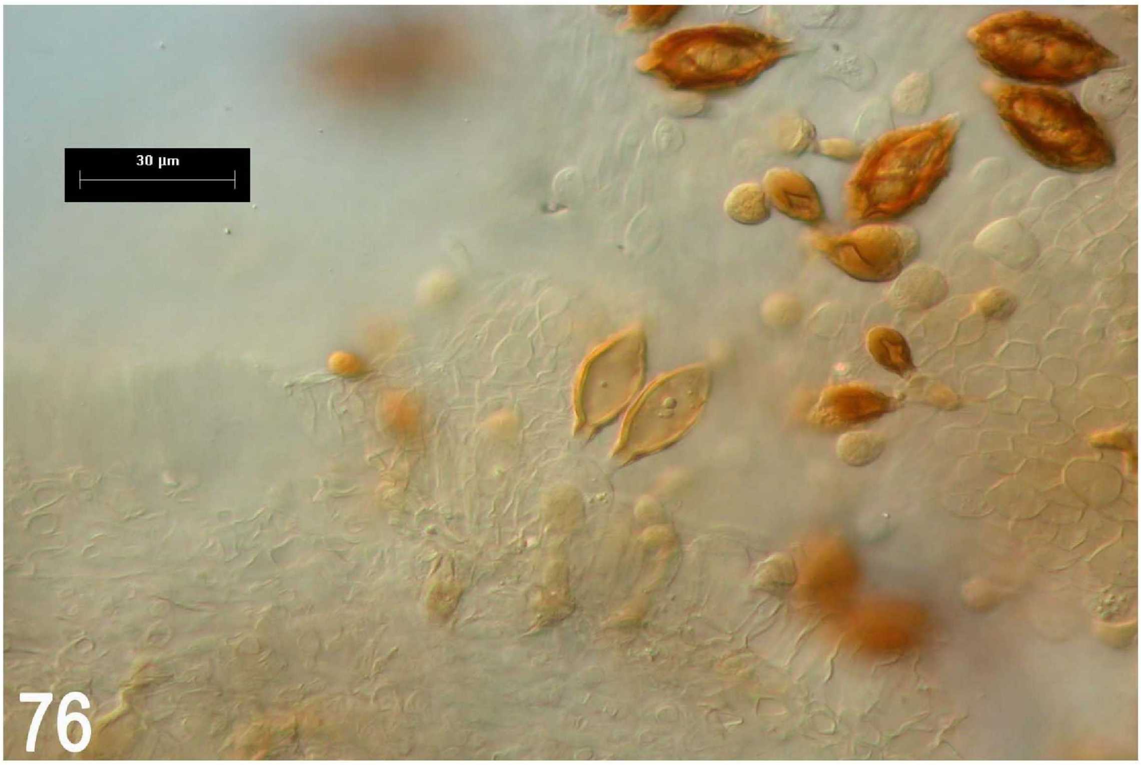
30 µm
76

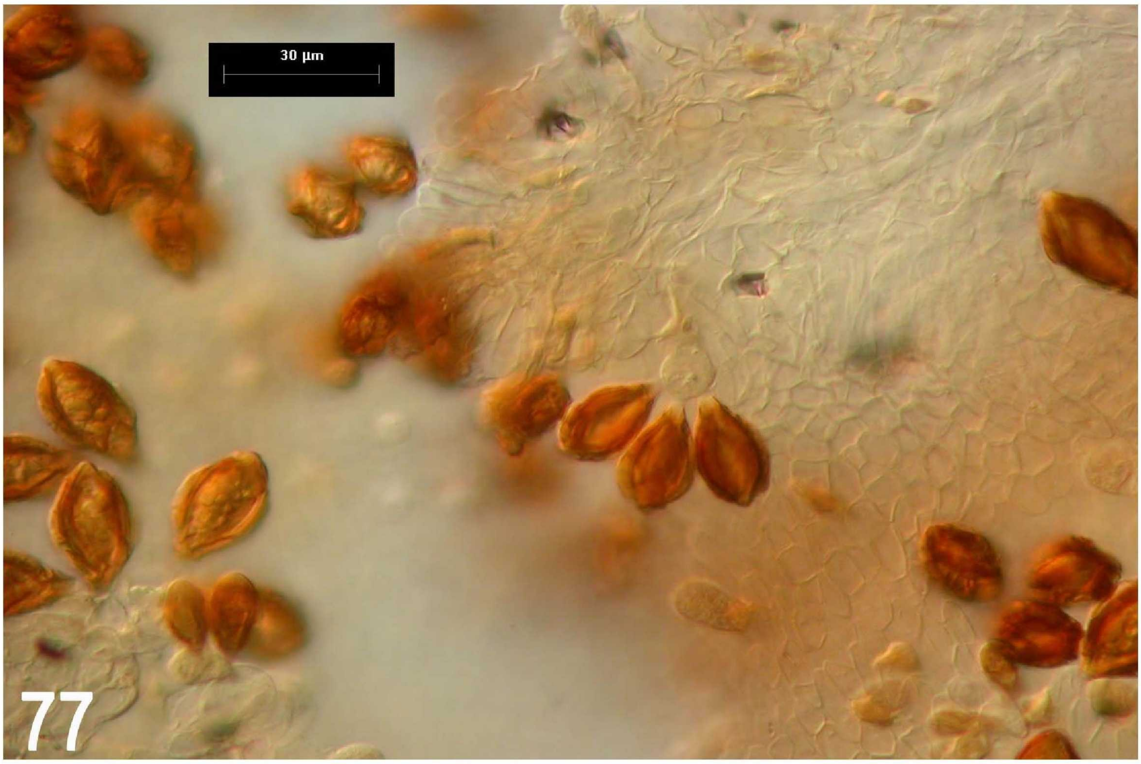
30 µm
77

Identification key to *Hymenogaster*

Hymenogaster griseus Vittad. 1831 emend. Stielow et al. 2010

Spores: (11-) 21 (-36) x (7-) 11 (-21) µm in diameter (quotient = 1.9); fusoid, mucronate, predominantly ellipsoid, but ovoid and lanceolate deviations are present.

Ornamentation: Verrucose, warted, wrinkled; colour brownish-yellowish to reddish, greenish tones absent.

Basidiomata: Globose to subglobose, tuberiform, lobated; ca. 1 - 4 (-6) cm in diameter.

Peridia: Variable in coloration, from pale brown to greyish-brownish-whitish in all possible graduations.

Gleba: Compressed, composed of irregularly arranged, labyrinthoid, small chambers; extremely variable in colouration, from distinct brown tones to black and to grey-white to brown-yellow, irrespective of the degree of maturity.

Odour: Variable, pleasant (reminding of lily) to unpleasant, from slightly unspecific to predominantly raphanoid with many graduations (often reminding of *Cortinarius*) to mature onions and garlic, like putrescent vegetables.

Ecology: The species is present under various deciduous and coniferous trees and soils, also in montane habitats.

Images: Basidiomata: No. 79-88. Basidiospores: No. 89-96.

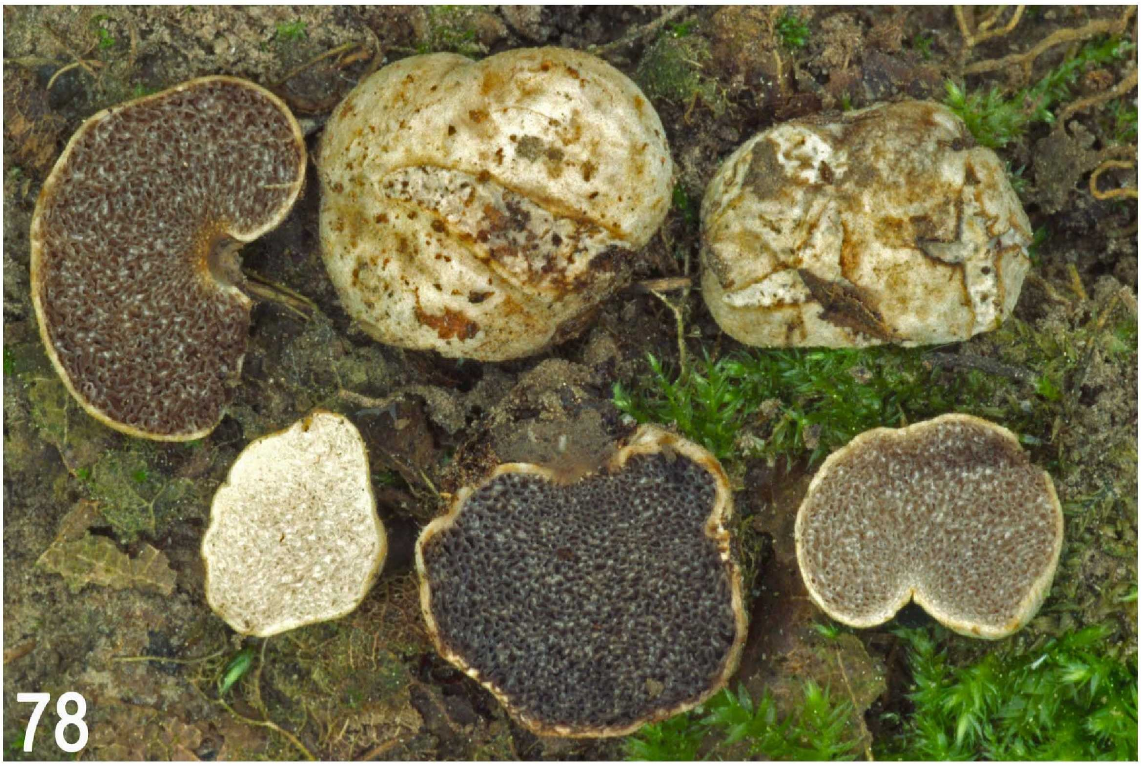
78

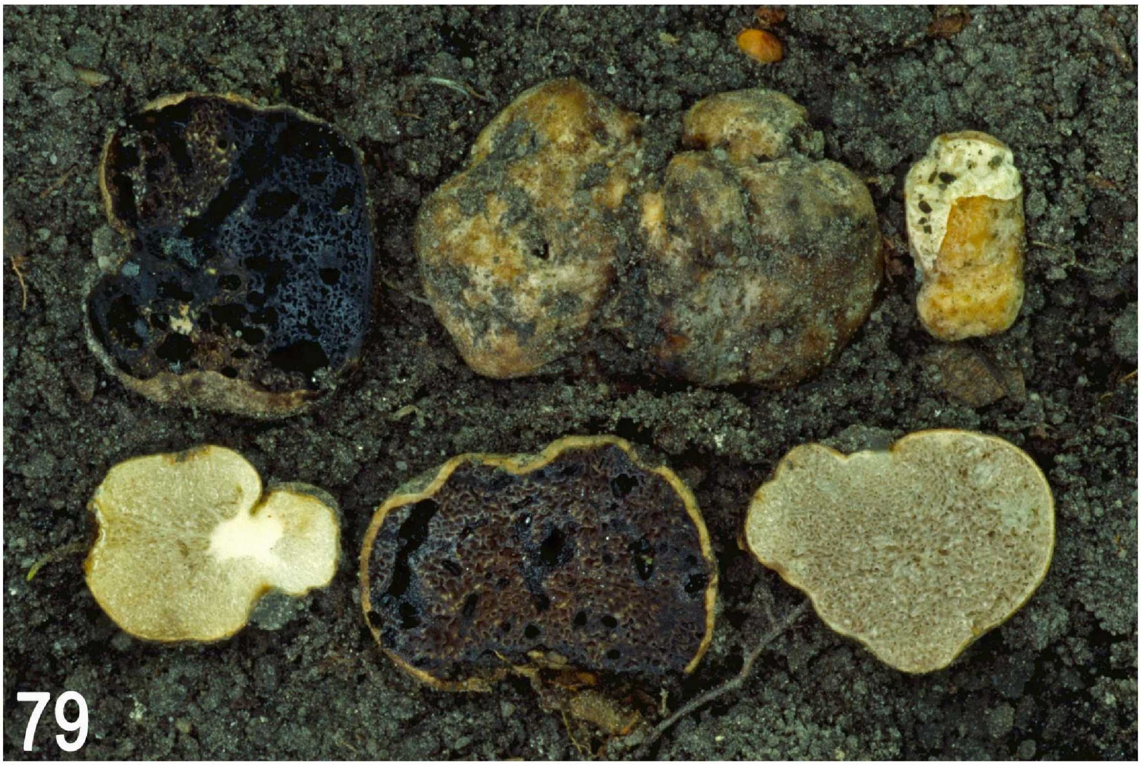
79

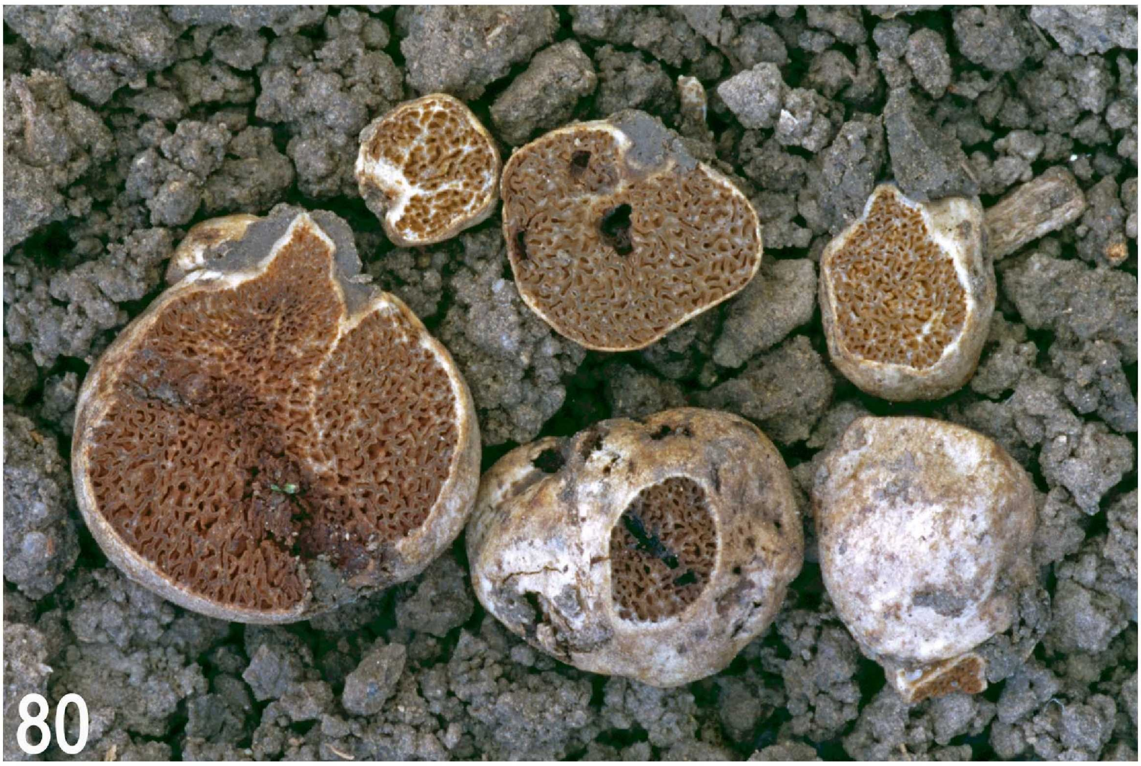
80

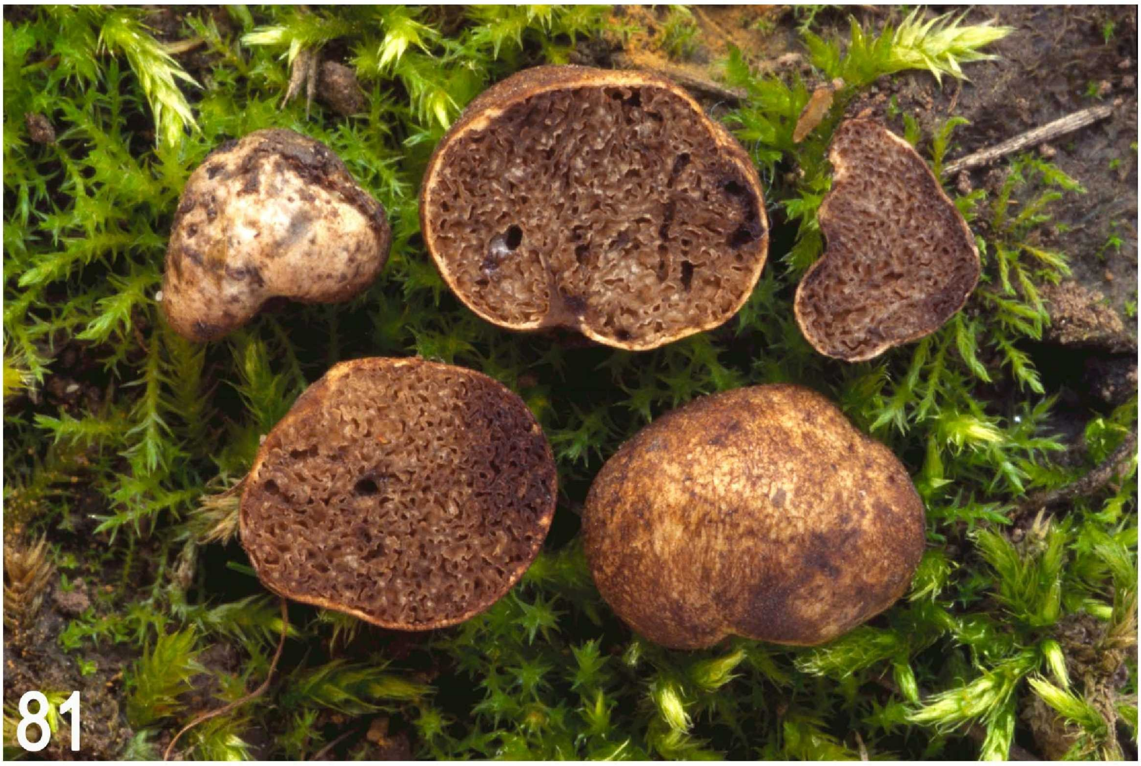
81

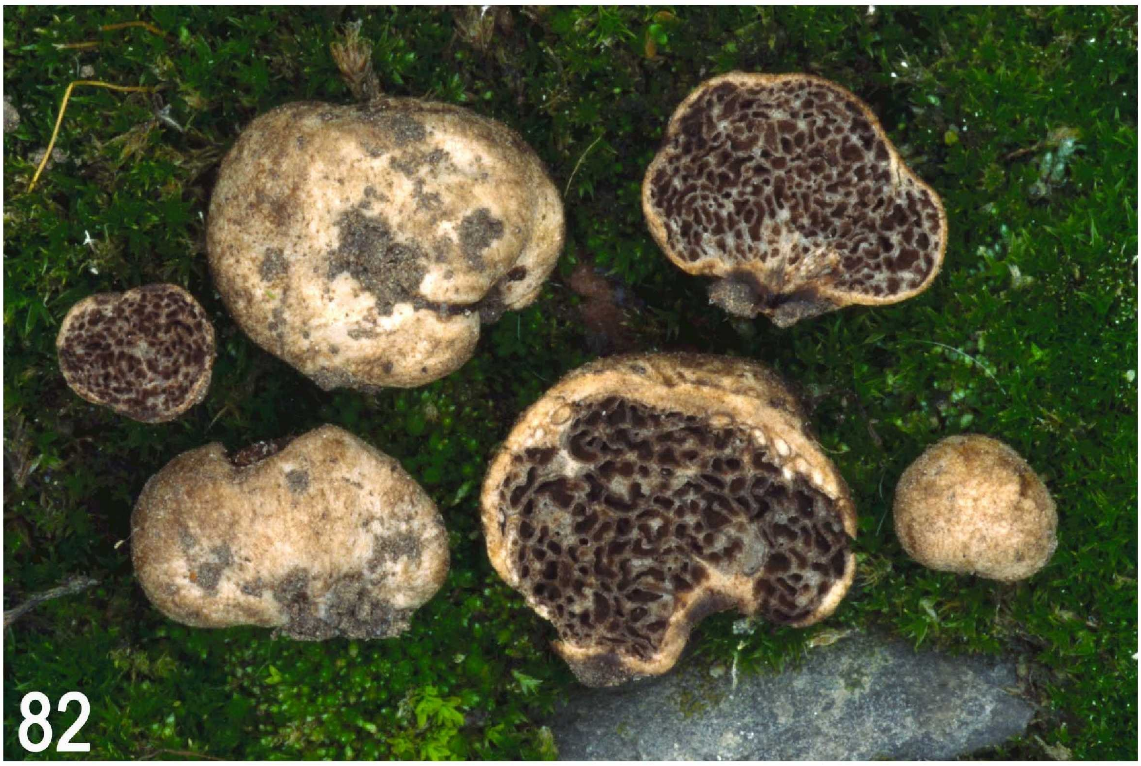

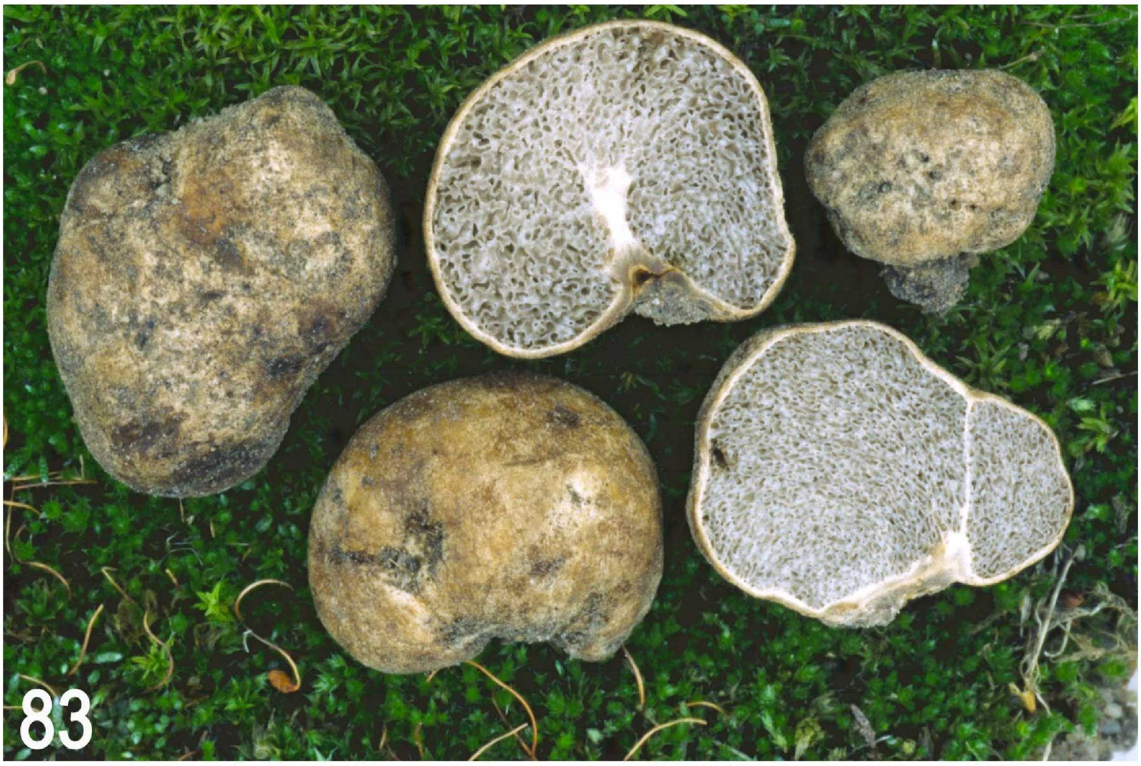

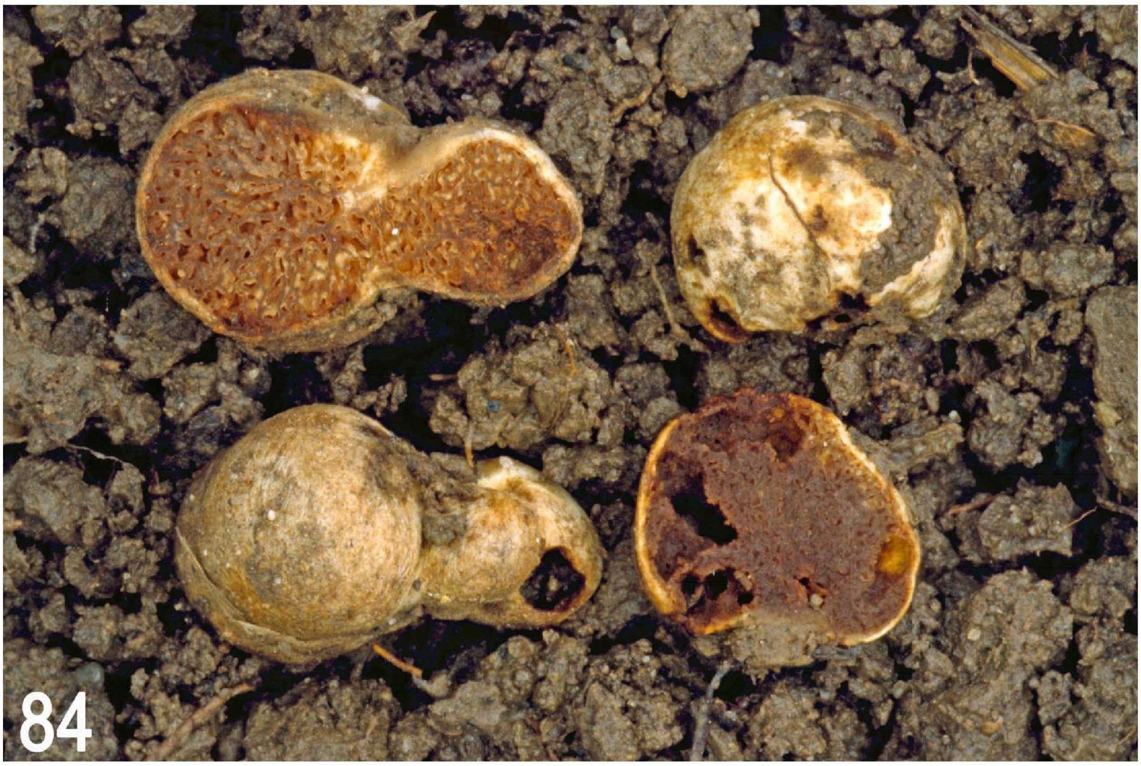

84

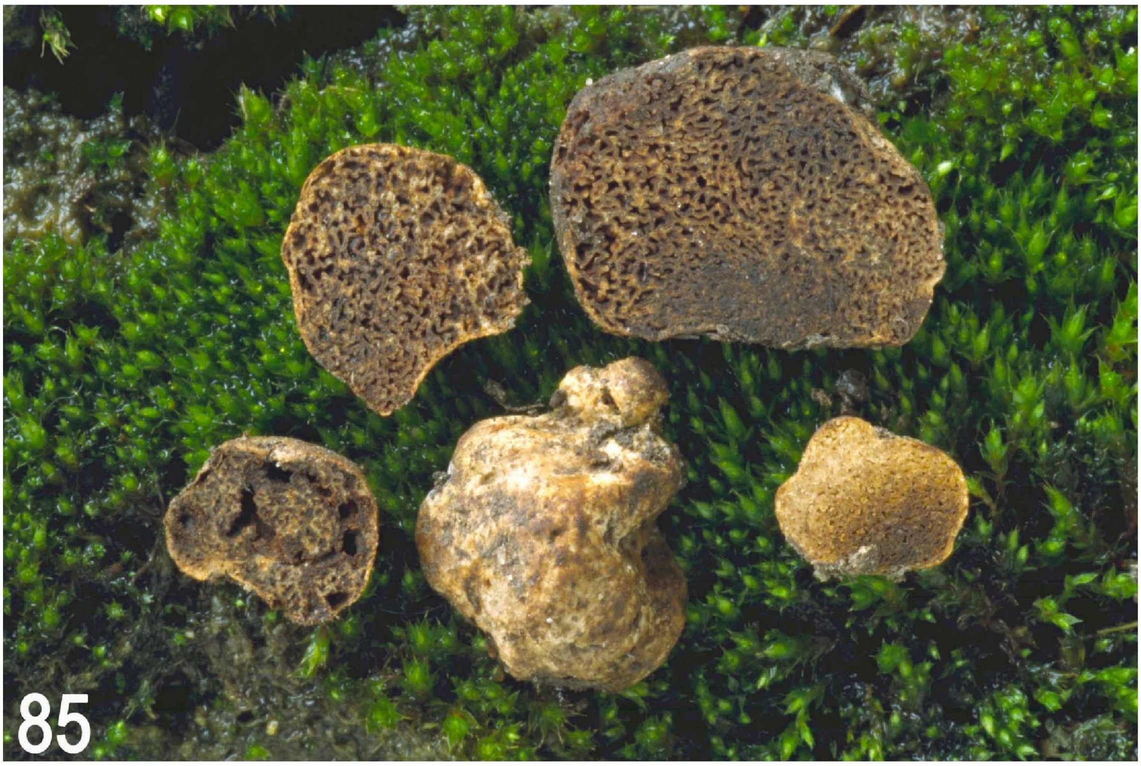

85

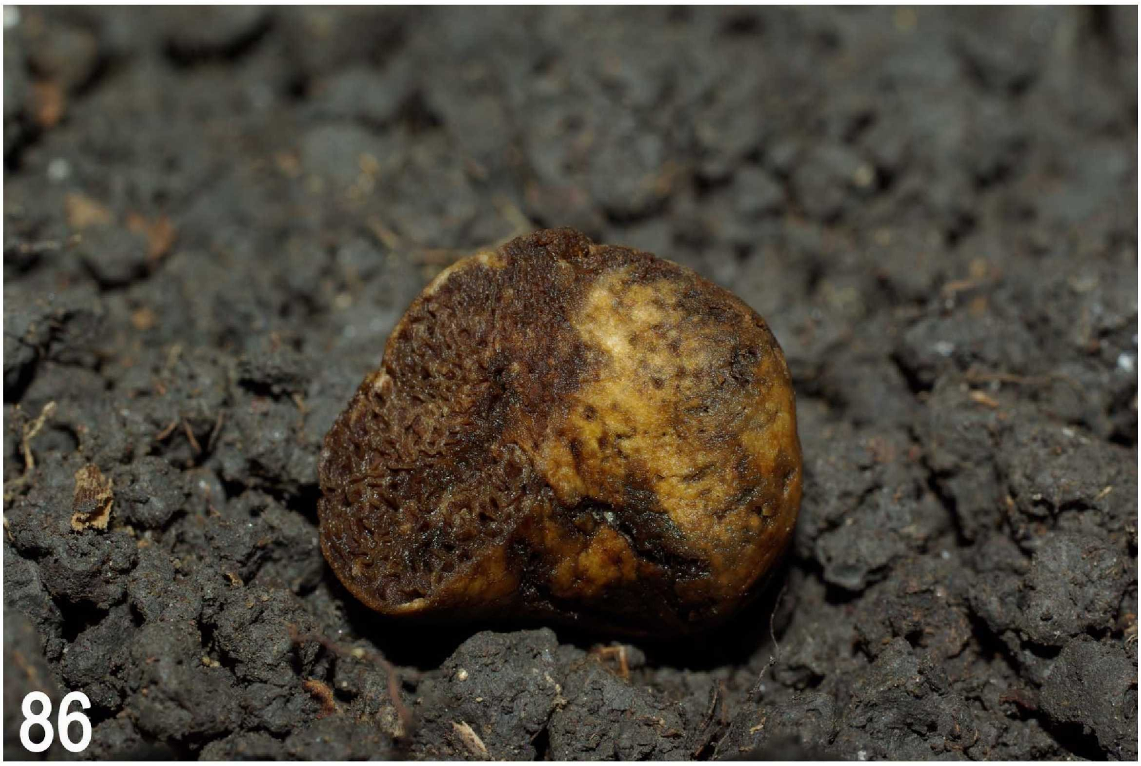
86

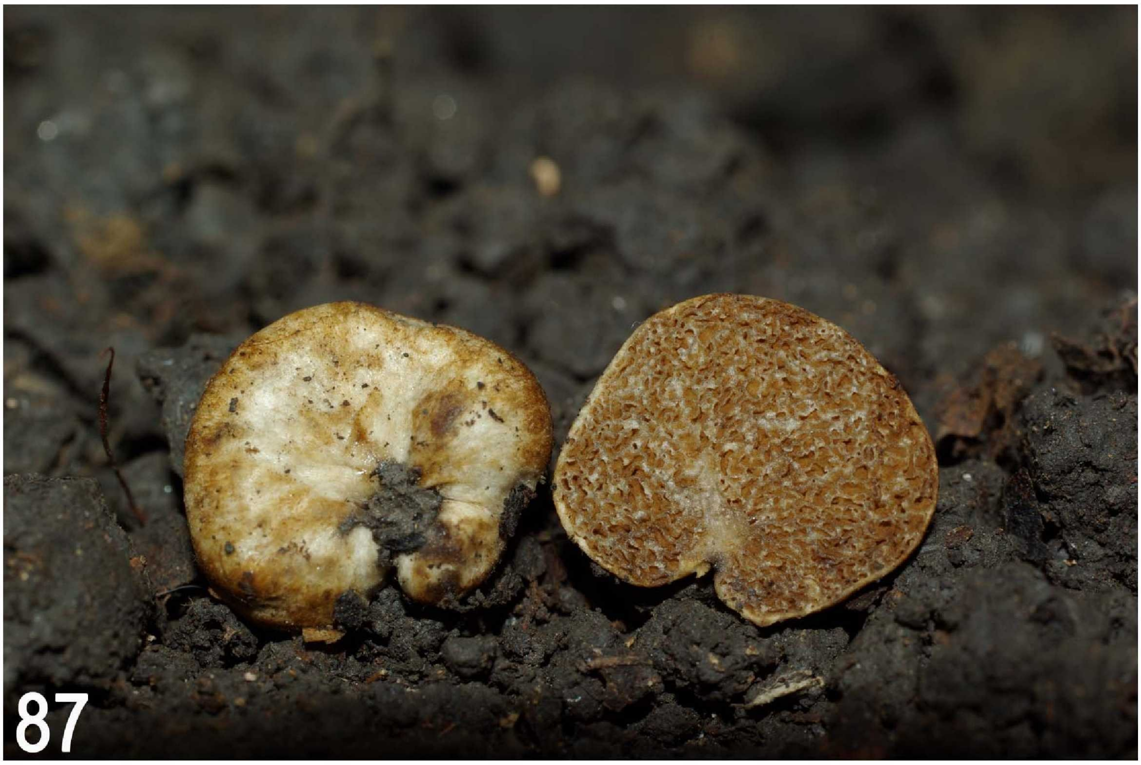
87

Identification key to *Hymenogaster*

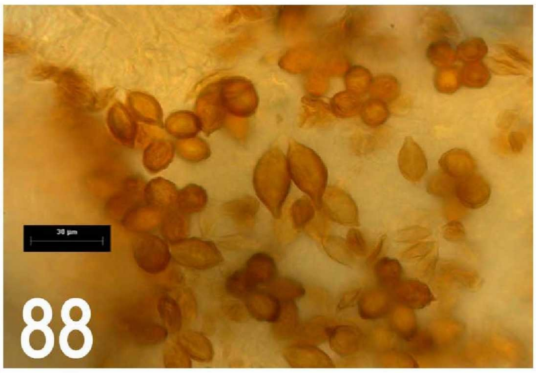

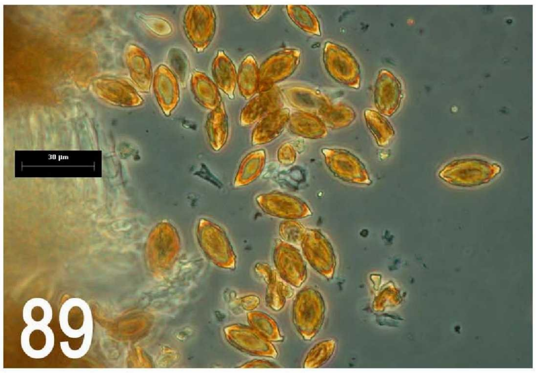

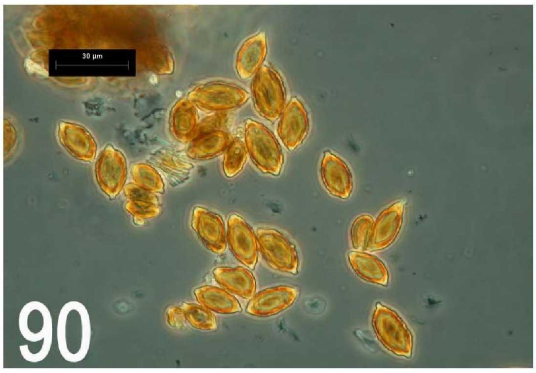

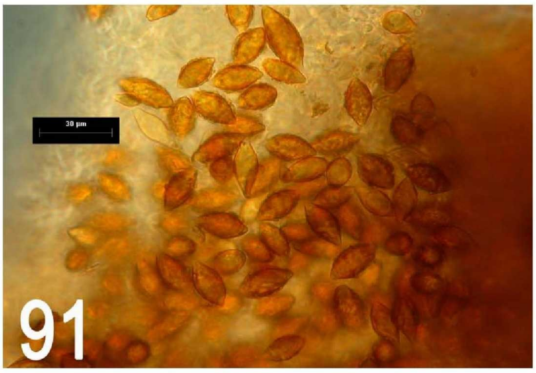

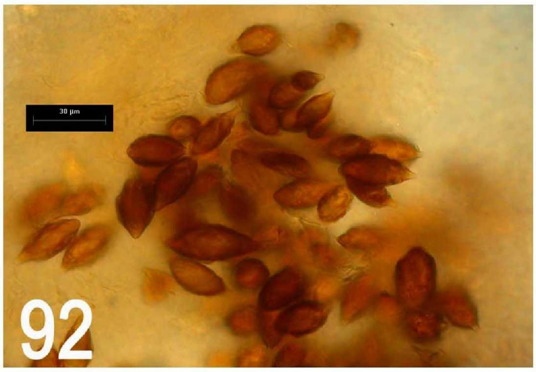

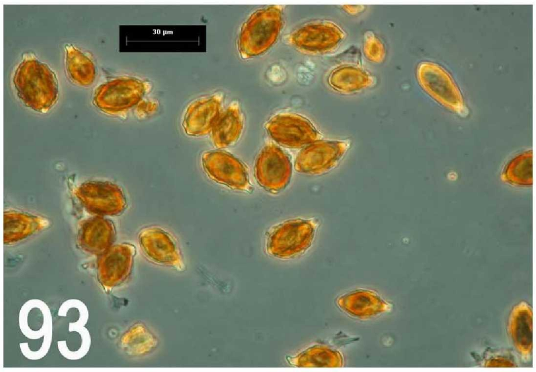

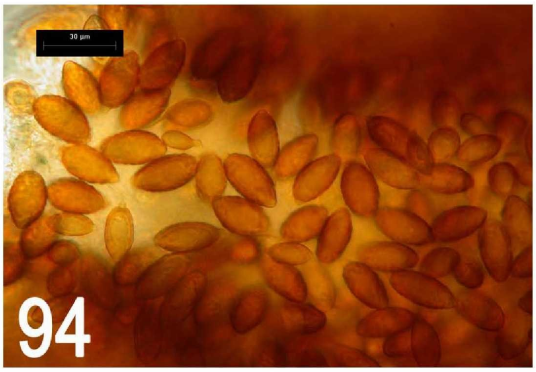

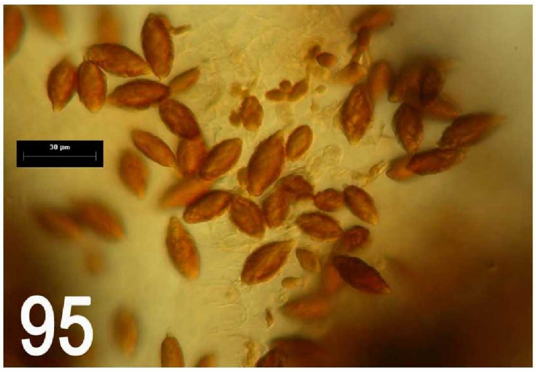

Identification key to *Hymenogaster*

Hymenogaster rehsteineri Bucholtz 1901 emend. Stielow et al. 2010

Spores: (13-) 20 (-30) x (8-) 11 (-15) µm in diameter; papillate to lanceolate shape predominant, but fusoid deviations always present.

Ornamentation: Verrucose, warted; colour brownish-yellowish, sometimes with very weak but distinct greenish tones (similar to *H. niveus*); loose perisporium constitutes two parallel walls, not always very evident but constantly present.

Basidiomata: Globose to tuberiform, sometimes elongated; ca. 1.5 -2 cm in diameter.

Peridia: Surface even, partially wrinkled, with visible hyphae; surface smooth, pale whitish-greyish when juvenile, turning ochraceous-yellowish-brownish at maturity

Gleba: Fragile, not compact, with small roundish chambers; greyish-brownish when juvenile, turning dark cinnamon-brown at maturity.

Odour: Pleasant in early juvenile stages, very unpleasant at maturity; rancid; when dry reminding of mature *Marasmius* species.

Ecology: The species is found under various deciduous trees and in different soils, often park-like habitats, also in montane habitats.

Taxonomic notes: *H. rehsteineri* is like *H. niveus* a difficult species complex with at least three cryptic species. One *H. rehsteineri* cryptic species could not be subjected to an intensive morphological analyses, as only two specimens were available. The second cryptic species represents the typical *H. rehsteineri*, with the main characters begin a pale ochraceous, yellowish or brownish basidiomata and lanceolate, strongly verrucose-thorny basidiospores.

Identification key to *Hymenogaster*

Images: Basidiomata: No. 97, 99. Basidiospores: No. 43, 98, 100, 101.

- Cryptic species one (= cluster 14): spores (13-) 21 (-24) x (7-) 12 (-15) µm
- (images 99-101).
- Cryptic species two (= cluster 8): spores (14-) 20 (-30) x (8-) 10 (-15) µm (images 43, 97, 98).

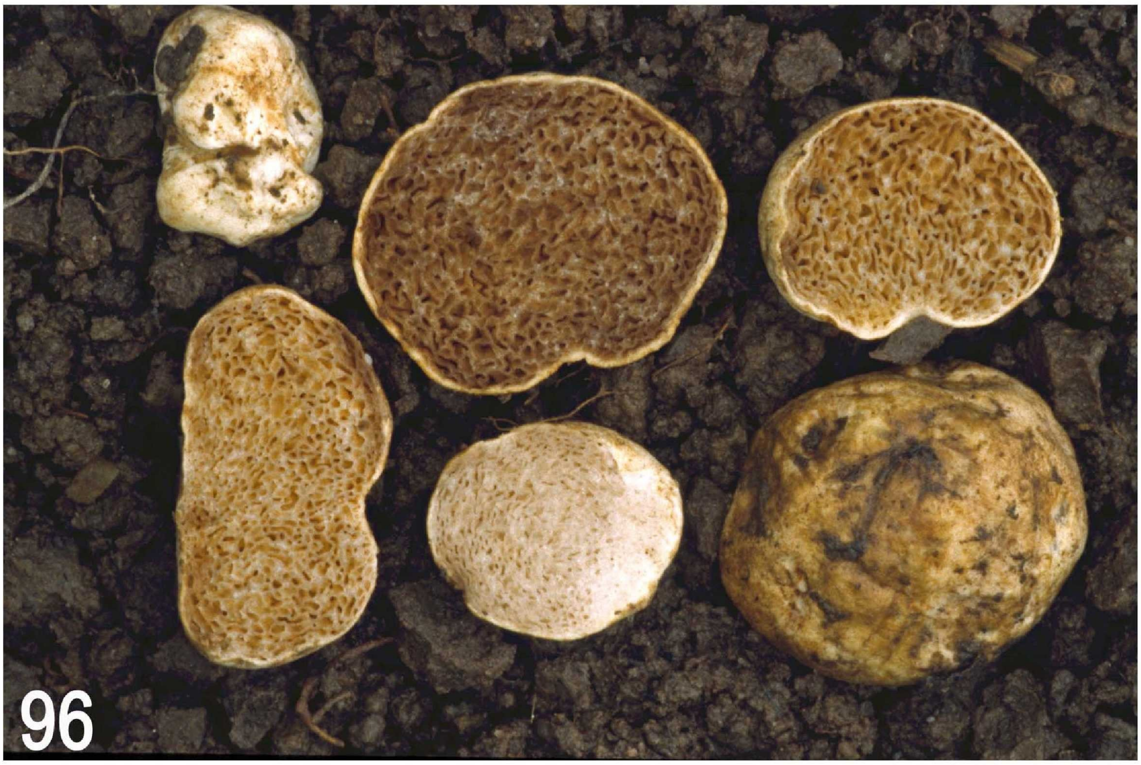
96

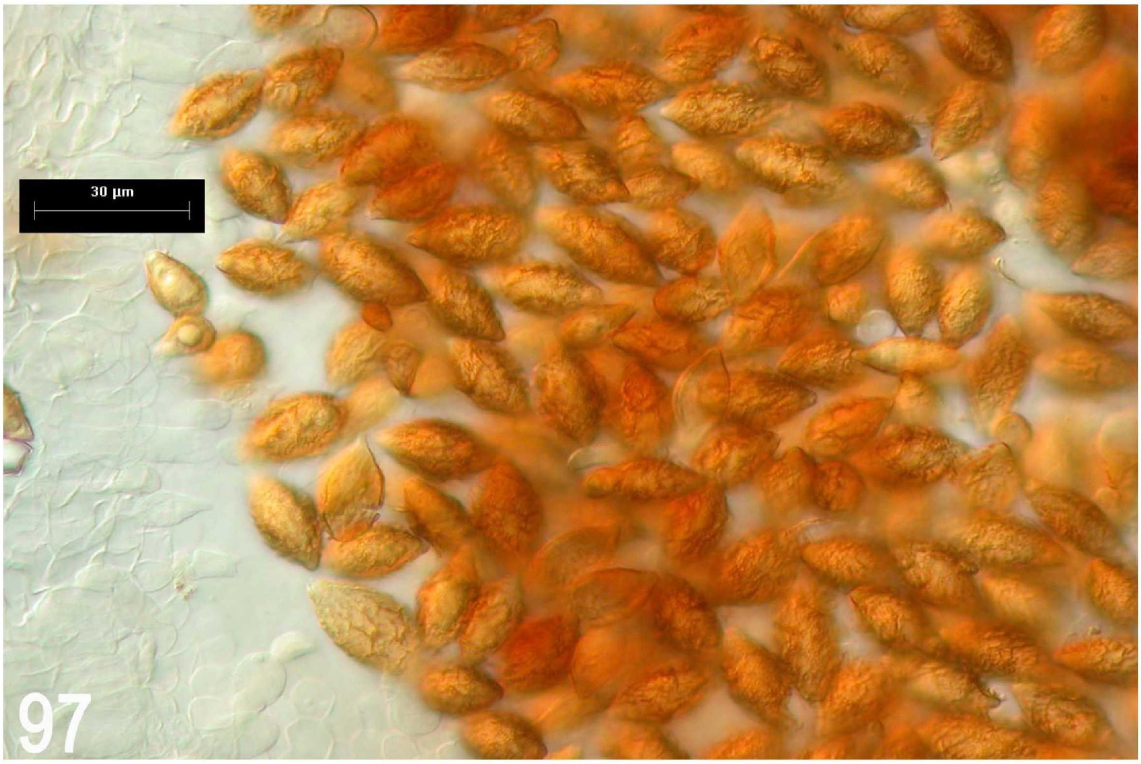
30 µm
97

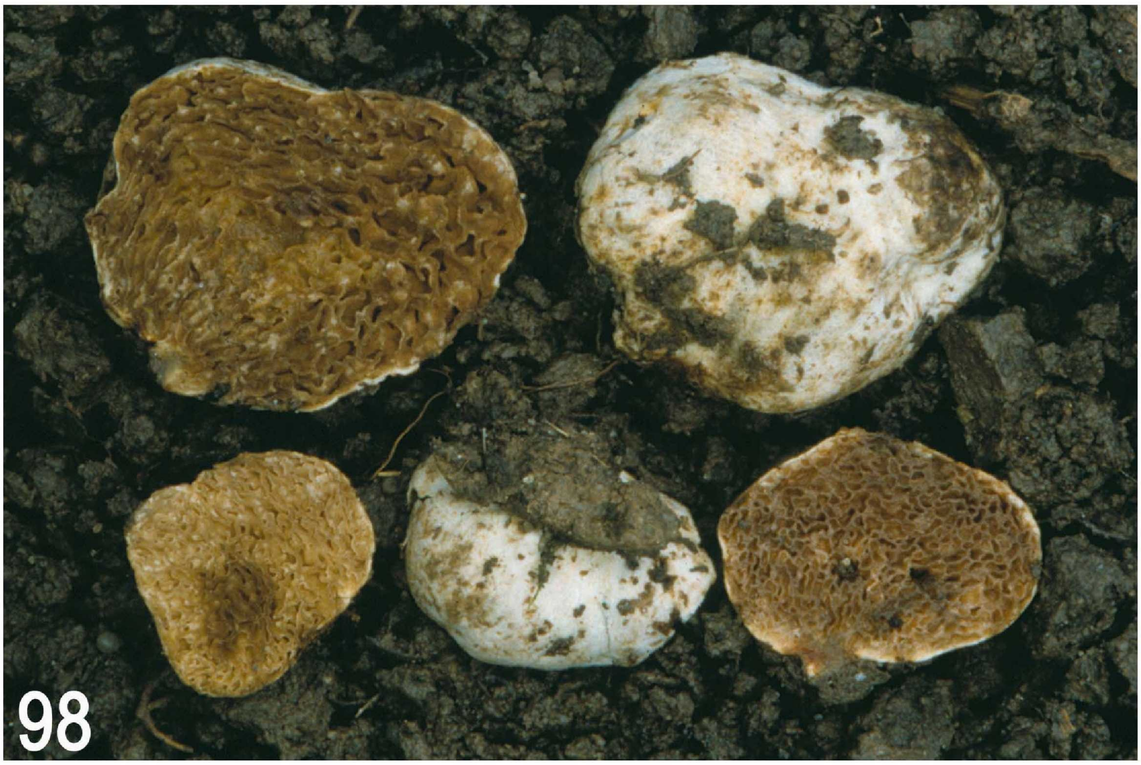

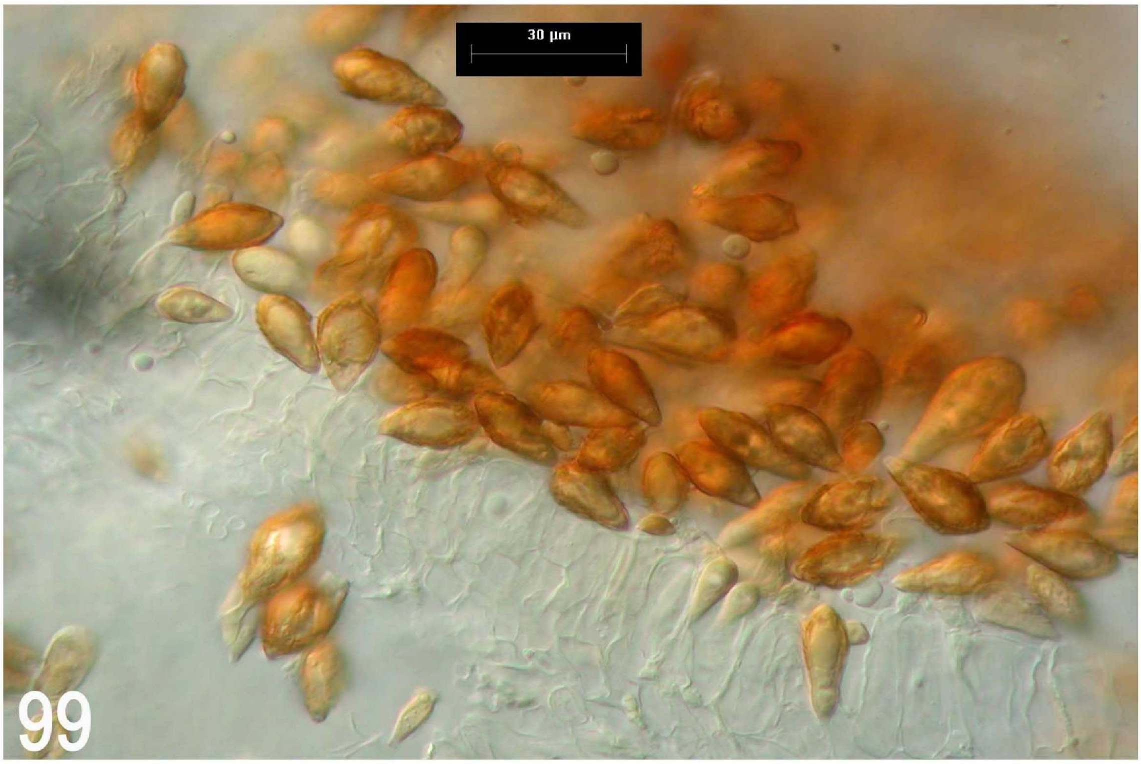
30 µm

Identification key to *Hymenogaster*

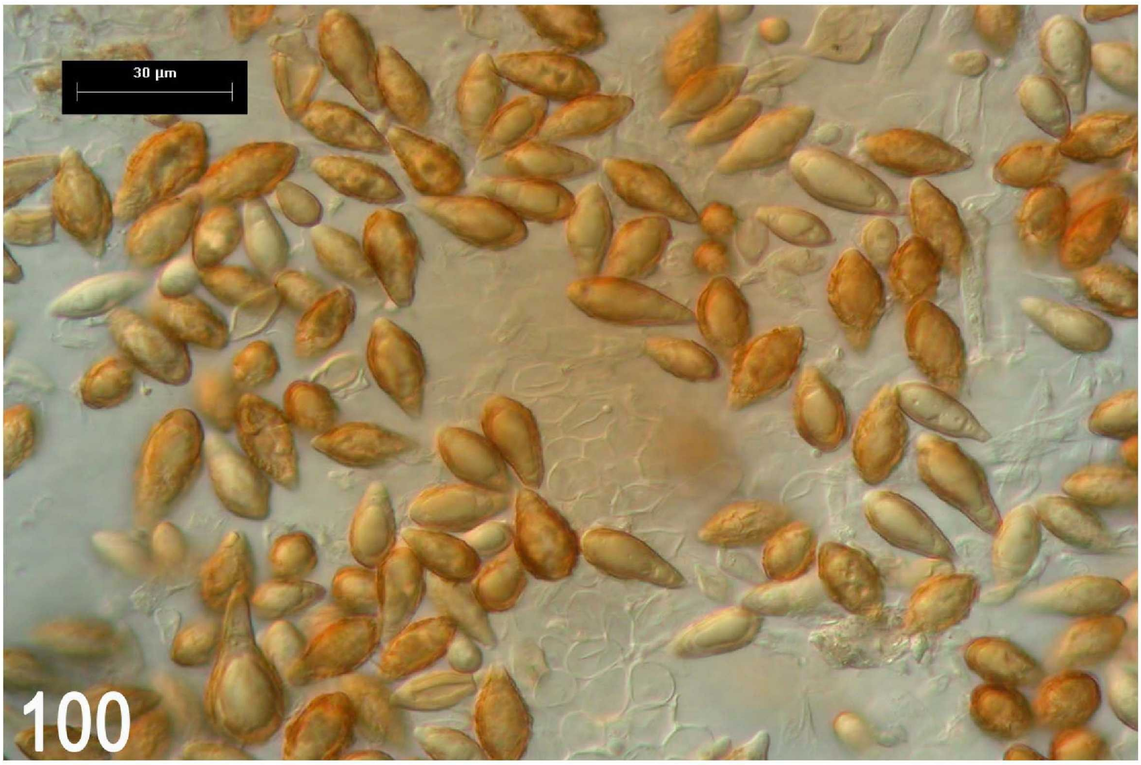

Supplement: File S1 — Taxonomic descriptions based on the 142 dried and fresh basidiomata collected by the authors ( Table 1 ) and illustrated identification key for the Central European Hymenogaster species based on the revised classification (PDF format). (PDF) [file pone.0015614.s001.pdf]
